# Sulfoximine Assisted C–H Activation and Annulation via Vinylene Transfer: Access to Unsubstituted Benzothiazines

**DOI:** 10.3390/molecules28135014

**Published:** 2023-06-27

**Authors:** Koneti Kondalarao, Somratan Sau, Akhila K. Sahoo

**Affiliations:** School of Chemistry, University of Hyderabad, Hyderabad 500046, India; konetikondalarao123@gmail.com (K.K.); somratan.somu@gmail.com (S.S.)

**Keywords:** vinylene transfer, C–H activation, redox neutral, sulfoximine

## Abstract

In this study, we report the synthesis of unsubstituted 1,2-benzothiazines through a redox-neutral Rh(III)-catalyzed C–H activation and [4+2]-annulation of S–aryl sulfoximines with vinylene carbonate. Notably, the introduction of an N-protected amino acid ligand significantly enhances the reaction rate. The key aspect of this redox-neutral process is the utilization of vinylene carbonate as an oxidizing acetylene surrogate and an efficient vinylene transfer agent. This vinylene carbonate enables the cyclization with the sulfoximine motifs, successfully forming a diverse array of 1,2-benzothiazine derivatives in moderate to good yields. Importantly, this study highlights the potential of Rh(III)-catalyzed C–H activation and [4+2]-annulation reactions for the synthesis of optically pure 1,2-benzothiazines with high enantiomeric purity.

## 1. Introduction

**Figure 1:**
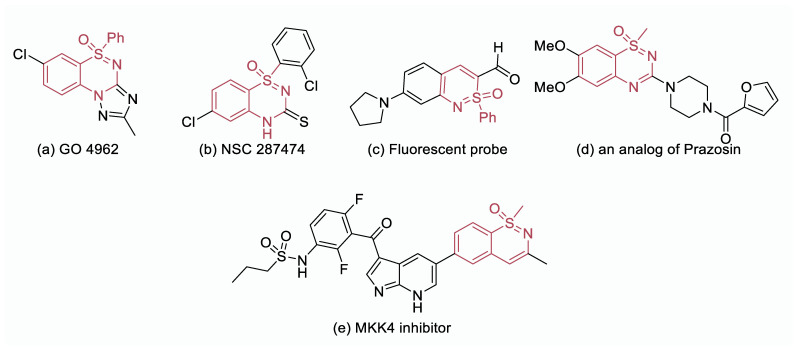
Biologically active compounds containing sulfoximine scaffold.

In recent years, the field of medicinal chemistry has experienced a remarkable expansion, particularly in the exploration of sulfur stereogenic complex scaffolds, which possess unique physicochemical properties. One prominent class of such scaffolds is S(VI)-stereogenic sulfoximines, which have attracted considerable attention due to their versatility and adaptability in synthesizing innovative molecular structures. By incorporating aryl/alkyl groups into sulfoximine frameworks, the complexity and chemical diversity of compounds can be significantly enhanced. This is evident from the wide range of biologically active compounds derived from sulfoximines and benzothiazine moieties [1,2,3,4]. An excellent example of the pharmacological potential of sulfoximines and benzothiazines is the compound GO4962 (Figure 1a), which acts as a partial agonist for benzodiazepine receptors, exhibiting anxiolytic and anticonvulsive properties [5]. Another noteworthy compound, NSC 287474 (Figure 1b), has emerged as a promising inhibitor of reverse transcriptase, offering protection to lymphocytes against HIV [6,7]. Furthermore, a fluorescent probe for the detection of Cu(II) has been developed based on sulfoximine and benzothiazine molecular entities (Figure 1c) [8]. Interestingly, analogs of prazosin, an antihypertensive agent, have also been synthesized using sulfoximines, demonstrating their potential in the development of therapeutic agents (Figure 1d) [9]. Additionally, a sulfoximine-based compound has been identified as an inhibitor of mitogen-activated protein kinase 4 (MKK4) (Figure 1e), showcasing the diverse pharmacological properties that can be achieved through the manipulation of sulfoximines and benzothiazine molecular entities. Overall, the rich pharmacological properties and wide-ranging applications of sulfoximines and benzothiazines make them valuable building blocks for the synthesis of innovative and biologically relevant compounds in medicinal chemistry.

**Scheme 1:**
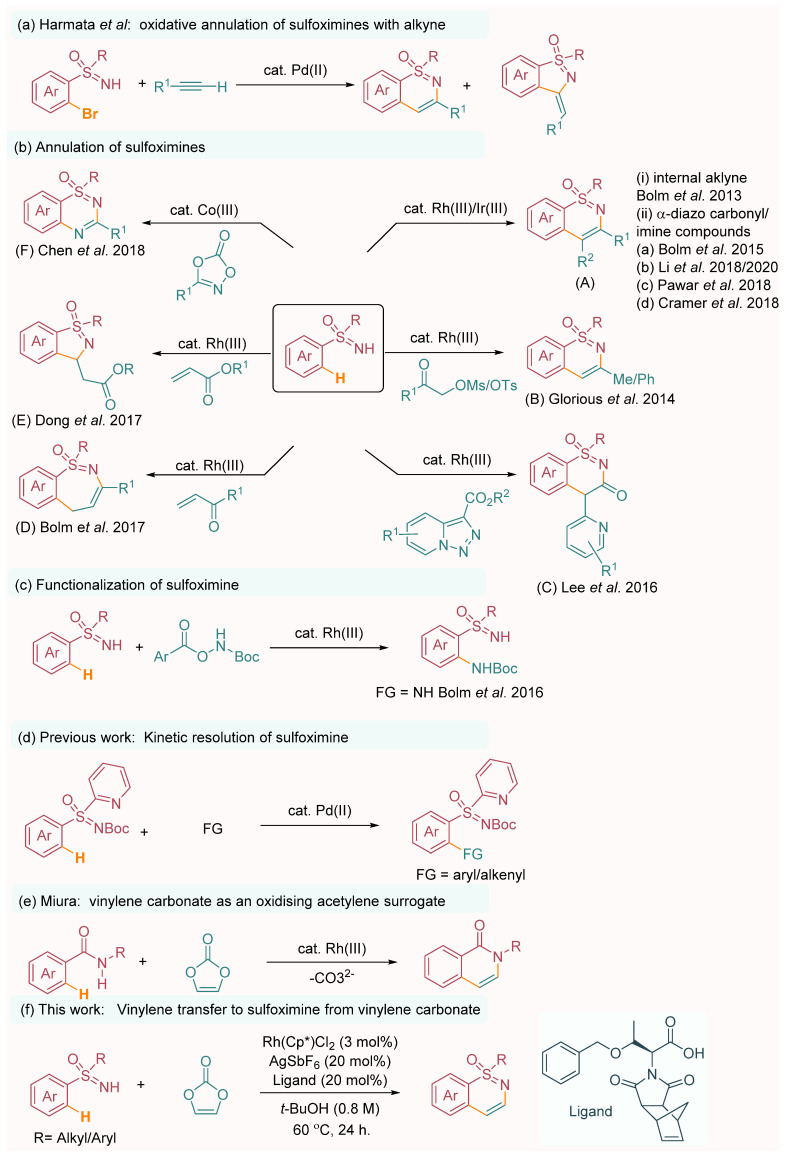
Synthetic methods for annulation of sulfoximines. (**a**) Harmata’s work [10]; (**b**)-**A**-(**i**) Bolm’s work [11]; (**b**)-**A**-(**ii**-**a**)-Bolm’s work [12]; (**b**)-**A**-(**ii**-**b**)-Li’s work [18]; (**b**)-**A**-(**ii**-**c**)-Pawar’s work [19]; (**b**)-**A**-(**ii**-**d**)-Cramer’s work [20,21]; (**b**)-**B**-Glorious work [13]; (**b**)-**C**-Lee’s work [14]; (**b**)-**D**-Bolm’s work [15]; (**b**)-**E**-Dong’s work [16]; (**b**)-**F**-Chen’s work [17]; (**c**)-Bolm’s work [22]; (**d**)-Previous work [23]; (**e**)-Miura’s work [24]; (**f**)-This work.

Recently, sulfoximines have also emerged as leading precursors for a wide range of chemical transformations, enabling the generation of complex sulfur-bearing scaffolds. One particularly notable innovation in this field is the palladium-catalyzed synthesis of 1,2-benzothiazines, which was introduced by Harmata and coworkers (Scheme 1a) [10]. This method utilizes terminal alkynes as the preferred coupling partners, offering a novel approach to accessing diverse benzothiazine derivatives. In 2013, Bolm’s research group demonstrated the rhodium-catalyzed C–H activation/cyclization of sulfoximines with alkynes, resulting in the construction of heterocyclic cores bearing sulfoximine functionalities (Scheme 1b-A-i) [11]. Building upon this work, two years later, the same group reported another significant advancement with a rhodium-catalyzed directed migratory carbene insertion of conventional NH-sulfoximines into aromatic C–H bonds. Subsequent regioselective dehydrative ring closures facilitated the synthesis of aryl-fused sulfoximines (Scheme 1b-A-ii(a)) [12]. In 2014, Glorius et al. successfully employed α-MsO/TsO ketones as C(sp^3^)-based electrophiles and oxidized alkyne equivalents in Rh(III)-catalyzed redox-neutral annulations to desired 3-methyl/aryl 1,2-benzothiazines (Scheme 1b-B) [13]. In 2016, Lee’s research group made a significant contribution by reporting the synthesis of 1,2-benzothiazine skeletons derived from NH-sulfoximine through the use of a Rh(III) catalyst in the presence of heterocyclic coupling partners (Scheme 1b-C) [14]. In 2017, the Bolm group made a significant contribution by reporting the synthesis of 1,2-benzothiazepine utilizing α,β-unsaturated carbonyl compounds, thereby showcasing the effectiveness of the Rh(III) catalyst in facilitating the formation of 1,2-benzothiazepine (Scheme 1b-D) [15]. Similarly, the Dong group also made noteworthy progress by introducing a method for synthesizing 1,2-benzoisothiazoles using α, β-unsaturated esters under the influence of the Rh(III) catalyst (Scheme 1b-E) [16]. In 2017, the Chen group made a significant contribution by reporting a Co(III)-catalyzed C–H activation/double C–N bond formation reaction (Scheme 1b-F) [17]. This reaction involved the utilization of free NH-sulfoximines and 1,4,2-dioxazol-5-ones as substrates, and it was conducted under the influence of microwave irradiation to construct structurally orchestrated thiadiazine 1-oxide derivatives.

The Pawar group made an important contribution by reporting the utilization of the Ir(III) catalyst for the synthesis of 1,2-benzothiazines through C–H activation of sulfoximine with α-diazo carbonyl compounds (Scheme 1b-A-ii(c)) [19]. In addition, the Bolm group presents a novel approach for the synthesis of heterocyclic thiadiazine 1-oxide by employing Rh(III) catalysis to facilitate the amination of sulfoximine. The amidating agent utilized in this process is *tert*-butyl (2,4,6-trichlorobenzoyl)oxycarbamate (Scheme 1c) [22]. One notable advantage of sulfoximine is its sulfur atom, which possesses four coordination centers, making it a prochiral or chiral entity. Recognizing this unique feature, several research groups have embarked on developing asymmetric C–H activation methods specifically targeting sulfoximines. A significant breakthrough in this area was reported by Li and co-workers, who described an unprecedented enantiodivergent [4+2] annulative coupling of sulfoximines with α-diazocarbonyl compounds. This groundbreaking approach relied on the use of a chiral Rh(III) catalyst to facilitate a desymmetrizing C–H activation process (Scheme 1b-A-ii(b)) [18]. Notably, Cramer and his research group made significant contributions to this field. Cramer’s work focused on the development of a chiral rhodium catalyst-assisted strategy for the synthesis of chiral 1,2-benzothiazines. By utilizing α-diazo carbonyl compounds as reactants and sulfoximines as starting materials, the team successfully achieved the formation of structurally diverse 1,2-benzothiazine derivatives. One key aspect of their approach was the implementation of desymmetrization [20] and kinetic resolution [21] processes (Scheme 1b-A-ii(d)), which enabled the generation of enantioenriched products. To achieve high levels of enantioselectivity, Cramer employed a chiral rhodium catalyst in combination with an N-diprotected chiral amino acid as an additive. The chiral catalyst played a crucial role in selectively activating the C–H bond of the sulfoximine, while the chiral additive facilitated the efficient differentiation of the two enantiomeric sulfoximine substrates, leading to enantioenriched 1,2-benzothiazine products. The Shi group recently reported the enantioselective annulation of sulfoximines using chiral carboxylic acids as a chiral source. This study employed Ir(III) [25] and Ru(III) [26,27] catalysts to achieve the desired enantioselectivity. Building on this research, our group made a significant contribution by reporting a palladium-catalyzed enantioselective arylation of sulfoximines (Scheme 1d) [23]. In our study, we utilized BOC-protected amino acids as a chiral source, resulting in high levels of enantioselectivity. More recently, Liu’s group addressed another challenging aspect of sulfoximine chemistry by developing a Rh(III)-catalyzed C–H annulation and cyclization strategy. Fused isochromeno-1,2-benzothiazines were successfully synthesized by employing 4-diazoisochroman-3-imines as coupling partners with sulfoximines [28]. In parallel to these advancements, significant progress has been made in the field of C–H activation, enabling the construction of various C–C, C–N, C–O, C–S, and C–X bonds [19,29,30,31,32,33,34,35,36,37,38,39,40,41,42]. Despite these remarkable achievements, the non-substituted vinylene annulation of S-aryl sulfoximines remains a formidable challenge. The main hurdle lies in the requirement for a suitable acetylene surrogate that can effectively produce vinylene-fused compounds, which adds complexity to the reaction design and necessitates the development of innovative synthetic approaches. The utilization of acetylene itself as a reactant poses challenges due to its gaseous nature, which requires specialized equipment and introduces safety hazards into the process [43,44,45]. Despite attempts to address this limitation, the most commonly used acetylene surrogate, bis(trimethylsilyl)acetylene, has shown limited reactivity and stability, resulting in restricted product formation [46]. To overcome these obstacles, alternative acetylene surrogates have been explored, such as vinyl acetate [47,48,49] and alpha-halo acetaldehyde [22]. However, the outcomes of these reactions have been moderate in terms of product yields. In a more recent development, Miura and colleagues described a rhodium-catalyzed annulative coupling reaction employing vinylene carbonate as an acetylene equivalent (Scheme 1e) [24]. The process successfully synthesized unsubstituted 1,2-benzothiazines, achieving a yield of 30%. This strategy offers a promising resolution to the inherent difficulties encountered in acetylene-based reactions.

Vinylene carbonate serves as an efficient and practical acetylene surrogate, offering improved reactivity and stability. This advancement opens new possibilities for the synthesis of vinylene-fused compounds in a more controlled and efficient manner. Taking inspiration from previous discoveries in the field and drawing on our group’s extensive research on sulfoximine-assisted annulation and annulative functionalizations [50,51,52,53,54,55,56,57], we focused our attention on investigating the annulation of NH-sulfoximines with vinylene carbonate as a means to construct 1,2-benzothiazine derivatives. An intriguing aspect of vinylene carbonate is its dual role as both an acetylene surrogate and an oxidizing agent; it can be used for a redox-neutral process [24,58,59,60,61,62,63,64,65,66,67,68], which enhances the catalyst turnover and contributes to the overall efficiency of the reaction. 

## 2. Results and Discussion

In this article, we present the successful implementation of a Rh(III)-catalyzed redox-neutral [4+2]-C–H annulation, utilizing sulfoximines and vinylene carbonate (Scheme 1f). This strategy enables the formation of unsubstituted 1,2-benzothiazines with moderate to good yields. By utilizing vinylene carbonate as a versatile acetylene surrogate and incorporating Rh(III) catalysis, we demonstrate the practicality and effectiveness of this approach for the synthesis of complex sulfur-containing heterocycles. The developed methodology expands the synthetic toolbox for accessing 1,2-benzothiazine derivatives and holds promise for the construction of diverse sulfur-bearing scaffolds in future applications.

In this study, our objective was to investigate the potential use of sulfoximine (**1a**) and vinylene carbonate (**2**) as a model substrate to evaluate the effectiveness of a rhodium complex catalyst in combination with various additives, ligands, and solvents. To begin with, we employed a catalytic system consisting of 3.0 mol% [Cp*RhCl_2_]_2_, 20 mol% AgSbF_6_, and 20 mol% **L1** as a ligand, with trifluoroethanol (TFE) serving as the solvent. The reaction was conducted at a temperature of 80 °C. Under these conditions, we successfully obtained the desired product **3a** with a yield of 30%. This result is documented in Table 1, entry 1, which summarizes the experimental details and outcomes of our investigation. By employing the specified catalytic system, we were able to demonstrate the efficacy of the rhodium complex catalyst in promoting the desired transformation and generating the target product. However, it was observed that at 80 °C, the sulfoximine substrate underwent cleavage to form sulfoxide as an undesired byproduct. To overcome this issue and promote smoother reaction progress, the decision was made to lower the reaction temperature to 60 °C. With the identical catalytic conditions employed in entry 1, we proceeded to screen various solvents at the reduced temperature of 60 °C. This screening process, detailed in Table 1, entries 2 to 6, allowed us to identify the most effective solvent. Among the tested solvents, *t*-BuOH exhibited superior performance, providing the desired product **3a** with a yield of 55% (entry 6). Subsequently, our attention turned to the investigation of different ligands, as documented in Table 1, entries 7 to 9, using the catalytic system established in entry 6. Through this screening process, we aimed to identify a suitable ligand that would further enhance the yield of product **3a**. Ultimately, ligand **L4** proved to be the most suitable, delivering the desired compound with a yield of 75% (entry 9). These results highlight the significance of ligand selection in the context of the catalytic system, as it played a crucial role in promoting the formation of the target product and achieving higher yields. 

Our experimental findings provide compelling evidence for the crucial role of the ligand in facilitating the C–H activation process through the chelation-assisted C–H metalation–deprotonation (CMD) pathway. Notably, in the absence of a suitable ligand, as demonstrated in entry 10, the reaction failed to proceed, underscoring the indispensability of the ligand in promoting the desired transformation. In addition to exploring the impact of the ligand, we also aimed to investigate the influence of the catalyst on the reaction outcome. However, our attempts to employ 3 mol% of Co(III) and Ir(III) complexes, as documented in Table 1 (entries 11 and 12), were met with limited success, as the desired product was not obtained. Nevertheless, our exploration of alternative catalysts led us to evaluate the performance of [Ru(*p*-cymene)Cl_2_]_2_ at a catalyst loading of 3 mol%. Encouragingly, this catalyst enabled the formation of product **3a** with a yield of 55%, as shown in Table 1, entry 13. This result highlights the potential of this ruthenium-based catalyst in the present reaction system, albeit with a moderate yield. Furthermore, we investigated the reaction in the presence of AcOH (acetic acid) and pivalic acid under the catalytic conditions shown in entry 9 without a ligand. Although these acids had a significant impact on the CMD process [69,70,71], the reaction led to a diminished product yield of 23% and 39%, (see the supplementary material) respectively (entries 14 and 15). Although the exact reason for the reaction outcome in the presence of amino acid ligand L4 is unknown, we believe that the ligand bulkiness assists in promoting the reactivity. 

**Scheme 2:**
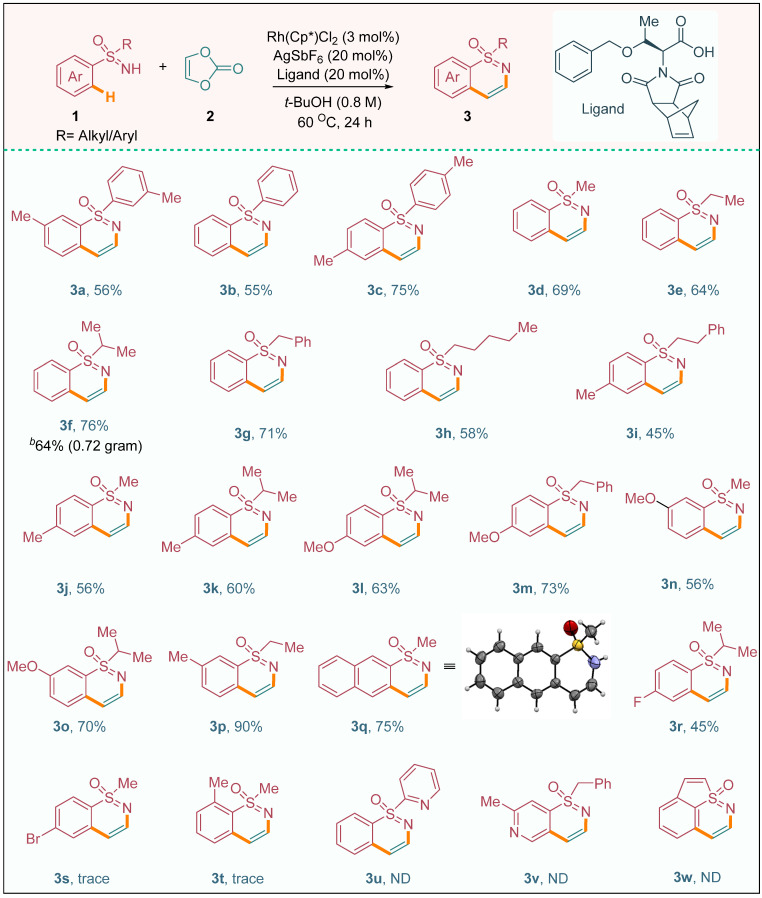
The substrate scope of sulfoximines ^a^. ^a^ Reaction conditions: **1a** (0.3 mmol), **2** (3.0 equiv), [Rh(Cp*)Cl_2_]_2_ (3.0 mol%), and AgSbF_6_ (20 mol%) in *t*–BuOH (2.4 mL) under nitrogen at 60 °C for 24 h. ^b^ Gram scale. ND = not determined.

These observations emphasize the significance of both the ligand and the catalyst in orchestrating the C–H activation process and achieving efficient conversion of the starting materials into the desired product. Following an extensive series of optimization endeavors, we proceeded to explore the applicability of various sulfoximine derivatives in the reaction system, as depicted in Scheme 2. Specifically, we investigated the reaction of di-aryl sulfoximines featuring donating groups (**1a** and **1c**) or no substitution (**1b**) with vinylene carbonate (**2**) to assess their potential as viable substrates. Gratifyingly, these reactions resulted in the formation of the desired products **3a**, **3b**, and **3c**, exhibiting moderate to good yields. The successful annulation of methyl phenyl sulfoximine (**1d**) with vinylene carbonate (**2**) proceeded smoothly, leading to the formation of the annulation product **3d** at a satisfactory yield of 69%. This result highlights the efficiency and effectiveness of our reaction system in transforming methyl phenyl sulfoximine into the desired 1,2-benzothiazine derivative.

In a similar fashion, phenyl alkyl/benzyl sulfoximines (**1e**–**i**) exhibited excellent reactivity and performance, affording the desired 1,2-benzothiazine derivatives **3e**–**i** with moderate to good yields. These findings indicate the broad substrate scope and versatility of our methodology, as it enables the construction of diverse 1,2-benzothiazine derivatives from various phenyl alkyl/benzyl sulfoximines. The reliable and consistent formation of the desired products further establishes the potential of sulfoximines as valuable building blocks for the synthesis of 1,2-benzothiazine derivatives. These findings not only expand the synthetic possibilities but also provide valuable insights into the reactivity patterns and synthetic utility of different sulfoximine derivatives in this annulation process. The successful implementation of our methodology extended to the annulation of aryl-alkylsulfoximines **1j**–**m**, which featured *para*-donating group substitutions on the aryl motifs and different alkyl/benzyl moieties. This transformation provided a convenient route for accessing the corresponding 1,2-benzothiazine derivatives **3j** (56% yield), **3k** (60% yield), **3l** (63% yield), and **3m** (73% yield). Notably, the annulation occurred selectively at the sterically less hindered C–H bond of the *meta*-substituted aryl moiety in the sulfoximine, resulting in the formation of products **3n** (56% yield) and **3o** (70% yield). Furthermore, we successfully synthesized the ethyl *meta*-tolyl sulfoximine-derived product **3p**, achieving an impressive yield of 90%. Additionally, the annulation of β-naphthyl methyl sulfoximine (**1q**) with **2** yielded the desired product **3q** in a high yield of 75%. The structures of the synthesized compounds were further confirmed by X-ray diffraction analysis (see the supplementary material), providing unequivocal evidence for the formation of product **3q**. These results highlight the robustness and versatility of our methodology, enabling the efficient synthesis of diverse 1,2-benzothiazine derivatives from a range of aryl-alkyl sulfoximines with different substituents. The high yields obtained in these transformations demonstrate the effectiveness of our approach and open up new possibilities for the synthesis of structurally diverse and pharmaceutically relevant 1,2-benzothiazine derivatives. In contrast to the successful annulation reactions observed with other sulfoximines, the presence of an electron-withdrawing group on the aryl moiety in sulfoximine 1**r** significantly affected the reactivity, resulting in a low yield of product **3r**. The electron-withdrawing nature of the substituent likely diminished the reactivity of the C–H bond, leading to a less favorable chelation-assisted C–H metalation–deprotonation (CMD) process and subsequently hindering the formation of the desired product. Regrettably, the aryl-sulfoximines bearing *para*-bromo substitution (**1s**) and *ortho*-methyl substitution (**1t**) did not participate in the annulation reaction. This lack of reactivity can be attributed to a combination of electronic and steric factors that impede the CMD process. The presence of a *para*-bromo substituent likely has a strong electron-withdrawing effect, further diminishing the reactivity of the C–H bond. Similarly, the *ortho*-methyl substitution introduces steric hindrance, making it difficult for the catalyst to access and activate the C–H bond effectively. Likewise, heterocyclic sulfoximines exert a significant influence on reactivity. In particular, the compounds 2-pyridyl phenyl sulfoximine (**3u**) and benzyl(imino)(2-methylpyridin-4-yl)-λ6-sulfanone (**3v**) are distinct; they do not participate in the annulation process due to the strong binding affinity of nitrogen to the metal catalyst. Moreover, the non-involvement of benzothiophene sulfoximine (**3w**) is attributed to the lack of planarity between the nitrogen atom of the sulfoximine and the arene C–H bonds, thereby exerting a discernible impact on reactivity. These findings, therefore, warrant further investigation.

Moreover, the observations emphasize the importance of the electronic and steric properties of the sulfoximine substrates in governing the success of the annulation reaction. It is intriguing to note that the incorporation of alkyl groups into the sulfoximine motifs, including methyl (**1d**), ethyl (**1e**), isopropyl (**1f**, **1k**, **1l**, and **1o**), and benzyl (**1g** and **1m**), resulted in highly efficient annulation reactions, and no cleavage of the starting materials was observed. These alkyl-substituted sulfoximines exhibited excellent reactivity and demonstrated remarkable effectiveness in yielding the desired products.

Moreover, the success of the annulation reaction was not limited to small-scale reactions but extended to gram-scale synthesis as well. For instance, NH-sulfoximine **1f** was subjected to the annulation process, and a substantial quantity of product **3f** (0.75 g) was obtained with an impressive yield of 64%. Overall, the introduction of alkyl groups on the sulfoximine moieties significantly enhanced the efficiency and applicability of the annulation reaction, enabling the synthesis of diverse compounds in satisfactory yields while maintaining the integrity of the starting materials. 

The incorporation of chiral sulfoximines plays a pivotal role in the molecular design of pharmaceutical compounds due to their significant presence and potential therapeutic applications. Consequently, the development of efficient synthetic methodologies for the construction of chiral sulfoximine derivatives has garnered considerable attention within the scientific community. The ability to access diverse chiral sulfoximine scaffolds through synthetic approaches holds great significance in drug discovery and medicinal chemistry. These chiral motifs can impart unique stereochemical properties to the target molecules, influencing their biological activity, selectivity, and pharmacokinetic profiles. Therefore, the exploration and advancement of synthetic methods to access chiral sulfoximines are of paramount importance for the development of novel pharmaceutical agents. The pursuit of efficient and versatile synthetic strategies for chiral sulfoximine synthesis not only addresses the growing demand for enantiopure compounds but also enables the exploration of structure–activity relationships, identification of new drug targets, and optimization of therapeutic properties. Consequently, the field of chiral sulfoximine synthesis continues to attract significant research interest and holds immense promise for the discovery and development of pharmaceutical compounds with enhanced efficacy and reduced side effects.

**Scheme 3:**
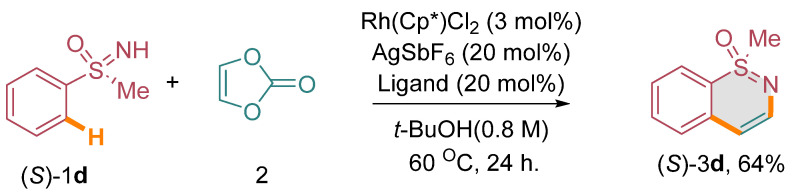
Annulation with enantiopure sulfoximines.

**Scheme 4:**
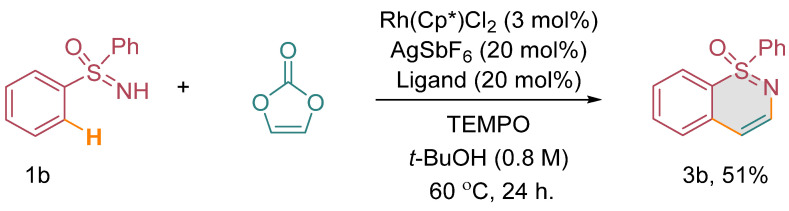
Radical quenching experiment.

Considering the limitations in accessing optically active 1,2-benzothiazine derivatives through conventional methods, we conceived the idea of investigating the annulation of enantiopure sulfoximines with vinylene carbonate under carefully optimized catalytic conditions. Our objective was to achieve the synthesis of these valuable compounds with preserved stereochemistry, which would otherwise be challenging to obtain using conventional synthetic approaches. To demonstrate the feasibility of this approach, we focused on the annulation of chiral methyl phenyl sulfoximine, specifically the (*S*)-enantiomer, designated as **(*S*)-1d**, with vinylene carbonate (**2**). Our expectation was to obtain the corresponding optically active 1,2-benzothiazine derivative, namely **(*S*)-3d [**(Scheme 3) (see the supplementary material)]. It was crucial for us to confirm that the chiral sulfoximine’s stereointegrity would be maintained throughout the annulation process. Our experimental results affirmed the successful conversion of **(*S*)-1d** to **(*S*)-3d** through the annulation reaction. Notably, the stereogenic centers of the chiral sulfoximine remained unchanged, indicating the retention of its stereochemical integrity during the annulation process. Furthermore, to investigate the involvement of radicals in the reaction, we performed a radical quenching experiment using diphenyl sulfoxime **1b**, vinylene carbonate, and TEMPO as a radical quencher under optimized catalytic conditions. Surprisingly, we obtained a 51% yield of the annulated product **3b**, indicating that the reaction was not quenched by radicals (Scheme 4).

**Scheme 5:**
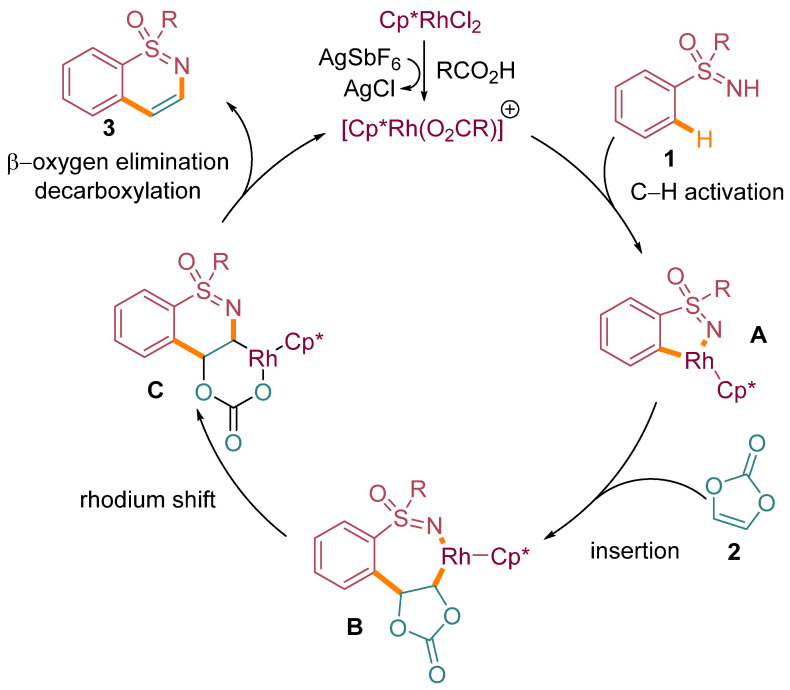
Proposed mechanism for the annulation of sulfoximines.

A plausible mechanistic pathway, supported by previous studies [24], has been proposed to elucidate the key steps involved in the annulation process. As illustrated in Scheme 5, the reaction initiation involves the formation of a five-membered rhodacycle intermediate **A**. This intermediate is formed through the coordination of the cationic rhodium catalyst with the nitrogen atom of the sulfoximine moiety in compound **1**. Importantly, this coordination is facilitated by the carboxylate-assisted metalation–deprotonation (CMD) process, which involves the activation of the *ortho*-C–H bond of the arene moiety in compound **1**. 

The N-protected amino acid ligand further enhances this *ortho*-C–H bond activation. Following the formation of intermediate **A**, migratory insertion occurs, where vinylene carbonate **2** reacts with the rhodium center, leading to the formation of a seven-membered rhodium complex, denoted as intermediate **B**. This step involves the migration of the rhodium atom from the five-membered ring to the carbonate moiety to give intermediate **C**. The overall process is redox-neutral, preserving the oxidation state of the rhodium catalyst. Notably, this step is followed by a β-oxygen elimination process, resulting in the closure of the catalytic cycle and the liberation of the final product. The reaction represented in Scheme 5 visualizes the sequential transformations and intermediates along this proposed mechanistic pathway.

## 3. Materials and Methods

### 3.1. General Information

All reagents and solvents were purchased from commercial sources, including BLD Pharmatech Ltd. headquartered in Shanghai, China., TCI Chemicals India Pvt Ltd., Turkapally, Hyderabad, India., Spectrochem Pvt. Ltd., Dabholkar Wadi, Mumbai, India., Avra Synthesis Pvt Ltd., Sai Enclave Habsiguda, Hyderabad, India., Jinay Pharmaceuticals Pvt Ltd., Thane, Mumbai, India., Sigma Aldrich Chemicals Pvt Ltd., Bangalore, India., and Finar Chemicals Pvt Ltd., Ahmedabad, Gujarat, India., and were used without further purification. Analytical thin-layer chromatography (TLC) was performed on HSGF 254 plates with a thickness of 0.15–0.2 mm. All products were characterized by their NMR and MS spectra. ^1^H and ^13^C nuclear magnetic resonance spectra (NMR) were acquired on a Bruker 400 MHz or 500 MHz NMR spectrometer, and chemical shifts were reported in parts per million (ppm) downfield from tetramethylsilane. The coupling constants (J) were indicated in Hz, and proton coupling patterns were described as singlet (s), doublet (d), triplet (t), quartet (q), multiplet (m), doublet of doublets (dd), and broad (br). High-resolution mass spectra (HRMS) data were measured on an Agilent G6520 Q-TOF instrument (Palo Alto, CA, USA) with electrospray ionization (ESI).

### 3.2. General Procedure for the Synthesis of Substrates **1a**–**1w**

A solution of sulfide (1.0 mmol) in MeOH (7.0 mL) was added to (NH_4_)_2_CO_3_ (1.2 mmol). Subsequently, PhI(OAc)_2_ (2.2 mmol) was slowly added, and the solution was stirred at room temperature. After complete consumption of the starting material, the solvents were removed under reduced pressure, and the crude product was purified by flash column chromatography using an eluent of EtOAc/hexane from 10:1 to 1:1 to yield the desired product **1**.

### 3.3. General Procedure for the Synthesis of Compounds **3a**–**3w**

Sulfoximine **1** (0.3 mmol), vinylene carbonate (0.6 mmol), [Cp*RhCl_2_]_2_ (3.0 mol%; 0.009 mmol), and AgSbF_6_ (20 mol%; 0.06 mmol) were added to a 15 mL screw-capped vial under nitrogen. Tertiary butanol was added under nitrogen, and the resulting mixture was stirred at 60 °C for 24 h. Upon completion of the reaction, the solvents were evaporated under reduced pressure, and the residue was purified by silica gel chromatography using an eluent of EtOAc/hexane from 18:1 to 10:3 to yield the desired product **3**.

### 3.4. General Procedure for the Synthesis of Ligands L1, L2, and L4

To prepare the title compound, a mixture of a-amino acid (1.0 equiv.) and its corresponding anhydride (1.0 equiv.) was heated at 120 °C for 4 h. After cooling to room temperature, 50 mL of CHCl_3_ and 50 mL of aqueous 1 N HCl were added to the reaction mixture. The aqueous layer was extracted with CHCl_3_, and the combined organic layer was washed with brine and dried over Na_2_SO_4_. The solvent was then removed under reduced pressure to yield the title compound.

### 3.5. Characterization of the Products

7-Methyl-1-(*m*-tolyl)benzo[*e*][1,2]thiazine 1-oxide **(3a)**: Yellow gummy liquid (45 mg, 56% yield); ^1^H NMR (500 MHz, CDCl_3_) δ 7.82–7.75 (m, 1H), 7.75–7.70 (m, 1H), 7.52–7.42 (m, 2H), 7.33 (dd, *J* = 8.2, 1.7 Hz, 1H), 7.28 (d, *J* = 8.2 Hz, 1H), 7.24 (d, *J* = 6.9 Hz, 1H), 7.12–7.08 (m, 1H), 6.21 (dd, *J* = 6.9, 0.8 Hz, 1H), 2.45 (s, 3H), 2.30 (s, 3H). ^13^C NMR (101 MHz, CDCl_3_) δ 139.52, 139.14, 137.00, 136.15, 134.64, 133.97, 133.29, 129.57, 128.93, 126.46, 126.23, 124.35, 120.42, 101.74, 21.33, 21.25. HRMS (ESI): Calculated for C16H16NOS [M + H]^+^: 270.0948, found: 270.0950.

1-Phenylbenzo[*e*][1,2]thiazine 1-oxide **(3b)**: Yellow gummy liquid (40 mg, 55% yield); ^1^H NMR (500 MHz, CDCl_3_) δ 7.96–7.92 (m, 2H), 7.70–7.63 (m, 1H), 7.62–7.56 (m, 2H), 7.52–7.48 (m, 1H), 7.36 (dd, *J* = 8.1, 1.2 Hz, 1H), 7.33–7.29 (m, 2H), 7.28–7.25 (m, 1H), 6.24 (dd, *J* = 6.9, 0.8 Hz, 1H). ^13^C NMR (126 MHz, CDCl_3_) δ 140.24, 138.53, 135.90, 133.49, 132.12, 129.13, 128.99, 126.47, 126.14, 125.13, 120.22, 101.52. HRMS (ESI): Calculated for C14H12NOS [M + H]^+^: 242.0635, found: 242.0639.

6-Methyl-1-(*p*-tolyl)benzo[*e*][1,2]thiazine 1-oxide **(3c)**: Yellow solid (70 mg, 75% yield); m.p.: 140–141 °C; ^1^H NMR (500 MHz, CDCl_3_) δ 7.79 (d, *J* = 8.4 Hz, 2H), 7.38–7.33 (dd, *J* = 8.5, 0.8 Hz, 2H), 7.28–7.23 (m, 1H), 7.20 (d, *J* = 8.3 Hz, 1H), 7.14–7.11 (t, *J* = 1.0 Hz 1H), 7.05 (dd, *J* = 8.2, 1.7 Hz, 1H), 6.13 (dd, *J* = 6.9, 0.8 Hz, 1H), 2.44 (s, 3H), 2.38 (s, 3H). ^13^C NMR (126 MHz, CDCl_3_) δ 144.60, 142.82, 137.96, 137.07, 135.82, 129.64, 129.05, 127.88, 125.81, 125.03, 118.23, 101.39, 21.67, 21.55. HRMS (ESI): Calculated for C16H16NOS [M + H]^+^: 270.0948, found: 270.0948.

1-Methyl-1λ^4^-benzo[*e*][1,2]thiazine 1-oxide **(3d)**: Yellow solid (38 mg, 69% yield); m.p.: 134–135 °C; ^1^H NMR (500 MHz, CDCl_3_) δ 7.77 (d, *J* = 8.0 Hz, 1H), 7.58–7.53 (m, 1H), 7.40 (td, *J* = 7.7, 1.2 Hz, 1H), 7.31 (dd, *J* = 8.0, 1.2 Hz, 1H), 7.06 (d, *J* = 6.9 Hz, 1H), 6.07 (dd, *J* = 6.8, 0.8 Hz 1H), 3.56 (s, 3H). ^13^C NMR (126 MHz, CDCl_3_) δ 137.87, 135.84, 132.77, 126.62, 126.45, 123.58, 119.39, 101.63, 45.33. HRMS (ESI): Calculated for C9H10NOS [M + H]^+^: 180.0478, found: 180.0480. 

1-Ethyl-1λ^4^-benzo[*e*][1,2]thiazine 1-oxide **(3e)**: Yellow gummy liquid (37 mg, 64% yield); ^1^H NMR (500 MHz, CDCl_3_) δ 7.72 (d, *J* = 8.1 Hz, 1H), 7.58–7.54 (m, 1H), 7.41–7.37 (m, 1H), 7.32 (dd, *J* = 8.0, 1.2 Hz, 1H), 7.12 (d, *J* = 6.9 Hz, 1H), 6.01 (dd, *J* = 7.0, 0.8 Hz, 1H), 3.81–3.60 (m, 2H), 1.23 (t, *J* = 7.3 Hz, 3H). ^13^C NMR (101 MHz, CDCl_3_) δ 137.79, 137.02, 133.12, 126.70, 126.64, 124.04, 116.52, 101.15, 51.30, 8.58. HRMS (ESI): Calculated for C10H11NOSNa [M + Na]^+^: 216.0454, found: 216.0454.

1-Isopropyl-1λ^4^-benzo[*e*][1,2]thiazine 1-oxide **(3f)**: Yellow gummy liquid (47 mg, 76% yield); ^1^H NMR (500 MHz, CDCl_3_) δ 7.75–7.66 (m, 1H), 7.56–7.52 (m, 1H), 7.38–7.33 (m, 1H), 7.27 (d, *J* = 5.6 Hz, 1H), 7.10 (d, *J* = 6.9 Hz, 1H), 5.92 (dd, *J* = 6.9, 0.8 Hz, 1H), 3.88–3.82 (m, 1H), 1.50 (d, *J* = 6.9 Hz, 3H), 1.18 (d, *J* = 6.7 Hz, 3H). ^13^C NMR (126 MHz, CDCl_3_) δ 138.90, 137.79, 133.01, 126.34, 126.23, 124.52, 115.57, 100.45, 58.23, 17.16, 13.52. HRMS (ESI): Calculated for C11H14NOS [M + H]^+^: 208.0791, found: 208.0790.

1-Benzyl-1λ^4^-benzo[*e*][1,2]thiazine 1-oxide **(3g)**: Yellow solid (55 mg, 71% yield); m.p.: 166–167 °C; ^1^H NMR (500 MHz, CDCl_3_) δ 7.60 (d, *J* = 8.0 Hz, 1H), 7.51–7.46 (m, 1H), 7.33–7.27 (m, 2H), 7.24–7.20 (m, 2H), 7.17 (dt, *J* = 6.9, 1.5 Hz, 2H), 7.14 (dd, *J* = 8.1, 1.1 Hz, 1H), 6.98 (d, *J* = 6.9 Hz, 1H), 5.75 (d, *J* = 6.9 Hz, 1H), 4.83–4.62 (m, 2H). ^13^C NMR (101 MHz, CDCl_3_) δ 139.53, 137.95, 133.05, 131.12, 128.76, 128.70, 128.54, 128.42, 127.82, 126.00, 125.87, 125.25, 116.23, 100.46, 64.66. HRMS (ESI): Calculated for C15H14NOS [M + H]^+^: 256.0791, found: 256.0796.

1-Pentyl-1λ^4^-benzo[*e*][1,2]thiazine 1-oxide **(3h)**: Yellow gummy liquid (41 mg, 58% yield); ^1^H NMR (500 MHz, CDCl_3_) δ 7.72 (dd, *J* = 8.0, 1.2 Hz, 1H), 7.58–7.54 (m, 1H), 7.42–7.38 (m, 1H), 7.34–7.30 (m, 1H), 7.11 (d, *J* = 6.9 Hz, 1H), 6.02 (dd, *J* = 7.0, 0.8 Hz, 1H), 3.78–3.57 (m, 2H), 1.82–1.43 (m, 2H), 1.42–1.17 (m, 4H), 0.84 (t, *J* = 7.2 Hz, 3H). ^13^C NMR (126 MHz, CDCl_3_) δ 137.53, 136.69, 133.06, 126.67, 126.62, 123.99, 117.28, 101.19, 56.66, 29.99, 23.52, 22.02, 13.65. HRMS (ESI): Calculated for C13H18NOS [M + H]^+^: 236.1104, found: 236.1103.

6-Methyl-1-phenethyl-1λ^4^-benzo[*e*][1,2]thiazine 1-oxide **(3i)**: Yellow gummy liquid (39 mg, 45% yield); ^1^H NMR (500 MHz, CDCl_3_) δ 7.58 (d, *J* = 8.2 Hz, 1H), 7.27–7.16 (m, 4H), 7.16–7.09 (m, 4H), 6.02 (d, *J* = 7.0 Hz, 1H), 4.07–3.95 (m, 2H), 3.13–2.76 (m, 2H), 2.44 (s, 3H). ^13^C NMR (101 MHz, CDCl_3_) δ 144.49, 136.66, 136.33, 128.82, 128.42, 126.99, 126.62, 124.10, 114.94, 101.69, 57.84, 30.12, 21.80. HRMS (ESI): Calculated for C17H18NOS [M + H]^+^: 284.1104, found: 284.1108.

1,6-Dimethyl-1λ^4^-benzo[*e*][1,2]thiazine 1-oxide **(3j)**: Yellow gummy liquid (32 mg, 56% yield); ^1^H NMR (500 MHz, CDCl_3_) δ 7.68 (d, *J* = 8.2 Hz, 1H), 7.25–7.22 (m, 1H), 7.11 (s, 1H), 7.03 (d, *J* = 6.9 Hz, 1H), 6.01 (dd, *J* = 7.0, 0.8 Hz, 1H), 3.57 (s, 3H), 2.43 (s, 3H). ^13^C NMR (126 MHz, CDCl_3_) δ 143.92, 136.95, 135.80, 128.20, 128.19, 126.36, 126.34, 123.66, 117.21, 101.78, 45.38, 45.37, 21.75. HRMS (ESI): Calculated for C10H12NOS [M + H]^+^: 194.0635, found: 194.0632.

1-Isopropyl-6-methyl-1λ^4^-benzo[*e*][1,2]thiazine 1-oxide **(3k)**: Yellow gummy liquid (40 mg, 60% yield); ^1^H NMR (500 MHz, CDCl_3_) δ 7.61 (d, *J* = 8.2 Hz, 1H), 7.19 (dd, *J* = 8.2, 1.6 Hz, 1H), 7.08 (d, *J* = 6.9 Hz, 2H), 5.86 (dd, *J* = 6.9, 0.8 Hz, 1H), 3.85 (p, *J* = 6.8 Hz, 1H) 3.88–3.82 (m, 1H), 2.41 (s, 3H), 1.49 (d, *J* = 6.9 Hz, 3H), 1.20 (d, *J* = 6.7 Hz, 3H). ^13^C NMR (126 MHz, CDCl_3_) δ 144.01, 138.50, 137.88, 127.82, 126.11, 124.62, 113.14, 100.45, 58.62, 21.74, 17.10, 13.71. HRMS (ESI): Calculated for C12H16NOS [M + H]^+^: 222.0948, found: 222.0950.

1-Isopropyl-6-methoxy-1λ^4^-benzo[*e*][1,2]thiazine 1-oxide **(3l)**: Yellow gummy liquid (45 mg, 63% yield); ^1^H NMR (500 MHz, CDCl_3_) δ 7.66–7.59 (dd, *J* = 9.0, 0.5 Hz, 1H), 7.08 (d, *J* = 6.9 Hz, 1H), 6.92 (dd, *J* = 8.9, 2.5 Hz, 1H), 6.64 (d, *J* = 2.5 Hz, 1H), 5.84 (dd, *J* = 6.9, 0.7 Hz, 1H), 3.87 (s, 3H), 3.82–3.75 (m, *J* = 6.8 Hz, 1H), 1.46 (d, *J* = 6.9 Hz, 3H), 1.19 (d, *J* = 6.8 Hz, 3H). ^13^C NMR (126 MHz, CDCl_3_) δ 163.05, 140.32, 139.46, 139.41, 126.81, 115.59, 108.11, 107.41, 100.43, 100.41, 59.06, 55.53, 16.97, 13.76. HRMS (ESI): Calculated for C12H15NO2SNa [M + Na]^+^: 260.0716, found: 260.0717.

1-Benzyl-6-methoxy-1λ^4^-benzo[*e*][1,2]thiazine 1-oxide **(3m)**: Yellow gummy liquid (63 mg, 73% yield); ^1^H NMR (500 MHz, CDCl_3_) δ 7.46 (dd, *J* = 8.9, 0.7 Hz, 1H), 7.29–7.25 (m, 1H), 7.24–7.20 (m, 2H), 7.21–7.11 (m, 2H), 6.95 (d, *J* = 6.9 Hz, 1H), 6.84 (dd, *J* = 8.9, 2.5 Hz, 1H), 6.49 (d, *J* = 2.5 Hz, 1H), 5.64 (dd, *J* = 7.0, 0.8 Hz, 1H), 4.72–4.54 (m, 2H), 3.83 (s, 3H). ^13^C NMR (126 MHz, CDCl_3_) δ 162.92, 140.42, 140.26, 131.08, 128.67, 128.37, 128.05, 127.38, 115.10, 108.98, 106.93, 100.30, 65.08, 55.41. HRMS (ESI): Calculated for C16H16NO2S [M + H]^+^: 286.0897, found: 286.0896.

7-Methoxy-1-methyl-1λ^4^-benzo[*e*][1,2]thiazine 1-oxide **(3n)**: Yellow solid (35 mg, 56% yield); m.p.: 141–142 °C; ^1^H NMR (500 MHz, CDCl_3_) δ 7.28–7.25 (m, 1H), 7.23–7.15 (m, 2H), 6.93 (dd, *J* = 7.0, 2.0 Hz, 1H), 6.06–6.00 (m, 1H), 3.88 (d, *J* = 0.9 Hz, 3H), 3.61 (d, *J* = 1.9 Hz, 3H). ^13^C NMR (126 MHz, CDCl_3_) δ 158.36, 134.46, 129.35, 128.29, 122.29, 119.89, 105.28, 101.68, 55.82, 45.12. HRMS (ESI): Calculated for C10H11NO2SNa [M + Na]^+^: 232.0403, found: 232.0403.

1-Isopropyl-7-methoxy-1λ^4^-benzo[*e*][1,2]thiazine 1-oxide **(3o)**: Yellow gummy liquid (50 mg, 70% yield); ^1^H NMR (500 MHz, CDCl_3_) δ 7.22 (d, *J* = 8.7 Hz, 1H), 7.17 (dd, *J* = 8.8, 2.6 Hz, 1H), 7.13 (d, *J* = 2.5 Hz, 1H), 6.99 (d, *J* = 6.9 Hz, 1H), 5.88 (dd, *J* = 6.9, 0.8 Hz, 1H), 3.91–3.87 (m, 1H), 3.85 (s, 3H), 1.50 (d, *J* = 6.9 Hz, 3H), 1.19 (d, *J* = 6.7 Hz, 3H). ^13^C NMR (126 MHz, CDCl_3_) δ 158.08, 135.99, 131.61, 127.99, 122.41, 115.94, 106.15, 100.29, 58.22, 55.75, 17.27, 13.55. HRMS (ESI): Calculated for C12H16NO2S [M + H]^+^: 238.0897, found: 238.0894.

1-Ethyl-7-methyl-1λ^4^-benzo[*e*][1,2]thiazine 1-oxide **(3p)**: Yellow gummy liquid (56 mg, 90% yield); ^1^H NMR (500 MHz, CDCl_3_) δ 7.49 (s, 1H), 7.38 (dd, *J* = 8.3, 1.8 Hz, 1H), 7.21 (d, *J* = 8.1 Hz, 1H), 7.06 (d, *J* = 6.9 Hz, 1H), 5.96 (d, *J* = 6.9 Hz, 1H), 3.97–3.48 (m, 2H), 2.42 (s, 3H), 1.20 (t, *J* = 7.3 Hz, 3H). ^13^C NMR (126 MHz, CDCl_3_) δ 137.35, 136.83, 134.77, 134.43, 126.45, 123.33, 116.29, 100.73, 51.31, 21.27, 8.54. HRMS (ESI): Calculated for C11H14NOS [M + H]^+^: 208.0791, found: 208.0794

1-Methyl-1λ^4^-naphtho [2,3-*e*][1,2]thiazine 1-oxide **(3q)**: Yellow crystalline solid (51 mg, 75% yield); m.p.: 172–173 °C; ^1^H NMR (500 MHz, CDCl_3_) δ 8.43 (s, 1H), 7.94 (dd, *J* = 8.4, 1.1 Hz, 1H), 7.87 (d, *J* = 8.4 Hz, 1H), 7.75 (s, 1H), 7.62–7.58 (m, 1H), 7.52–7.48 (m, 1H), 6.96 (d, *J* = 7.1 Hz, 1H), 6.22 (d, *J* = 7.1 Hz, 1H), 3.62 (s, 3H). ^13^C NMR (101 MHz, CDCl_3_) δ 135.94, 135.64, 131.71, 131.16, 129.13, 128.90, 127.84, 126.23, 125.23, 123.91, 121.87, 102.36, 45.35. HRMS (ESI): Calculated for C13H12NOS [M + H]^+^: 230.0635, found: 230.0637

6-Fluoro-1-isopropyl-1λ^4^-benzo[e][1,2]thiazine 1-oxide **(3r)**: Yellow gummy liquid (30 mg, 45% yield); ^1^H NMR (500 MHz, CDCl_3_) δ 7.77–7.67 (m, 1H), 7.13 (dd, *J* = 6.9, 0.8 Hz, 1H), 7.06 (ddd, *J* = 8.8, 8.1, 2.5 Hz, 1H), 6.91 (dd, *J* = 9.8, 2.5 Hz, 1H), 5.86 (dd, *J* = 6.9, 0.8 Hz, 1H), 3.8–3.74 (m, 1H), 1.48 (d, *J* = 6.8 Hz, 3H), 1.18 (d, *J* = 6.7 Hz, 3H). ^19^F NMR (471 MHz, CDCl_3_) δ -104.43. ^13^C NMR (101 MHz, CDCl_3_) δ 141.74, 127.95, 127.85, 114.70, 114.46, 111.11, 110.89, 99.91, 59.05, 16.92, 13.68. HRMS (ESI): Calculated for C11H13FNOS [M + H]^+^: 226.0697, found: 226.0696.

## 4. Conclusions

In summary, we have presented a demonstration of the Rhodium(III)-catalyzed, redox-neutral [4+2]-C–H annulation of sulfoximine with the application of vinylene carbonate as an acetylene equivalent. Through this innovative approach, it is possible to achieve the direct synthesis of unsubstituted aryl-fused sulfoximine heterocycles. The reaction exhibits a broad scope, making a wide array of 1,2-benzothiazine derivatives in moderate to good yields.

## Figures and Tables

**Table 1:** Optimization of reaction conditions ^a^.

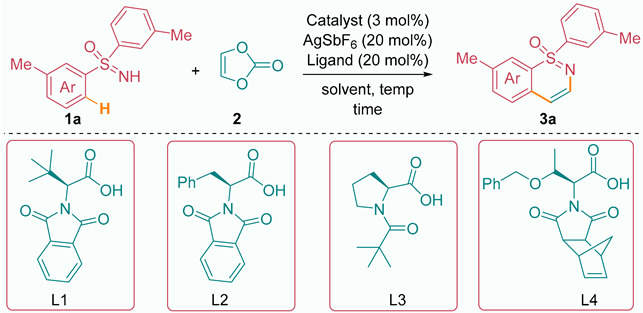					
Entry	Catalyst (3 mol%)	Ligand (20 mol%)	Solvent	Temp (°C)	Yield (%) ^b^
1	[Rh(Cp*)Cl_2_]_2_	L1	TFE	80	30
2	[Rh(Cp*)Cl_2_]_2_	L1	DCE	60	20
3	[Rh(Cp*)Cl_2_]_2_	L1	*t*–amyl–OH	60	50
4	[Rh(Cp*)Cl_2_]_2_	L1	TFE+DCE	60	15
5	[Rh(Cp*)Cl_2_]_2_	L1	TFT	60	35
6	[Rh(Cp*)Cl_2_]_2_	L1	*t*–BuOH	60	55
7	[Rh(Cp*)Cl_2_]_2_	L2	*t*–BuOH	60	50
8	[Rh(Cp*)Cl_2_]_2_	L3	*t*–BuOH	60	43
**9**	[Rh(Cp*)Cl_2_]_2_	**L4**	***t*** **–BuOH**	**60**	**75**
10	[Rh(Cp*)Cl_2_]_2_	No ligand	*t*–BuOH	60	Trace
11	[Ir(Cp*)Cl_2_]_2_	L4	*t*–BuOH	60	n.d
12	[Co(Cp*)(CO)I_2_]_2_	L4	*t*–BuOH	60	n.d
13	[Ru(*p*–cymene)Cl_2_]_2_	L4	*t*–BuOH	60	55
14	[Rh(Cp*)Cl_2_]_2_	AcOH	*t*–BuOH	60	23 ^c^
15	[Rh(Cp*)Cl_2_]_2_	Piv-OH	*t*–BuOH	60	39 ^c^

^a^ Reaction conditions: **1a** (0.3 mmol), **2** (3.0 equiv), catalyst and additive in solvent (2.4 mL) under nitrogen. ^b^ Isolated yield. ^c^ Yields are Determined by ^1^HNMR spectra 1,3,5-trymethoxy benzene as a reference. Entry 9 is the optimized condition.

## Data Availability

Not applicable.

## Supplementary Materials

The following supporting information can be downloaded at https://www.mdpi.com/article/10.3390/molecules28135014/s1. The optimization details and the spectra of the representative compounds.

## Sample Availability

Samples of the compounds are available from the authors.
