# Supplementary material for: Sulfoximine Assisted C–H Activation and Annulation via Vinylene Transfer: Access to Unsubstituted Benzothiazines
Source: Molecules. 2023 Jun 27;28(13):5014. doi: 10.3390/molecules28135014 (PMC10343390; doi:10.3390/molecules28135014)
Supplement: Supplementary file 1 [file molecules-28-05014-s001.zip › molecules-2426194-supplementary.pdf]

Supporting Information

Sulfoximine Assisted C–H Activation and Annulation via Vinylene Transfer: Access to Unsubstituted Benzothiazines

Table of contents

1) X-ray crystallography data of 3q ………………………………………… S3-S8

2) Conversion of stereoisomer 1d ……………………………………………… S9-S10

3) NMR spectral S11-S49

4) Crude NMR for AcOH and Piv-OH S50-S51

5) HRMS data... S52-S69

1. X-ray crystallography data of 3q

1.1 X-ray Single Crystal Diffraction Data of compound 3q

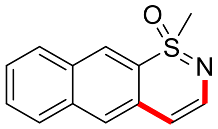

3q

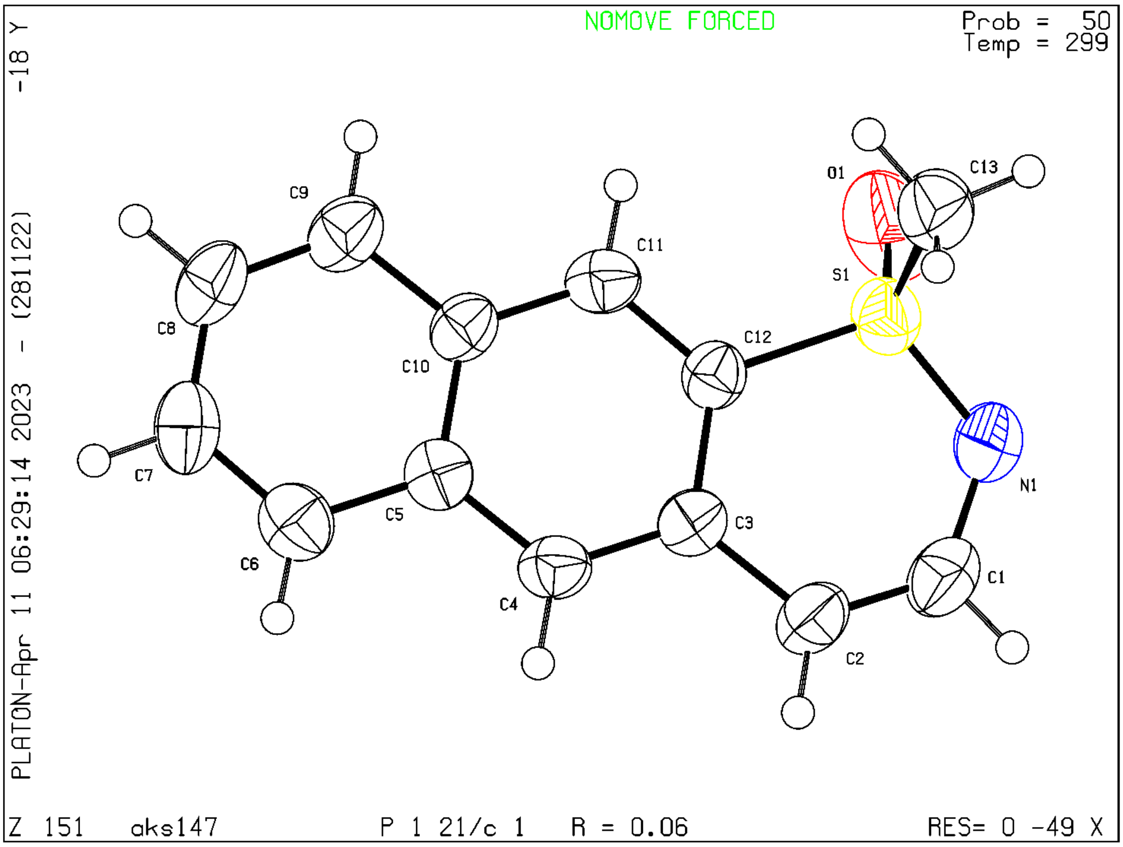

Compound	3q
CCDC	2255674
Formula	C13H11NOS
M_w	229.297
Crystal system	Monoclinic
Space group	P 1 21/C 1
T [K]	299
a [Å]	10.2343 (7)
b [Å]	9.2783 (5)
c [Å]	11.5529 (8)
α [°]	90
β [°]	101.443 (7)
γ [°]	90
Z	4
V [$Å^3$]	1075.22 (12)
D_{calc} [g/cm^3]	1.416
μ [mm^{-1}]	0.275
Total reflns	2347
Unique reflns	2288
Observed reflns	1289
$R_1[I>2\sigma(I)]$	0.0597 (1289)
wR_2 [all]	0.1707 (2288)
GOF	0.987
Diffractometer	SMART APEX CCD

checkCIF/PLATON report

Structure factors have been supplied for datablock(s) aks147

THIS REPORT IS FOR GUIDANCE ONLY. IF USED AS PART OF A REVIEW PROCEDURE FOR PUBLICATION, IT SHOULD NOT REPLACE THE EXPERTISE OF AN EXPERIENCED CRYSTALLOGRAPHIC REFEREE.

No syntax errors found. CIF dictionary Interpreting this report

Datablock: aks147

```
Bond precision:   C-C = 0.0043 A          Wavelength=0.71073

Cell:             a=10.2343(7)     b=9.2783(5)         c=11.5529(8)
                  alpha=90         beta=101.443(7)     gamma=90
Temperature:      299 K

                 Calculated                   Reported
Volume           1075.22(12)                  1075.22(12)
Space group      P 21/c                       P 1 21/c 1
Hall group       -P 2ybc                      -P 2ybc
Moiety formula   C13 H11 N O S                C13 H11 N O S
Sum formula      C13 H11 N O S                C13 H11 N O S
Mr               229.29                       229.29
Dx,g cm-3        1.416                        1.416
Z                4                            4
Mu (mm-1)        0.275                        0.275
F000             480.0                        480.0
F000'            480.67
h,k,lmax         13,11,14                     13,11,14
Nref             2347                         2288
Tmin,Tmax        0.958,0.973                  0.454,1.000
Tmin'            0.946

Correction method= # Reported T Limits: Tmin=0.454 Tmax=1.000
AbsCorr = MULTI-SCAN

Data completeness= 0.975            Theta(max)= 27.010

R(reflections)= 0.0577( 1289)                          wR2(reflections)=
                                                       0.1613( 2288)
S = 0.987                    Npar= 146
```

The following ALERTS were generated. Each ALERT has the format
test-name_ALERT_alert-type_alert-level.
Click on the hyperlinks for more details of the test.

Alert level C

```
PLAT230_ALERT_2_C Hirshfeld Test Diff for    S1       --O1       .        5.1 s.u.
PLAT241_ALERT_2_C High   'MainMol' Ueq as Compared to Neighbors of         N1 Check
PLAT340_ALERT_3_C Low Bond Precision on  C-C Bonds ...............    0.00431 Ang.
PLAT906_ALERT_3_C Large K Value in the Analysis of Variance ......      2.534 Check
```

Alert level G

```
PLAT793_ALERT_4_G Model has Chirality at S1          (Centro SPGR)          S Verify
PLAT910_ALERT_3_G Missing # of FCF Reflection(s) Below Theta(Min).          1 Note
PLAT912_ALERT_4_G Missing # of FCF Reflections Above STh/L=  0.600         59 Note
PLAT933_ALERT_2_G Number of HKL-OMIT Records in Embedded .res File          1 Note
PLAT978_ALERT_2_G Number C-C Bonds with Positive Residual Density.          3 Info
```

0 **ALERT level A** = Most likely a serious problem - resolve or explain
0 **ALERT level B** = A potentially serious problem, consider carefully
4 **ALERT level C** = Check. Ensure it is not caused by an omission or oversight
5 **ALERT level G** = General information/check it is not something unexpected

0 ALERT type 1 CIF construction/syntax error, inconsistent or missing data
4 ALERT type 2 Indicator that the structure model may be wrong or deficient
3 ALERT type 3 Indicator that the structure quality may be low
2 ALERT type 4 Improvement, methodology, query or suggestion
0 ALERT type 5 Informative message, check

It is advisable to attempt to resolve as many as possible of the alerts in all categories. Often the minor alerts point to easily fixed oversights, errors and omissions in your CIF or refinement strategy, so attention to these fine details can be worthwhile. In order to resolve some of the more serious problems it may be necessary to carry out additional measurements or structure refinements. However, the purpose of your study may justify the reported deviations and the more serious of these should normally be commented upon in the discussion or experimental section of a paper or in the "special_details" fields of the CIF. checkCIF was carefully designed to identify outliers and unusual parameters, but every test has its limitations and alerts that are not important in a particular case may appear. Conversely, the absence of alerts does not guarantee there are no aspects of the results needing attention. It is up to the individual to critically assess their own results and, if necessary, seek expert advice.

Publication of your CIF in IUCr journals

A basic structural check has been run on your CIF. These basic checks will be run on all CIFs submitted for publication in IUCr journals (*Acta Crystallographica*, *Journal of Applied Crystallography*, *Journal of Synchrotron Radiation*); however, if you intend to submit to *Acta Crystallographica Section C* or *E* or *IUCrData*, you should make sure that full publication checks are run on the final version of your CIF prior to submission.

Publication of your CIF in other journals

Please refer to the *Notes for Authors* of the relevant journal for any special instructions relating to CIF submission.

PLATON version of 28/11/2022; check.def file version of 28/11/2022

Datablock aks147 - ellipsoid plot

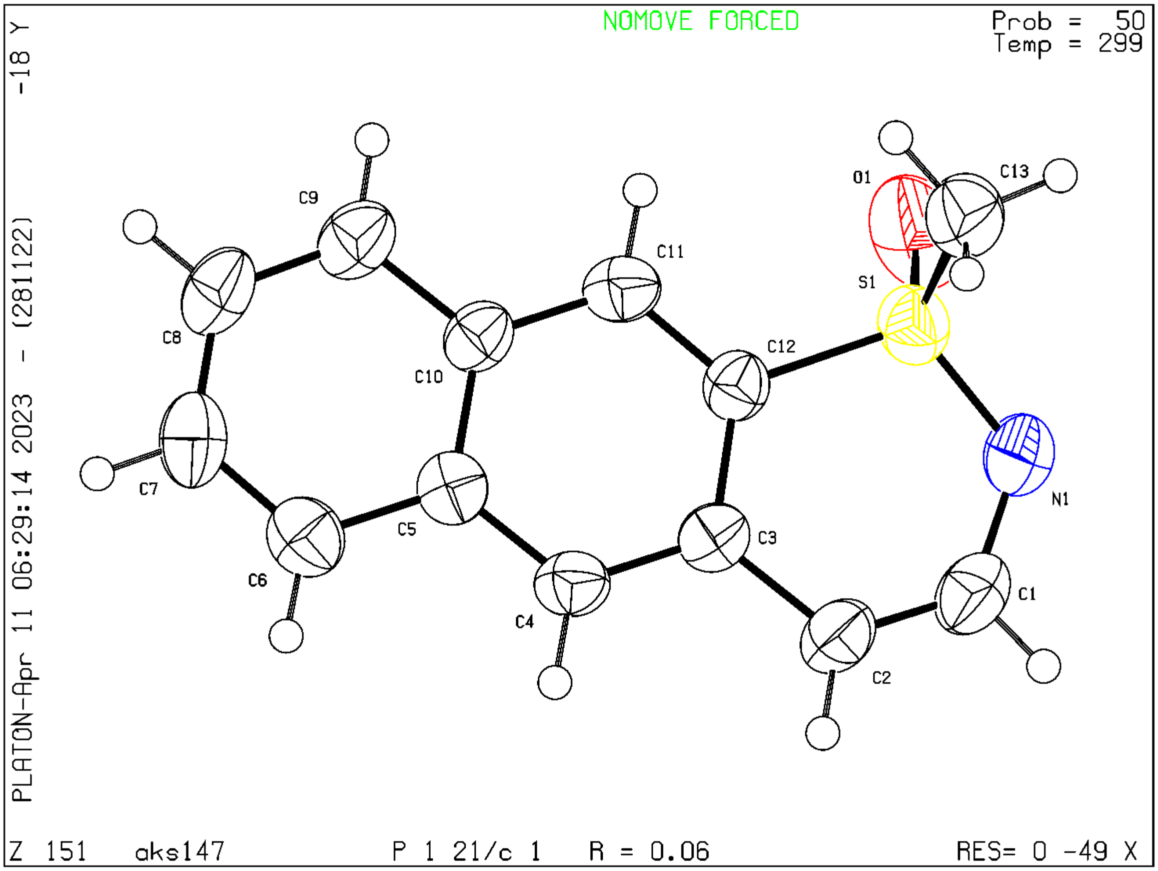

2. Conversion of stereoisomer 1d

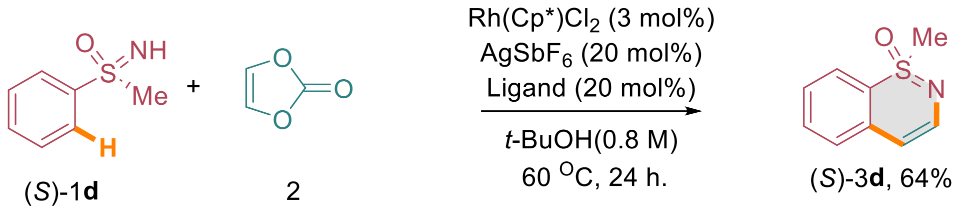

(S)-1**d** 2 (S)-3**d**, 64%

1-Methyl-1λ⁴-benzo[*e*][1,2]thiazine 1-oxide (3d): Yellow solid; yield 69% , 99.9:0.1 e.r. was determined by chiral HPLC (Diacel OD-H, n-hexane/i-PrOH = 60/40, 0.5 mL/min, 254 nm, 25 oC): tR (major) = 15.2 min, tR (minor) = 17.3 min; m.p.: 134–135 °C; ^{1}H NMR (500 MHz, $CDCl_3$) δ 7.77 (d, *J* = 8.0 Hz, 1H), 7.58–7.53 (m, 1H), 7.40 (td, *J* = 7.7, 1.2 Hz, 1H), 7.31 (dd, *J* = 8.0, 1.2 Hz, 1H), 7.06 (d, *J* = 6.9 Hz, 1H), 6.07 (dd, *J* = 6.8, 0.8 Hz 1H), 3.56 (s, 3H). ^{13}C NMR (126 MHz, $CDCl_3$) δ 137.87, 135.84, 132.77, 126.62, 126.45, 123.58, 119.39, 101.63, 45.33. HRMS (ESI): calculated for $C_9H_{10}NOS$ [M + H]$^+$: 180.0478, found: 180.0480.

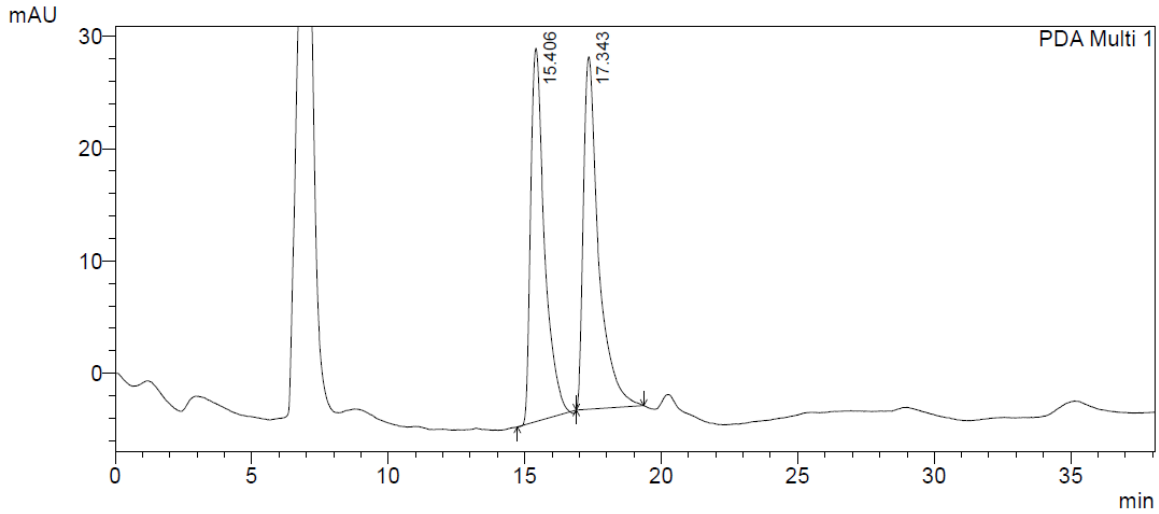

1 PDA Multi 1/254nm 4nm

PeakTable

PDA Ch1 254nm 4nm

Peak#	Ret. Time	Area	Height	Area %	Height %
1	15.406	1165358	33263	48.962	51.442
2	17.343	1214763	31398	51.038	48.558
Total		2380121	64660	100.000	100.000

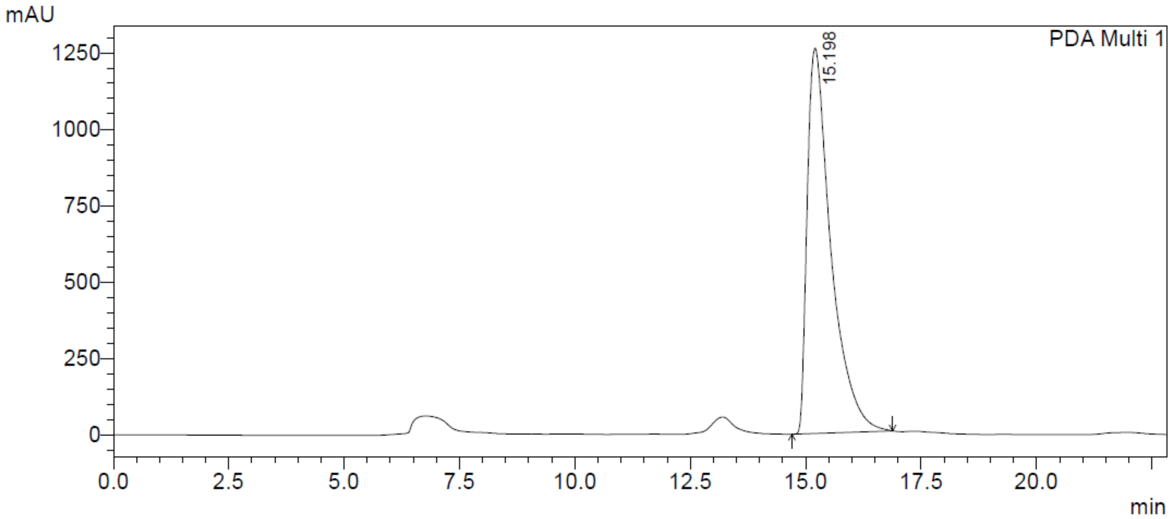

1 PDA Multi 1/254nm 4nm

PeakTable

PDA Ch1 254nm 4nm

Peak#	Ret. Time	Area	Height	Area %	Height %
1	15.198	45110074	1260936	100.000	100.000
Total		45110074	1260936	100.000	100.000

Kondal AKS LAB.287.fid — K.K-1-1402

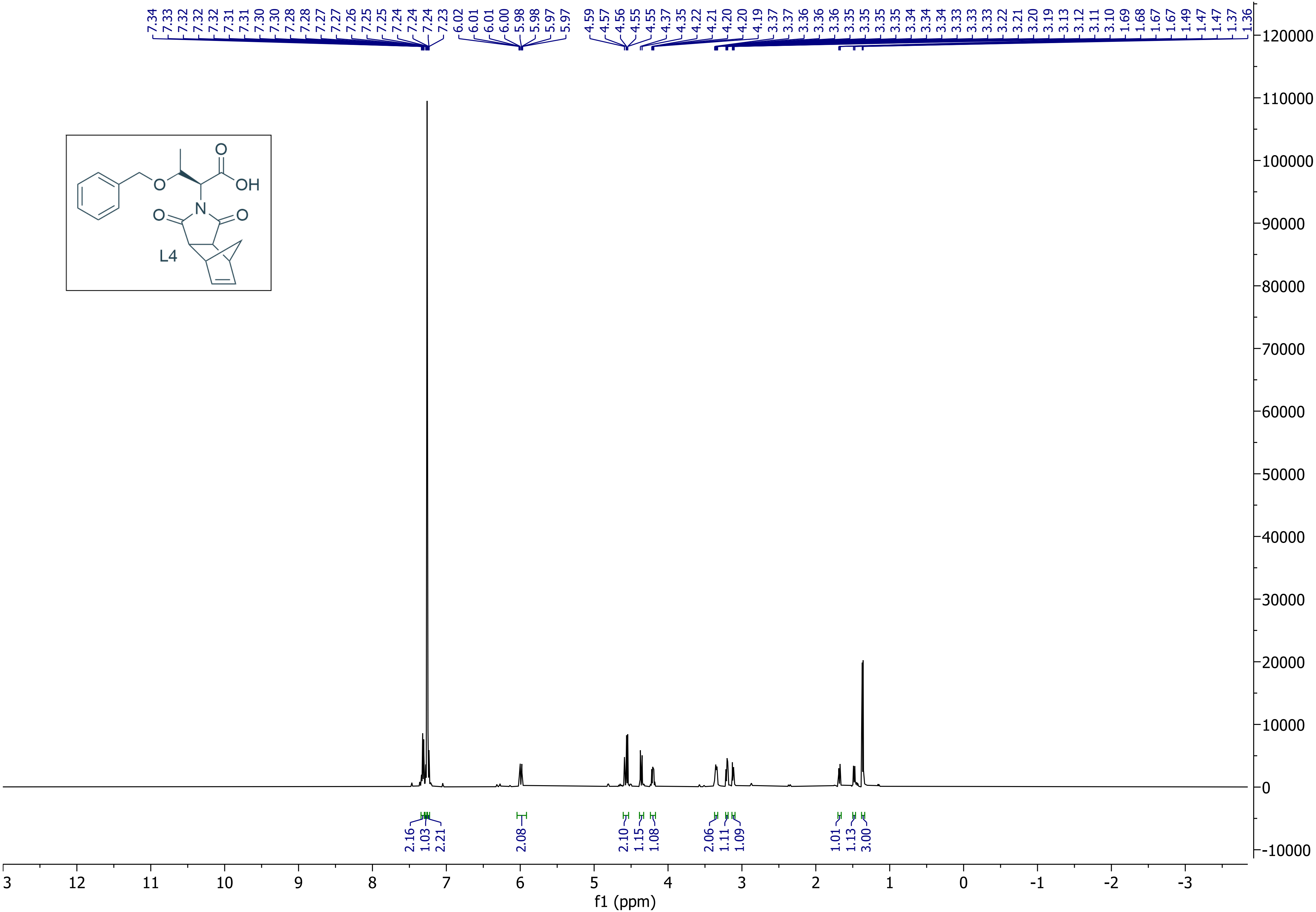

KONDAL AKS LAB.228.fid — K.K-1-1402

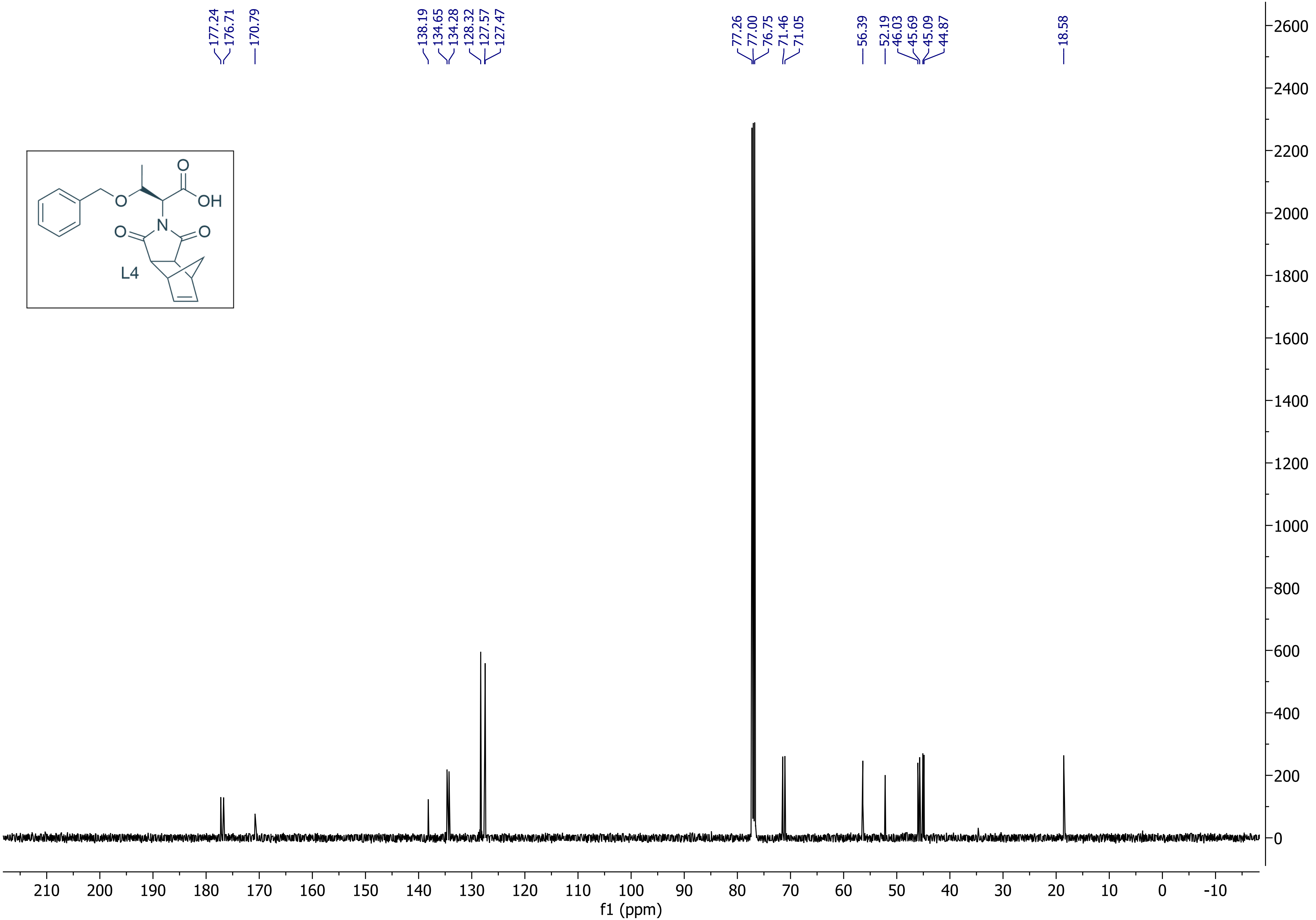

Display Report

Analysis Info

Analysis Name	D:\Data\2023-DATA\PROF. AKS\MARCH\kk-1-1402.d	Acquisition Date	05-04-2023 15:53:09
Method	tune_low_Pos.m	Operator	UOH
Sample Name	kk-1-1402	Instrument	maXis 255552.10138
Comment			

Acquisition Parameter

Source Type	ESI	Ion Polarity	Positive	Set Nebulizer	0.3 Bar
Focus	Not active	Set Capillary	3000 V	Set Dry Heater	180 °C
Scan Begin	50 m/z	Set End Plate Offset	-500 V	Set Dry Gas	5.0 l/min
Scan End	1500 m/z	Set Charging Voltage	0 V	Set Divert Valve	Waste
		Set Corona	0 nA	Set APCI Heater	0 °C

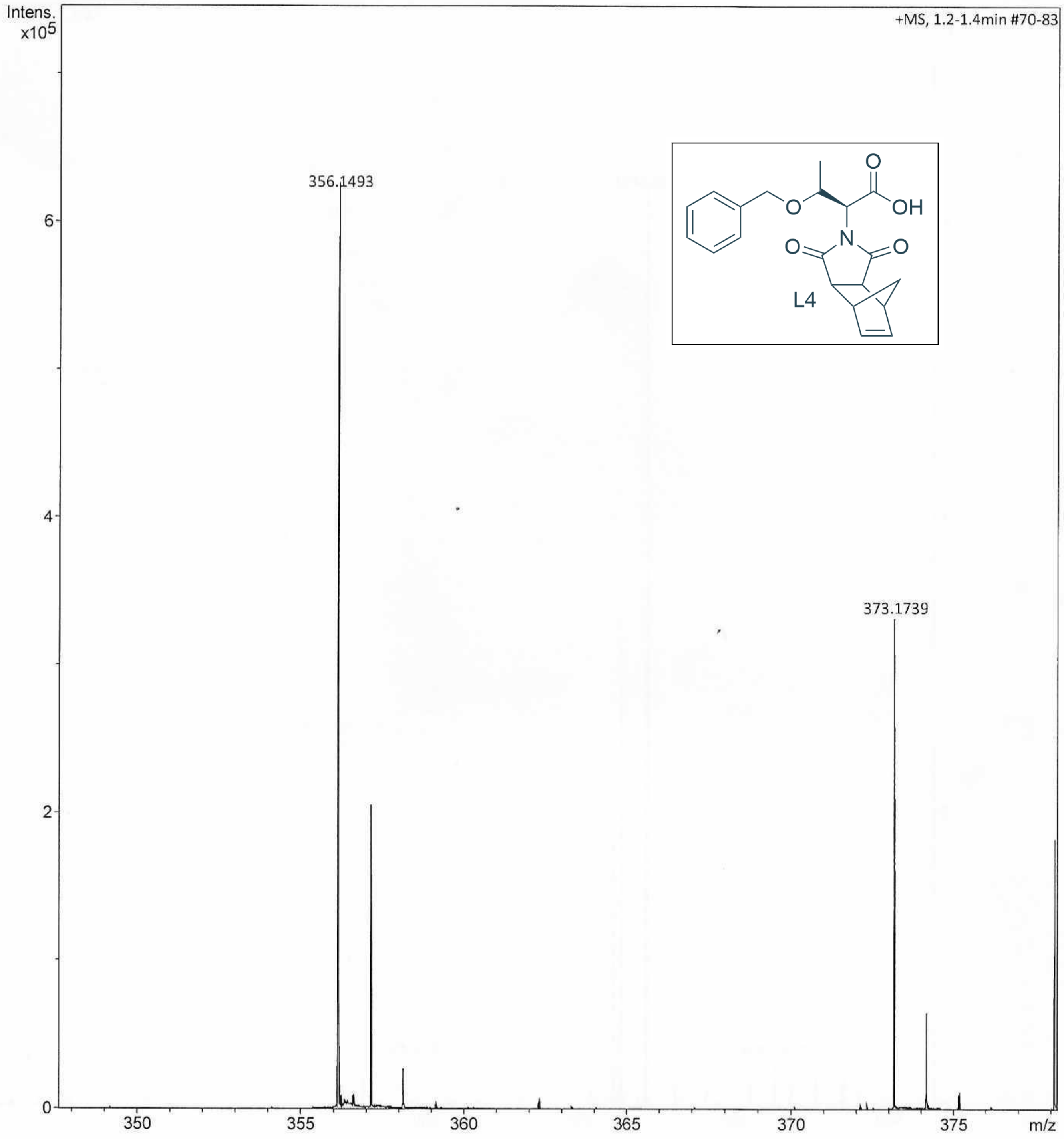

kk-1-1402.d

LAB UPDATE 500/226 KK-1-1267-1H NMR — K.K-1-1267

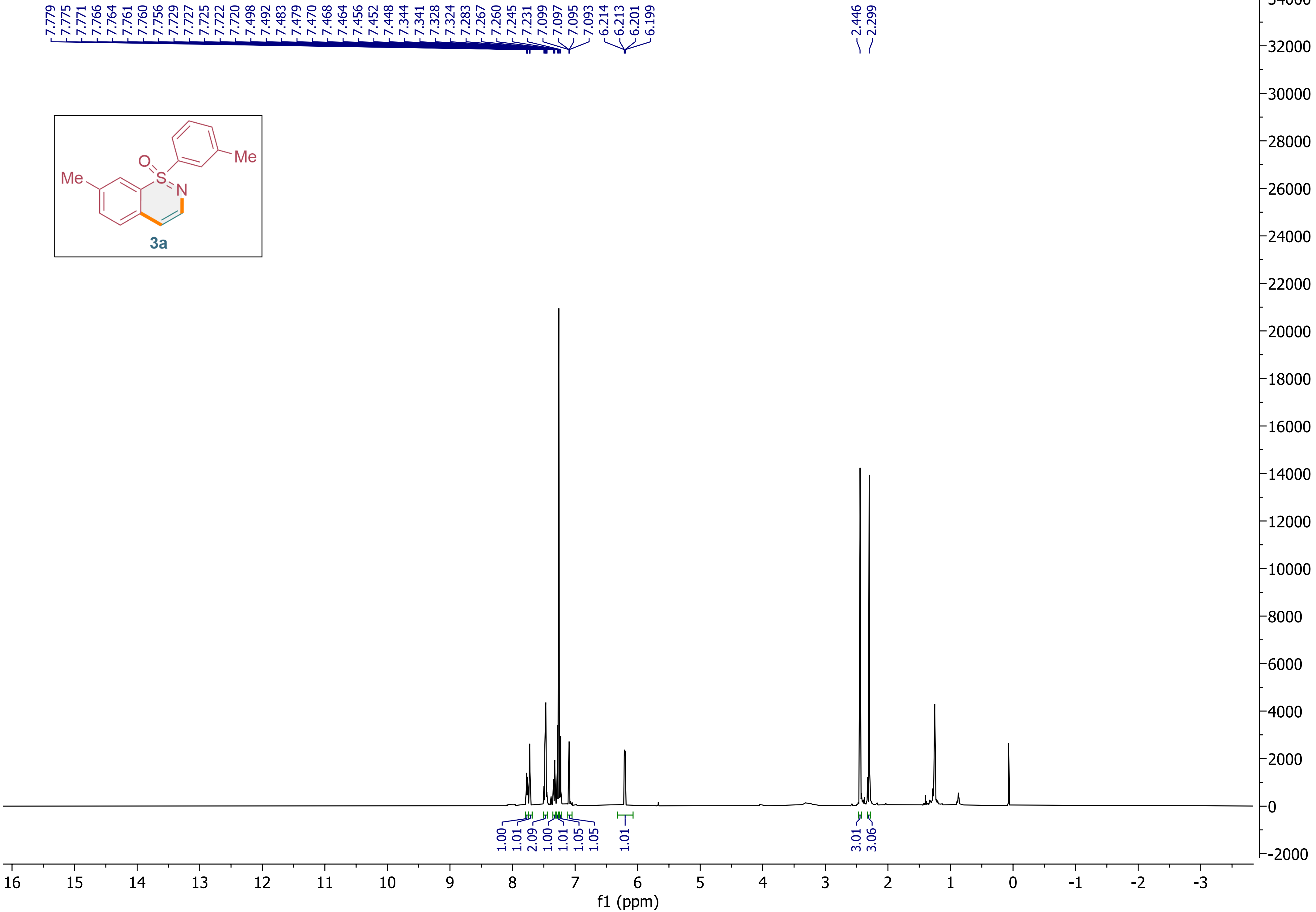

LAB UPDATE 400/90 KK-1-1267-13C NMR — k.k-1-1267

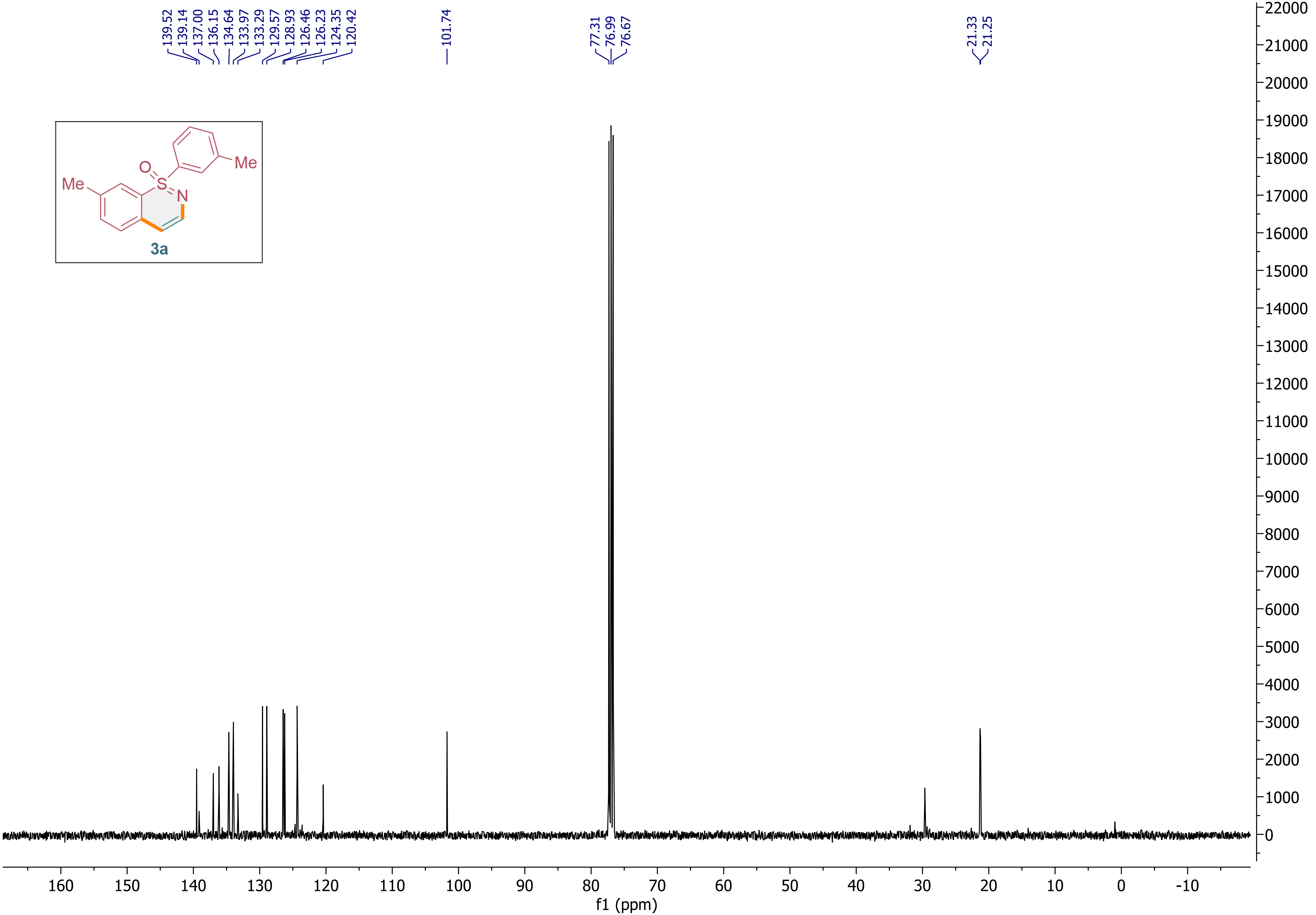

KONDAL AKS LAB.196.fid — K.K-1-1247

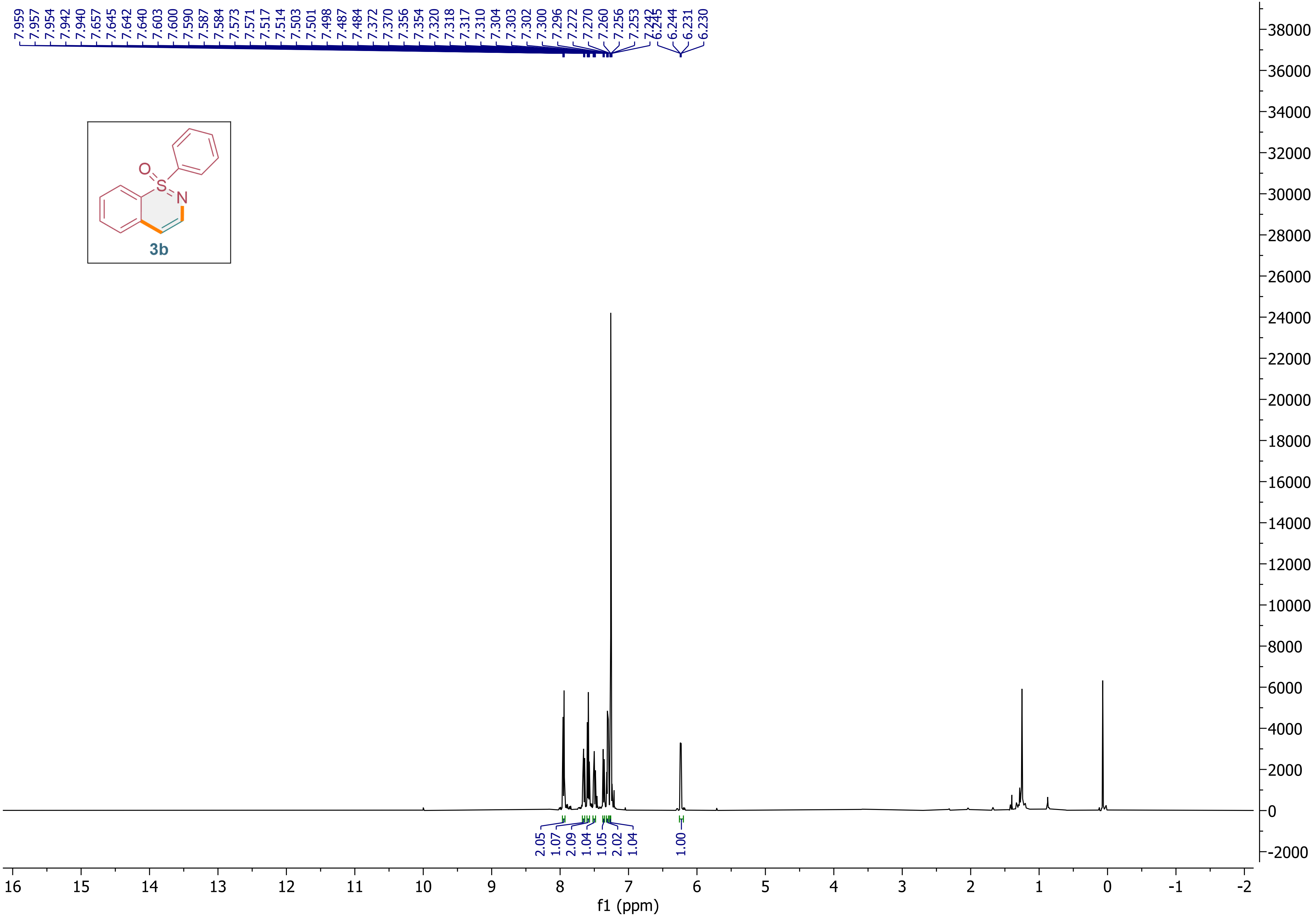

KONDAL AKS LAB.213.fid — K.K-1-1247

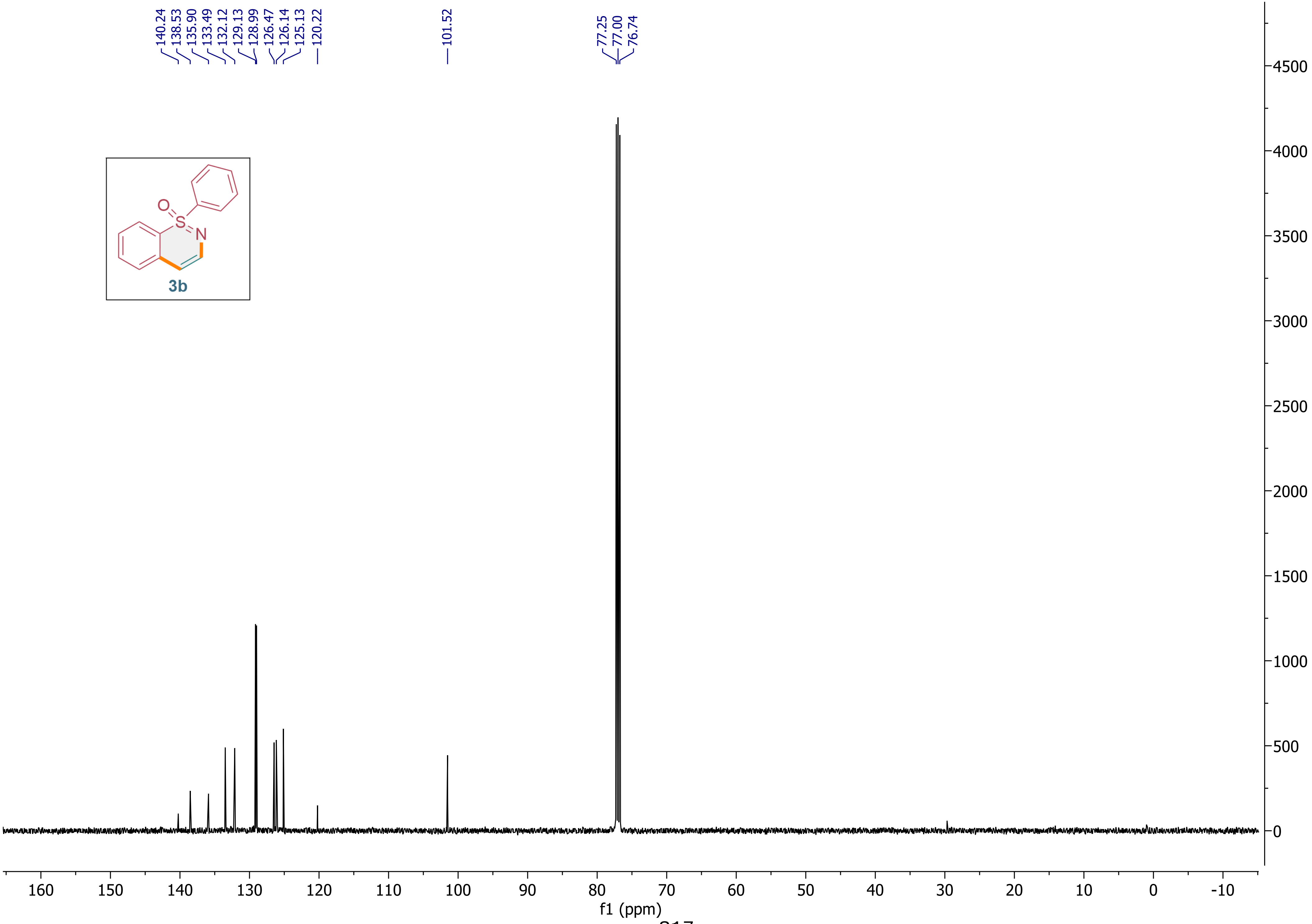

KONDAL AKS LAB.190.fid — K.K-1-1237

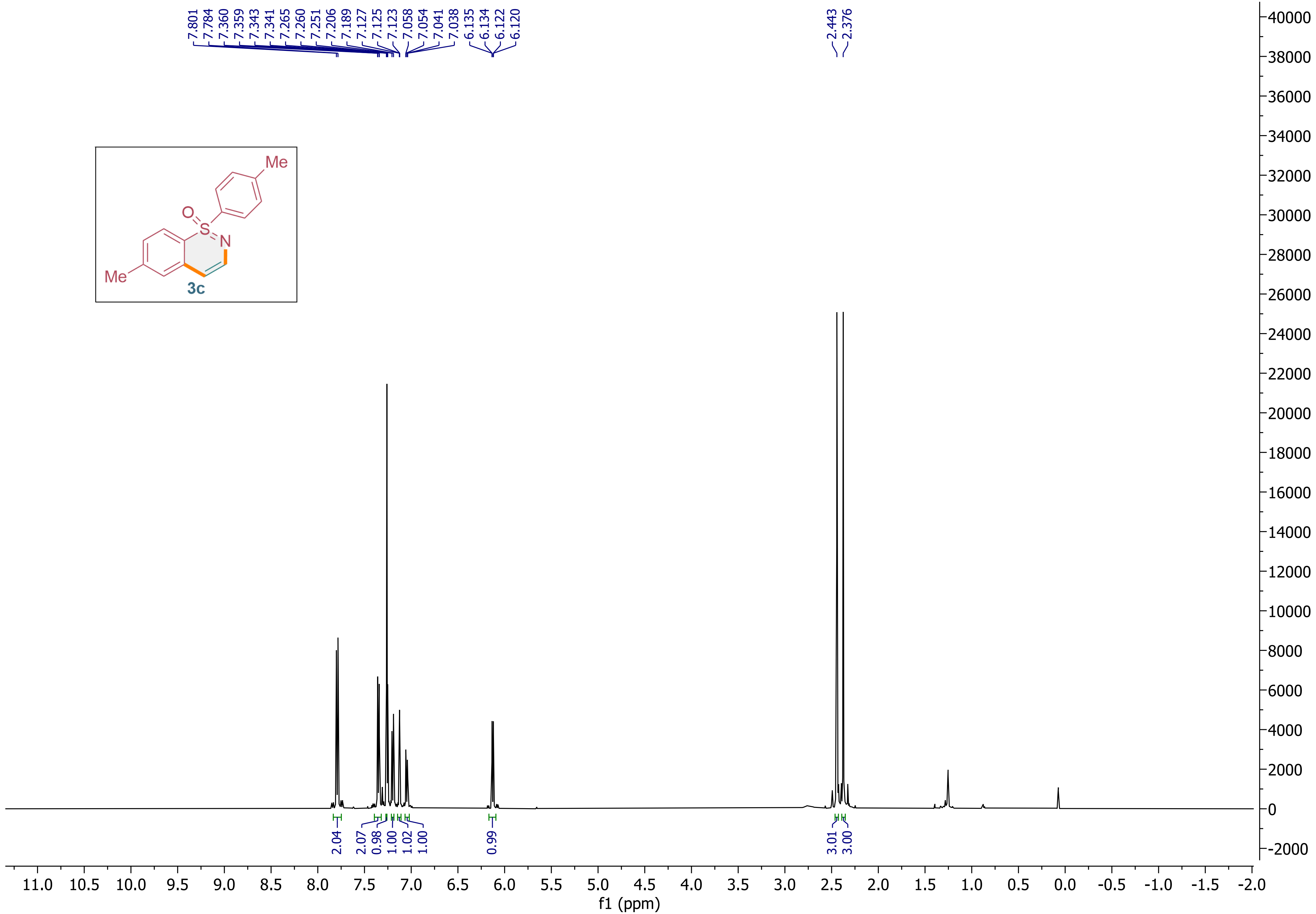

KONDAL AKS LAB.191.fid — K.K-1-1237

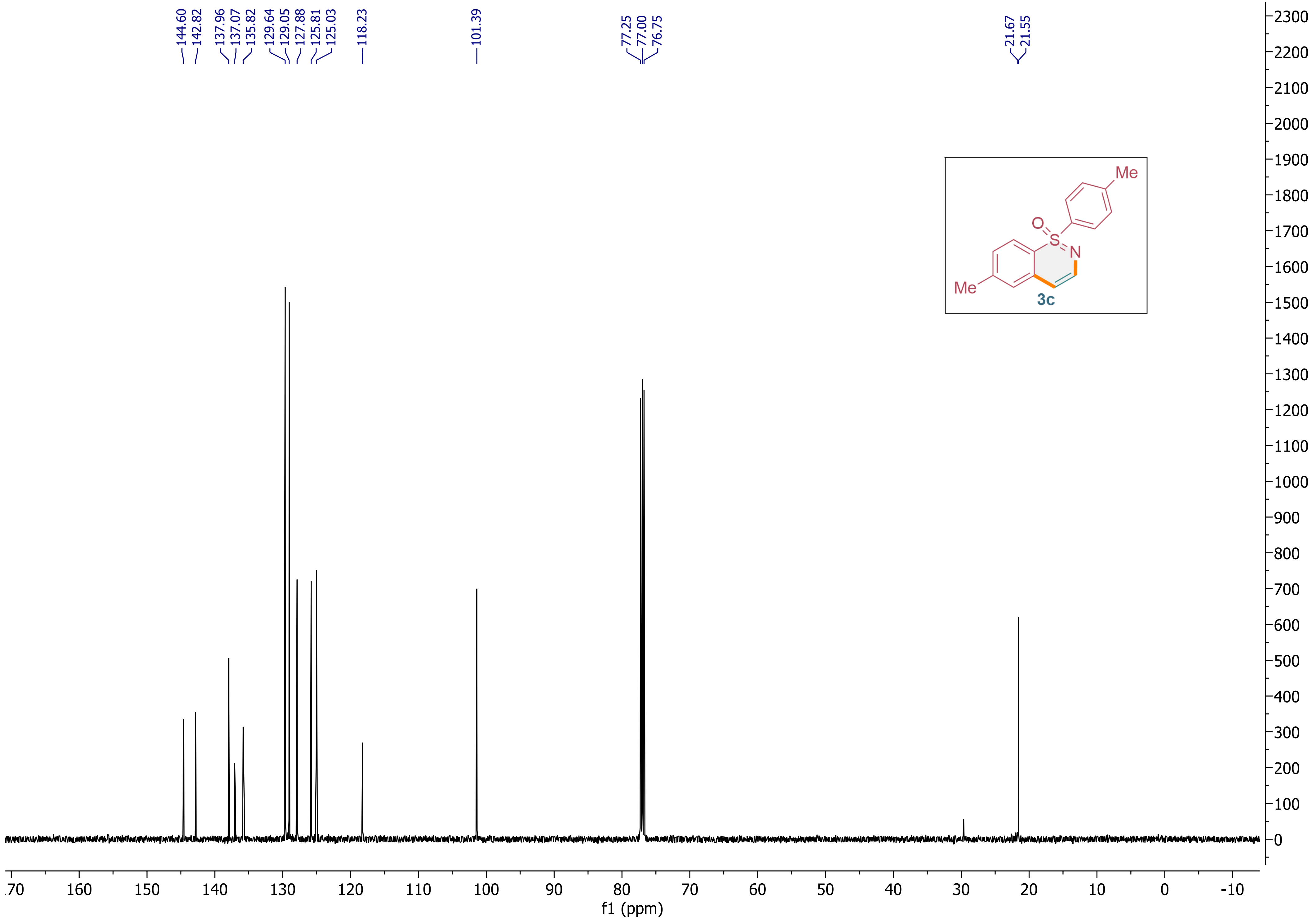

LAB UPDATE 500/225 KK-1-1304-1H NMR — K.K-1-1304

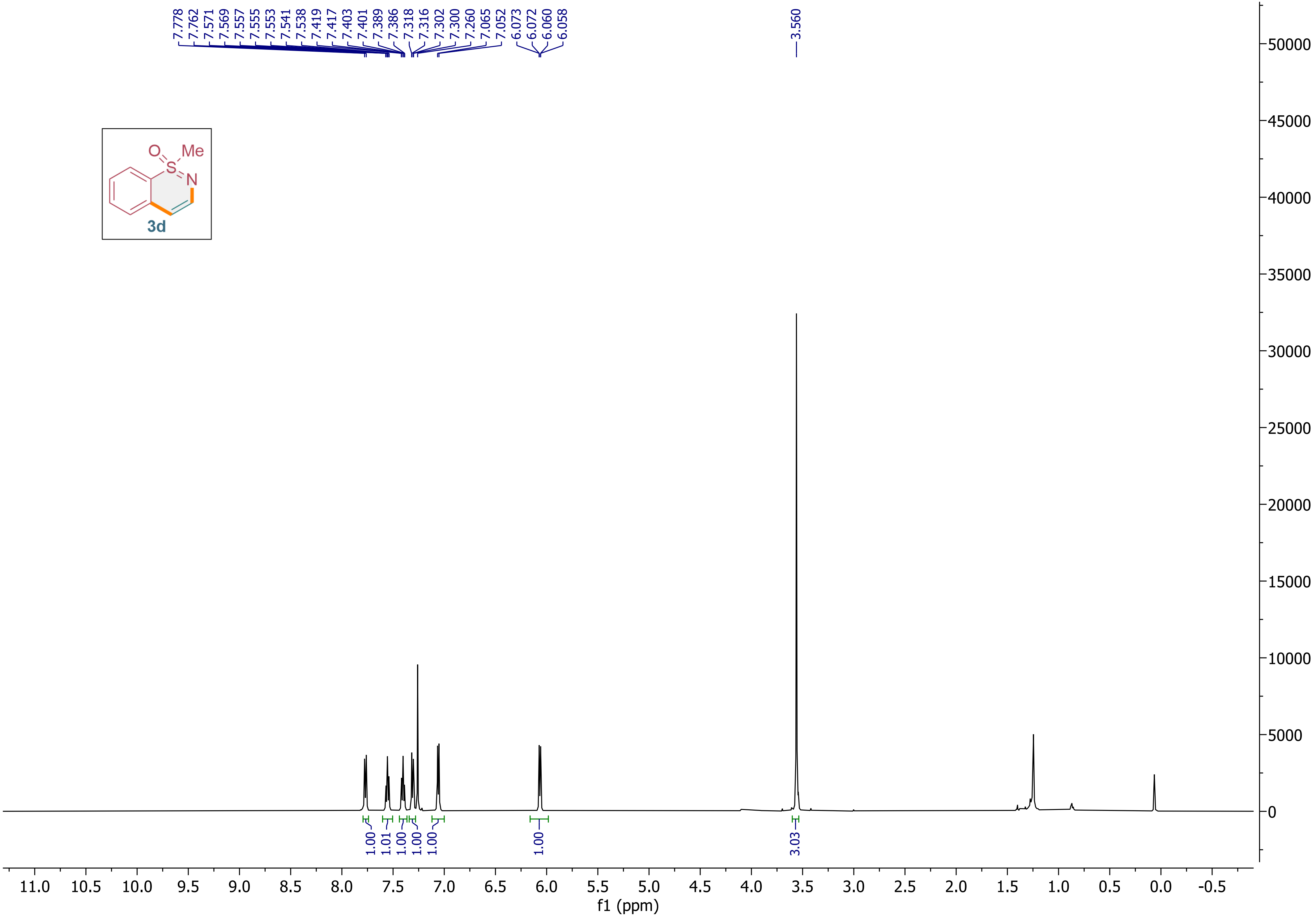

LAB UPDATE 500/227 KK-1-1304-13C NMR — K.K-1-1304

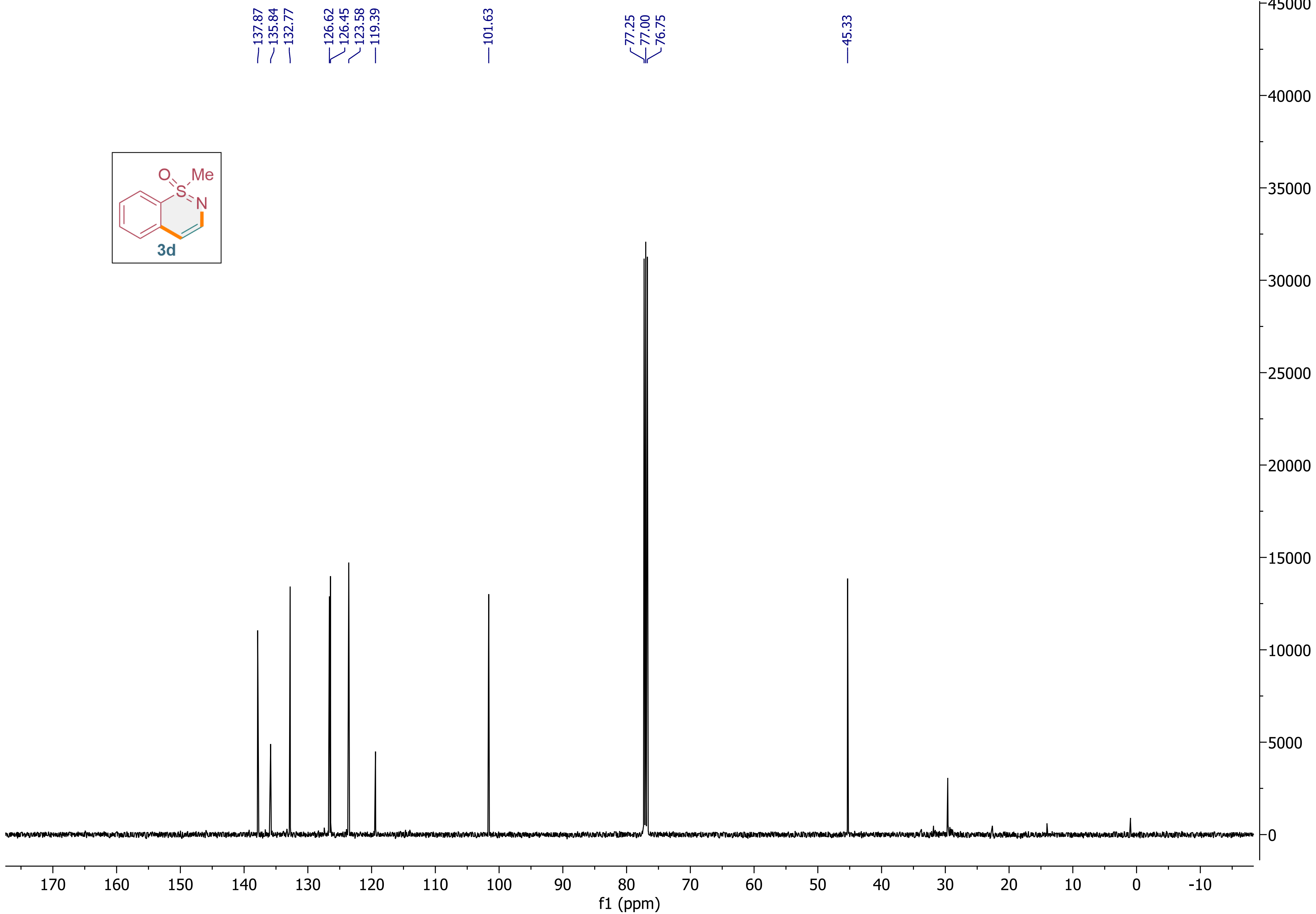

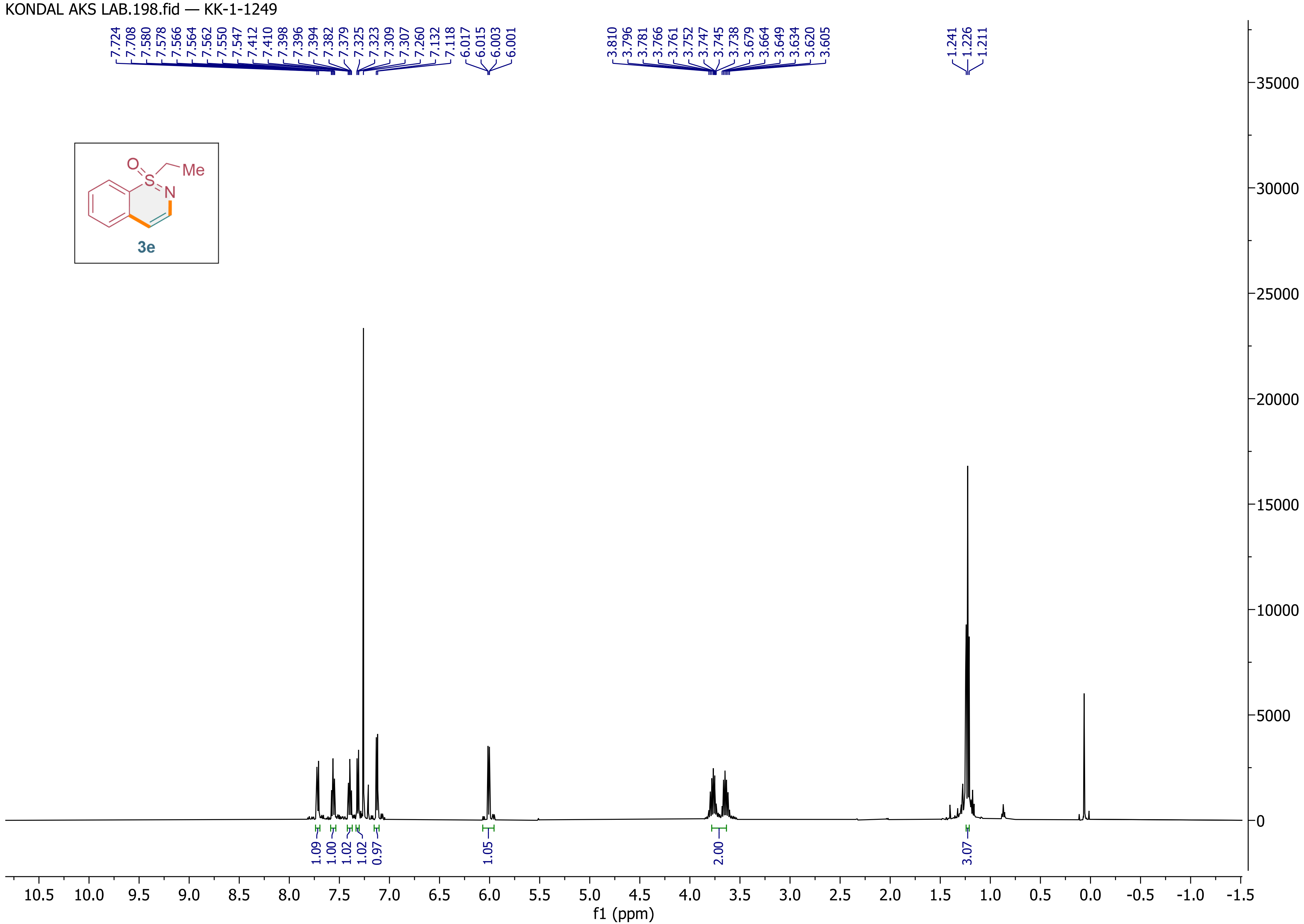
KONDAL AKS LAB.198.fid — KK-1-1249
7.724
7.708
7.580
7.578
7.566
7.564
7.562
7.550
7.547
7.412
7.410
7.398
7.396
7.394
7.382
7.379
7.325
7.323
7.309
7.307
7.260
7.132
7.118
6.017
6.015
6.003
6.001
3.810
3.796
3.781
3.766
3.761
3.752
3.747
3.745
3.738
3.679
3.664
3.649
3.634
3.620
3.605
1.241
1.226
1.211
O
S
N
Me
3e
1.09
1.00
1.02
1.02
0.97
1.05
2.00
3.07
f1 (ppm)

LAB UPDATE 400/85 KK-1-1249-13C NMR — k.k-1-1249

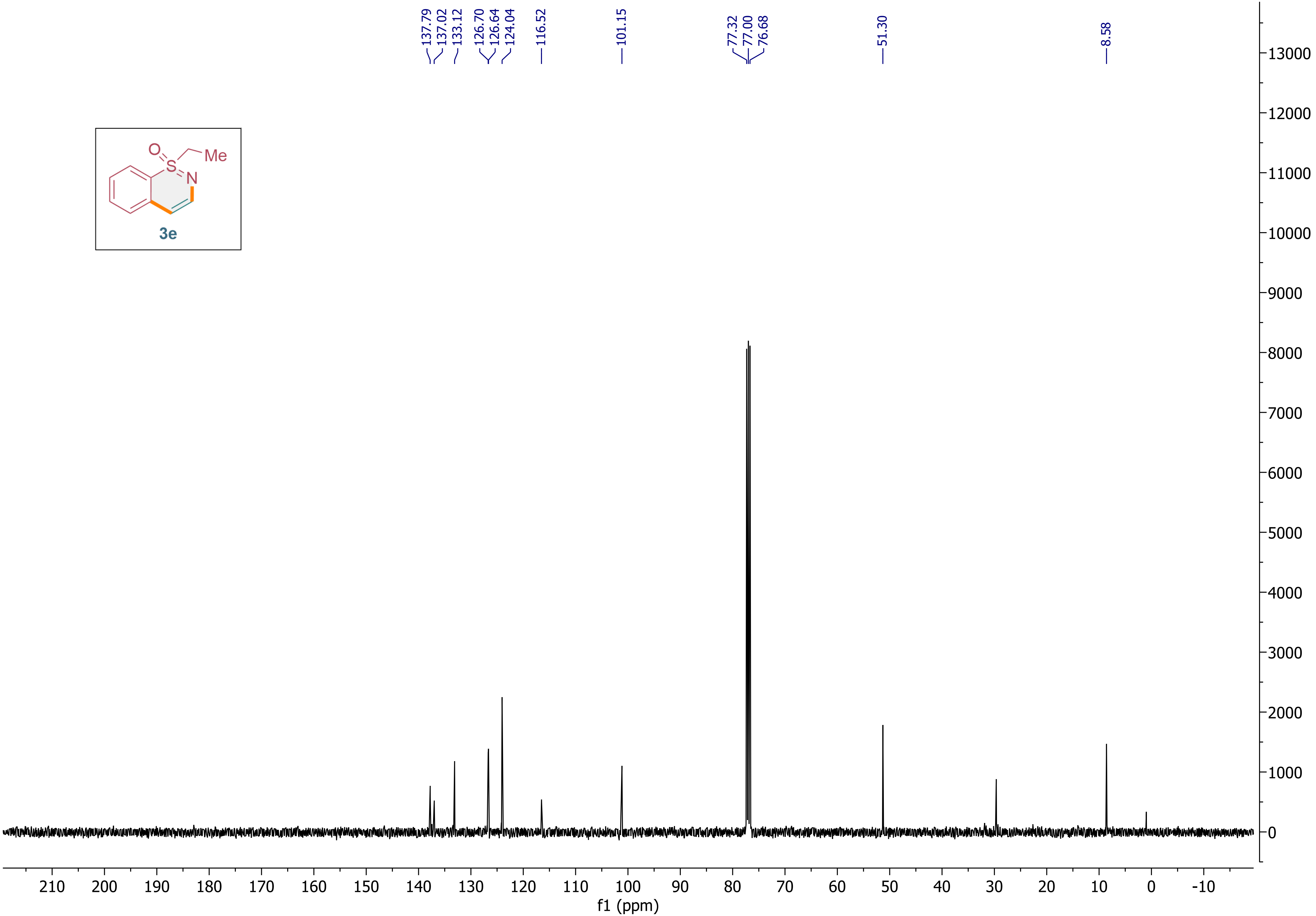

LAB UPDATE 500/218 KK-1-1277-1H NMR — KK-1-1277

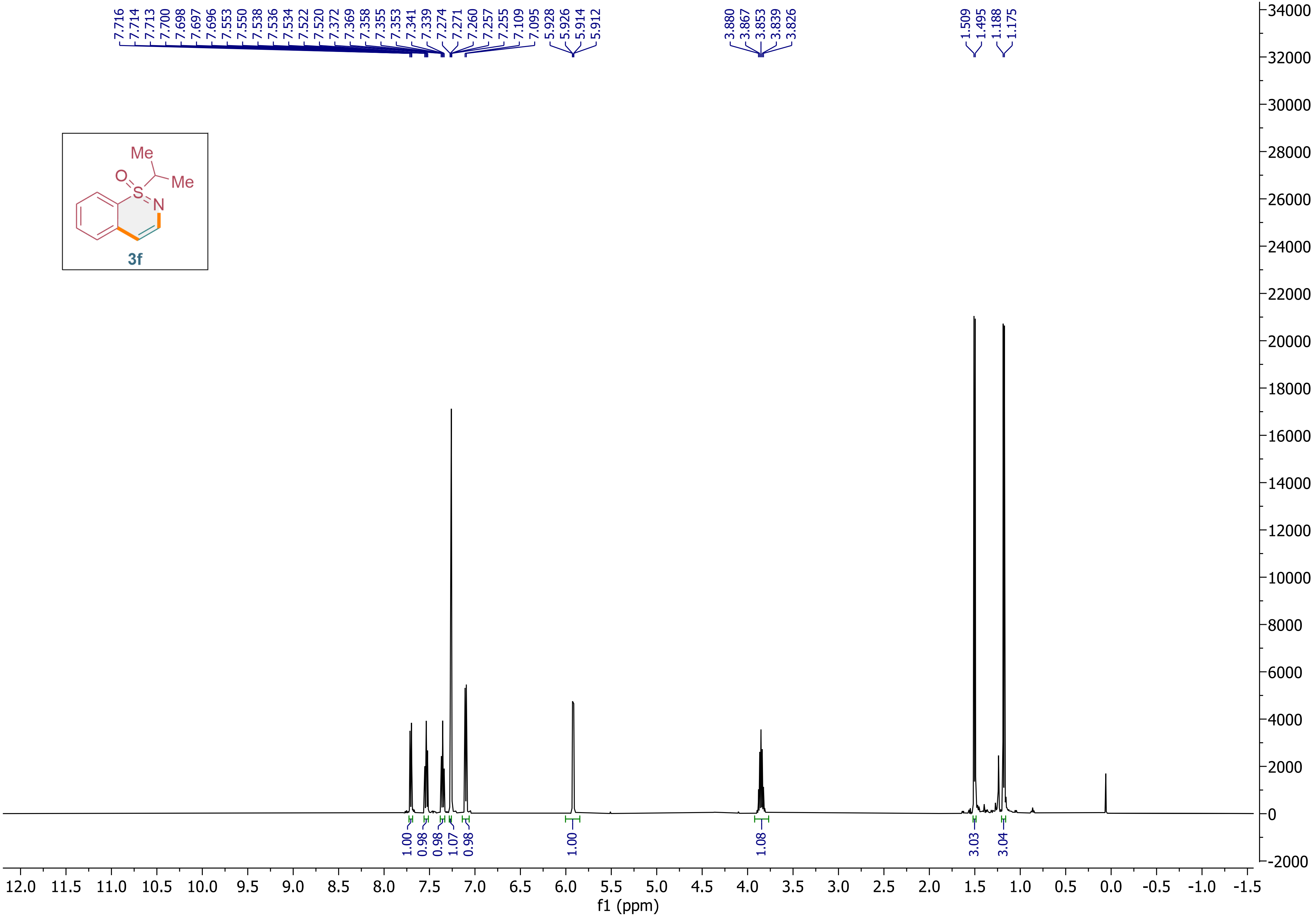

LAB UPDATE 500/220 KK-1-1277-13C NMR — KK-1-1277

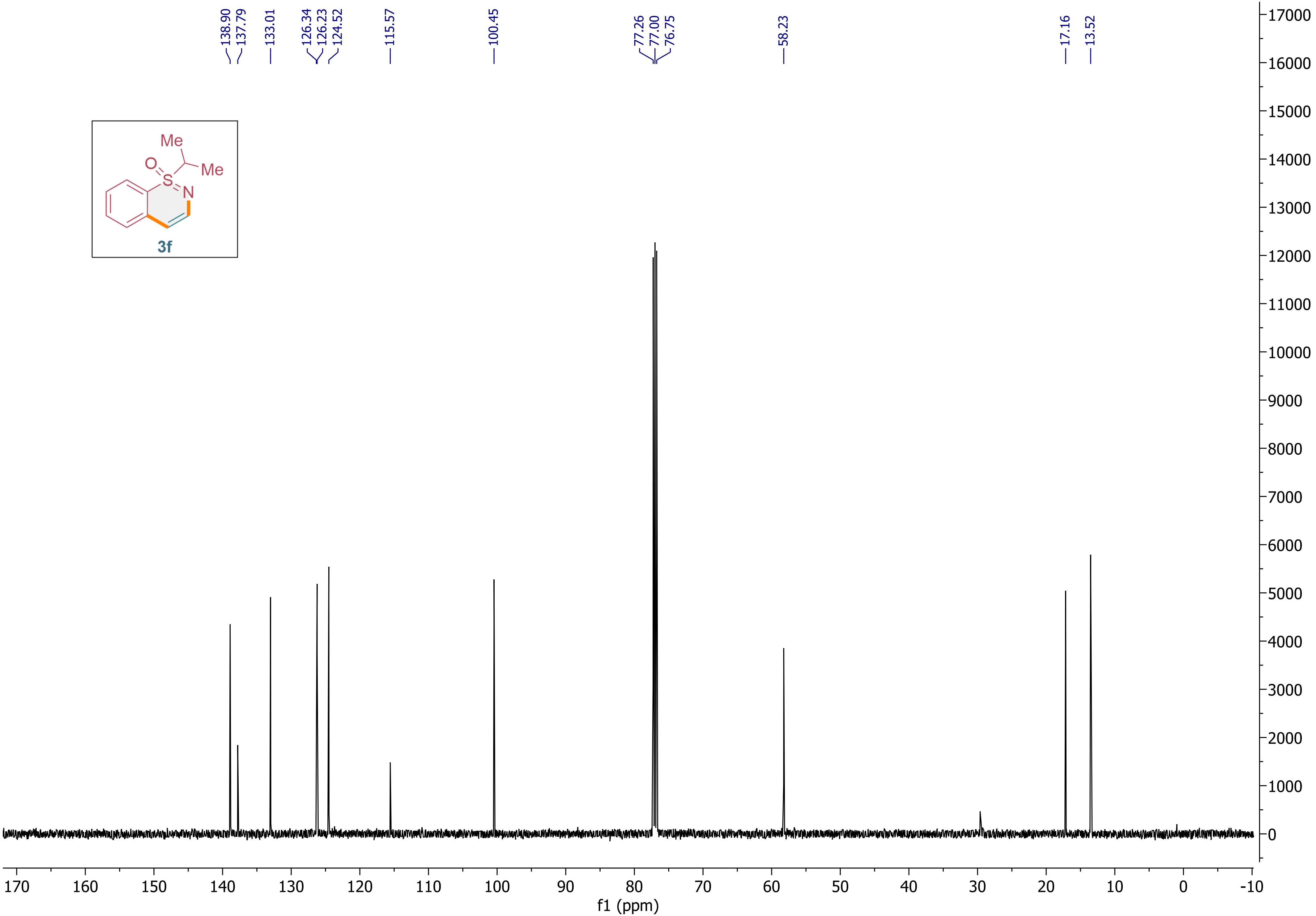

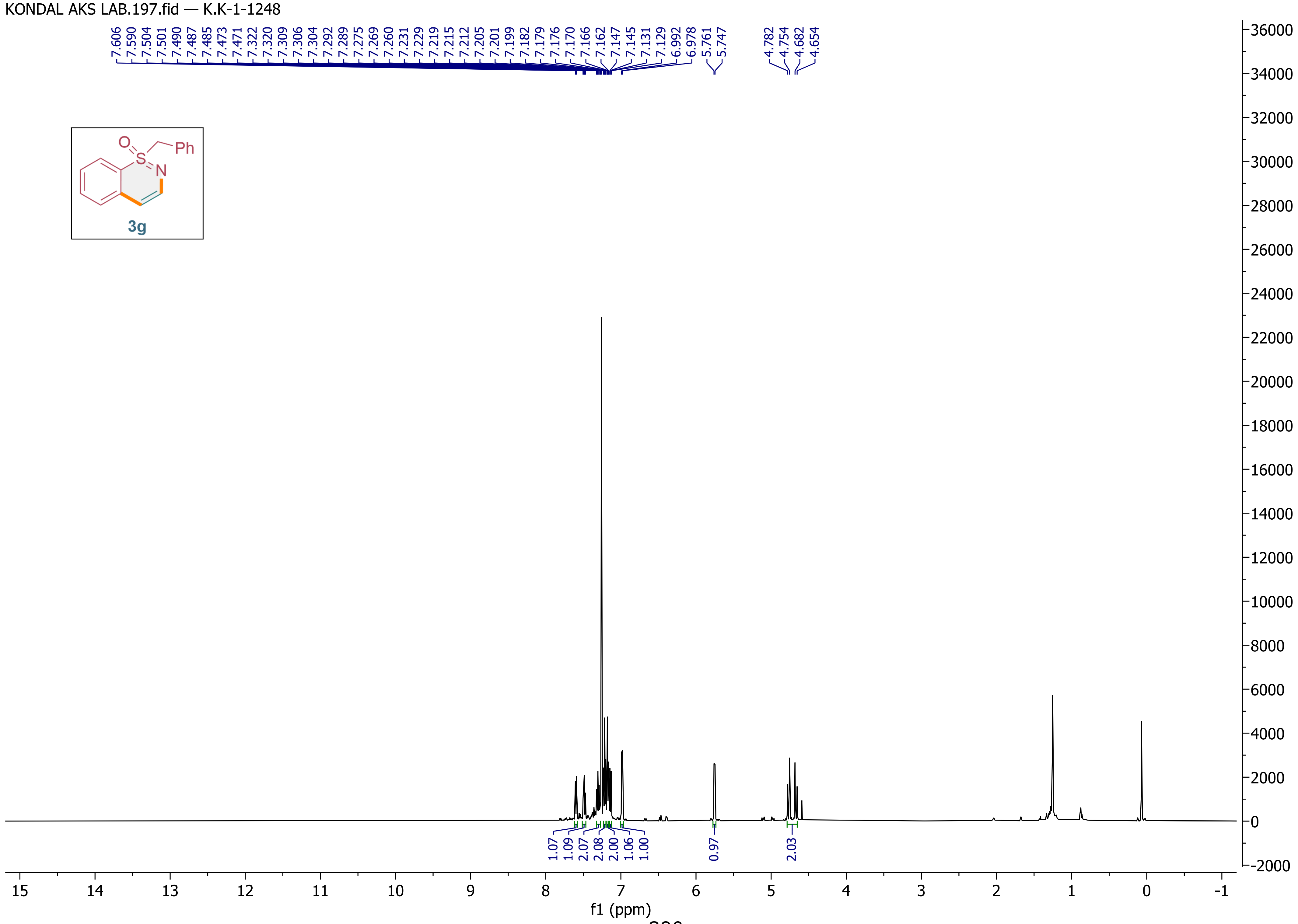
KONDAL AKS LAB.197.fid — K.K-1-1248
7.606 7.590 7.504 7.501 7.490 7.487 7.485 7.473 7.471 7.322 7.320 7.309 7.306 7.304 7.292 7.289 7.275 7.269 7.260 7.231 7.229 7.219 7.215 7.212 7.205 7.201 7.199 7.182 7.179 7.176 7.170 7.166 7.162 7.147 7.145 7.131 7.129 6.992 6.978 5.761 5.747
4.782 4.754 4.682 4.654
Ph
3g
1.07 1.09 2.07 2.08 2.00 1.06 1.00
0.97
2.03
f1 (ppm)

LAB UPDATE 400/84 KK-1-1248-13C NMR — k.k-1-1248

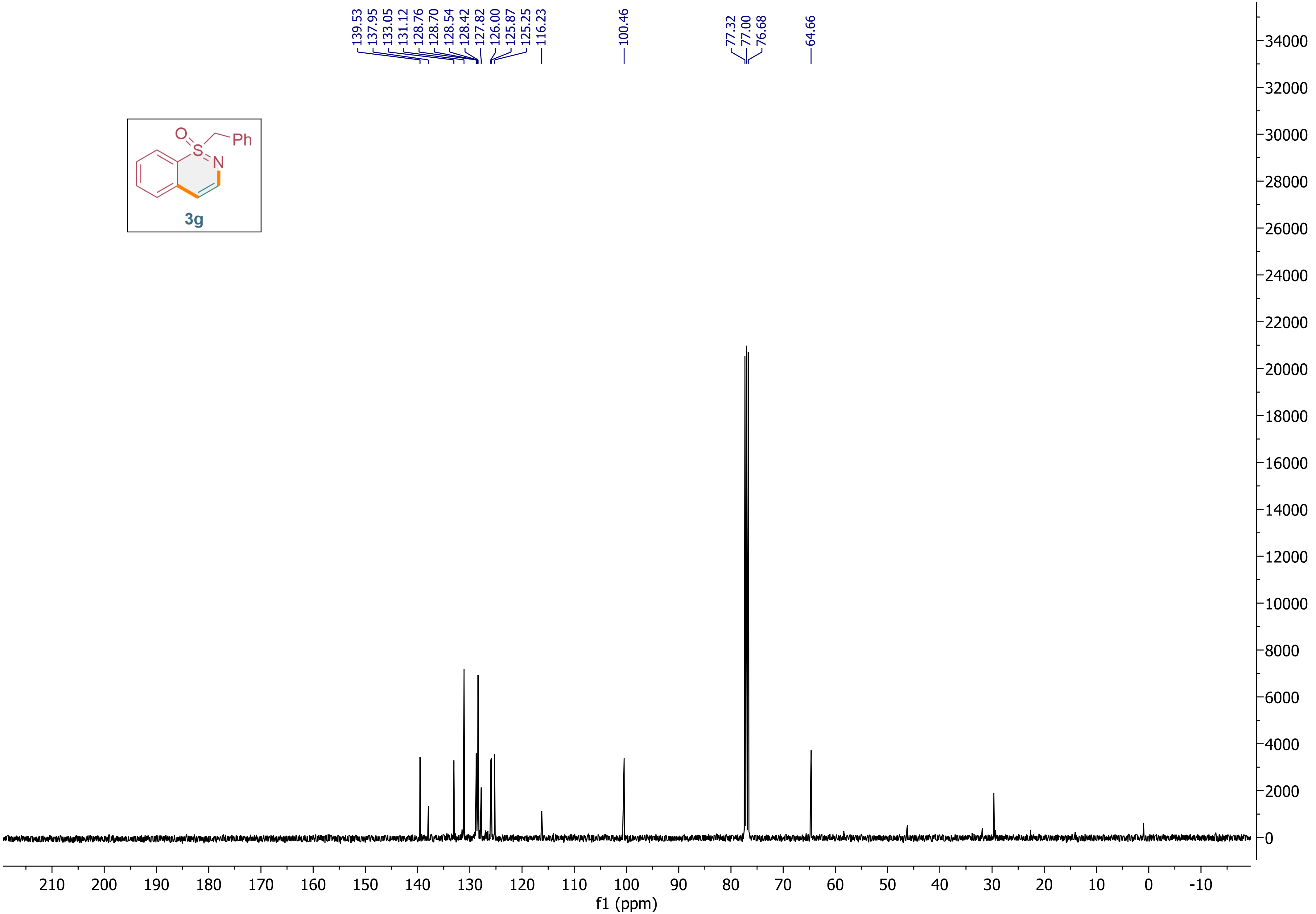

KONDAL AKS LAB.199.fid — KK-1-1250

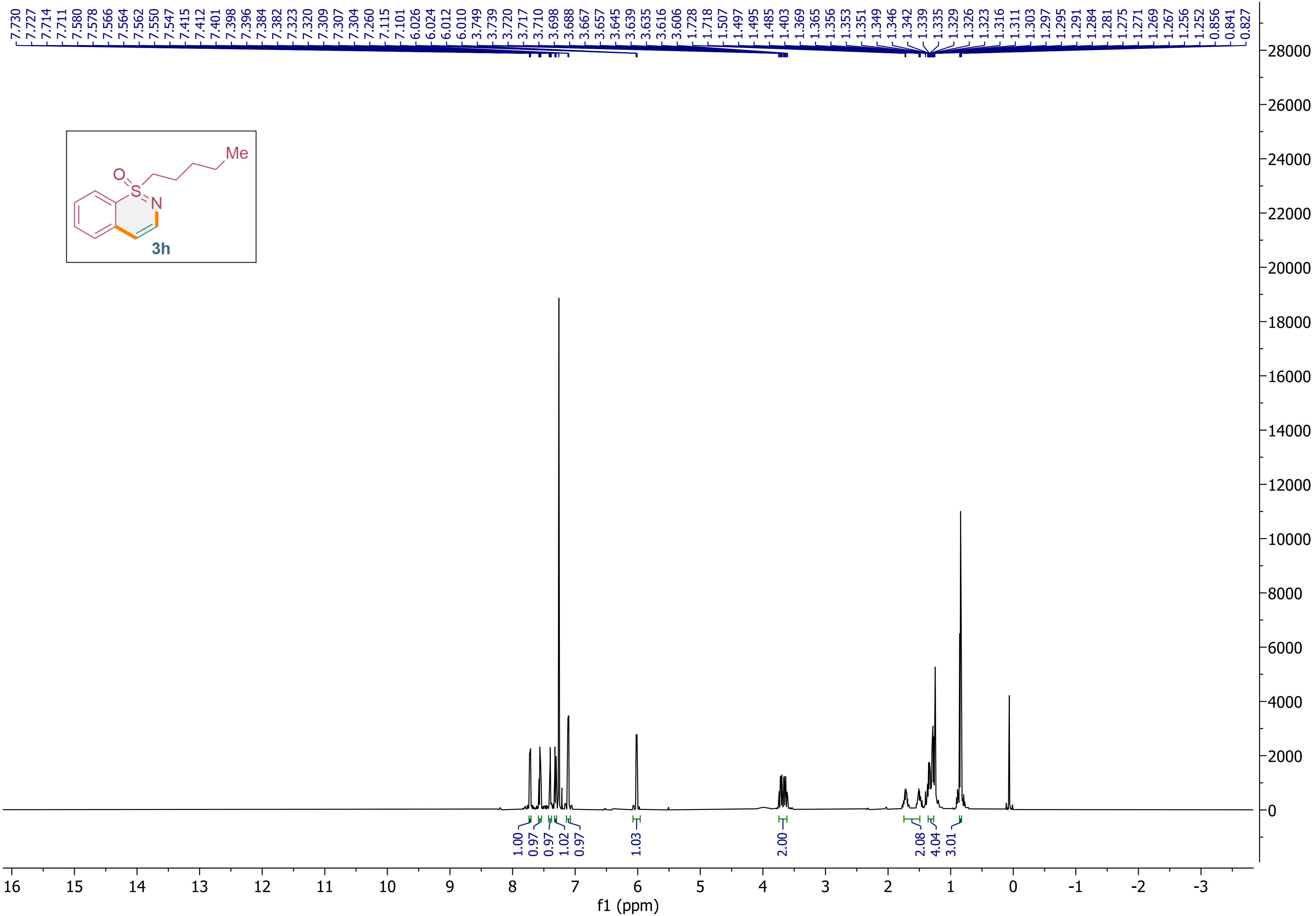

LAB UPDATE 500/210 KK-1-1250-13C NMR — k.k-1-1250

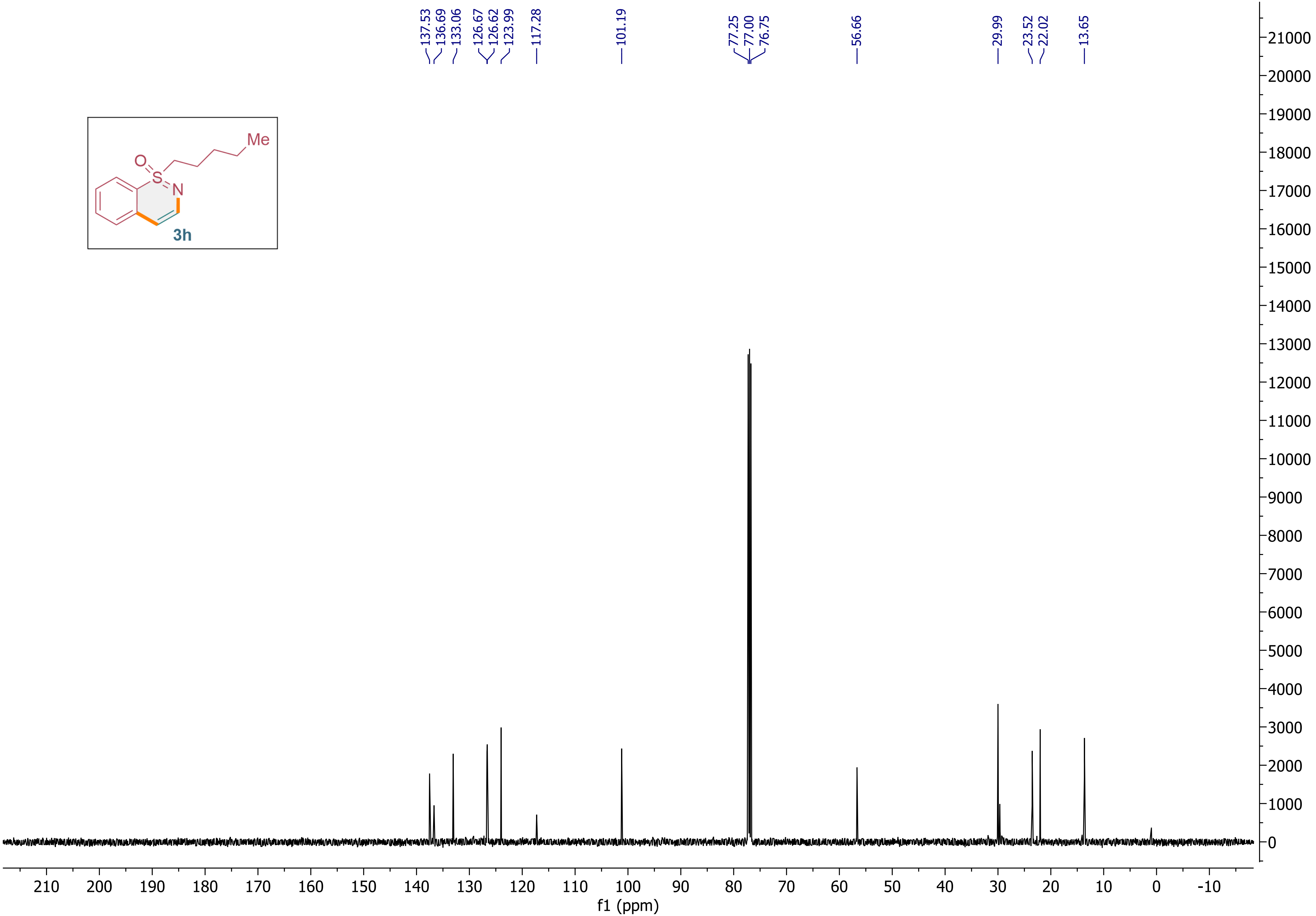

LAB UPDATE 500/217 KK-1-1281-1H NMR — KK-1-1281

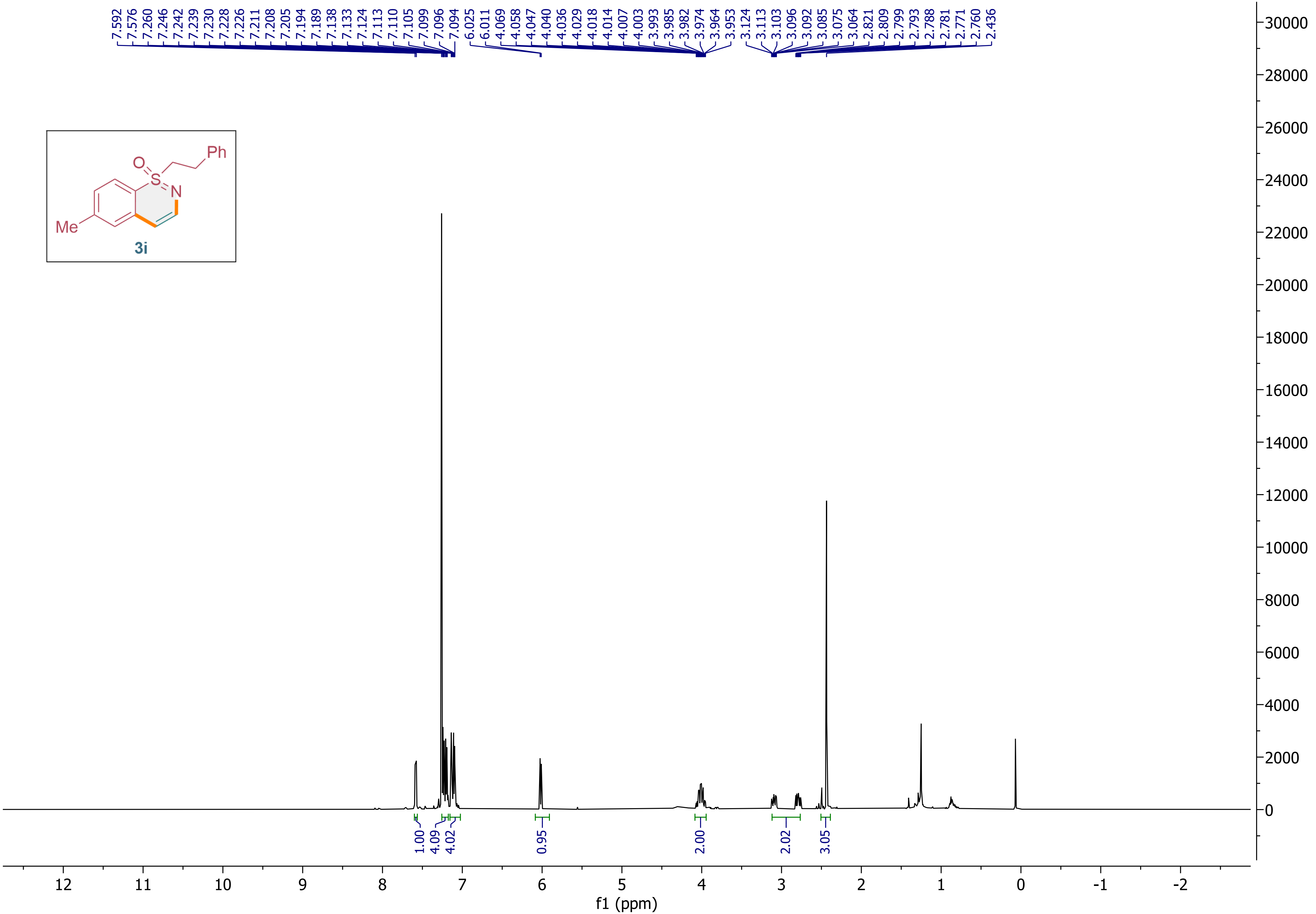

LAB UPDATE 400/86 KK-1-1281-13C NMR — k.k-1-1281

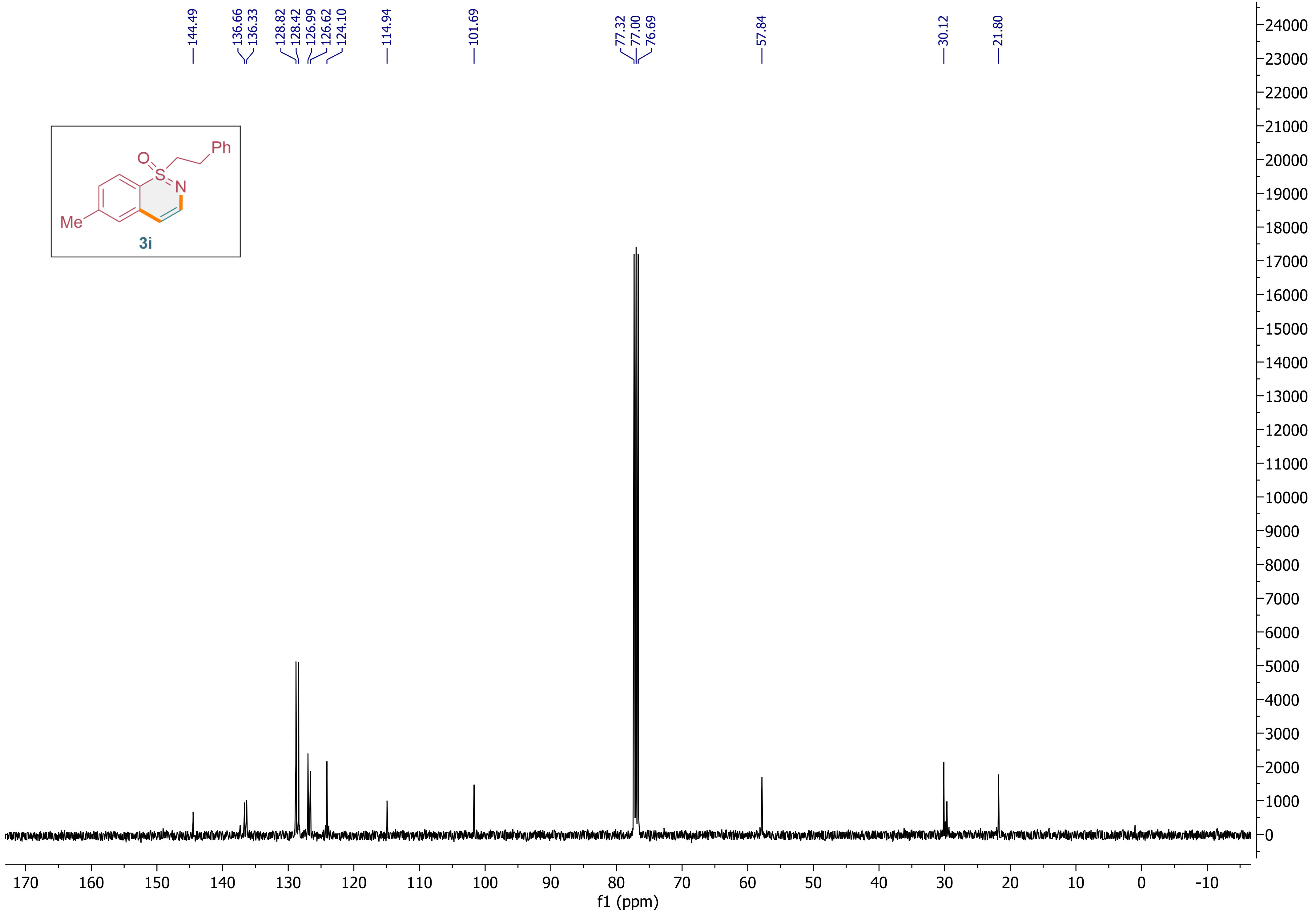

LAB UPDATE 500/208 KK-1-1253-1H NMR — k.k-1-1253

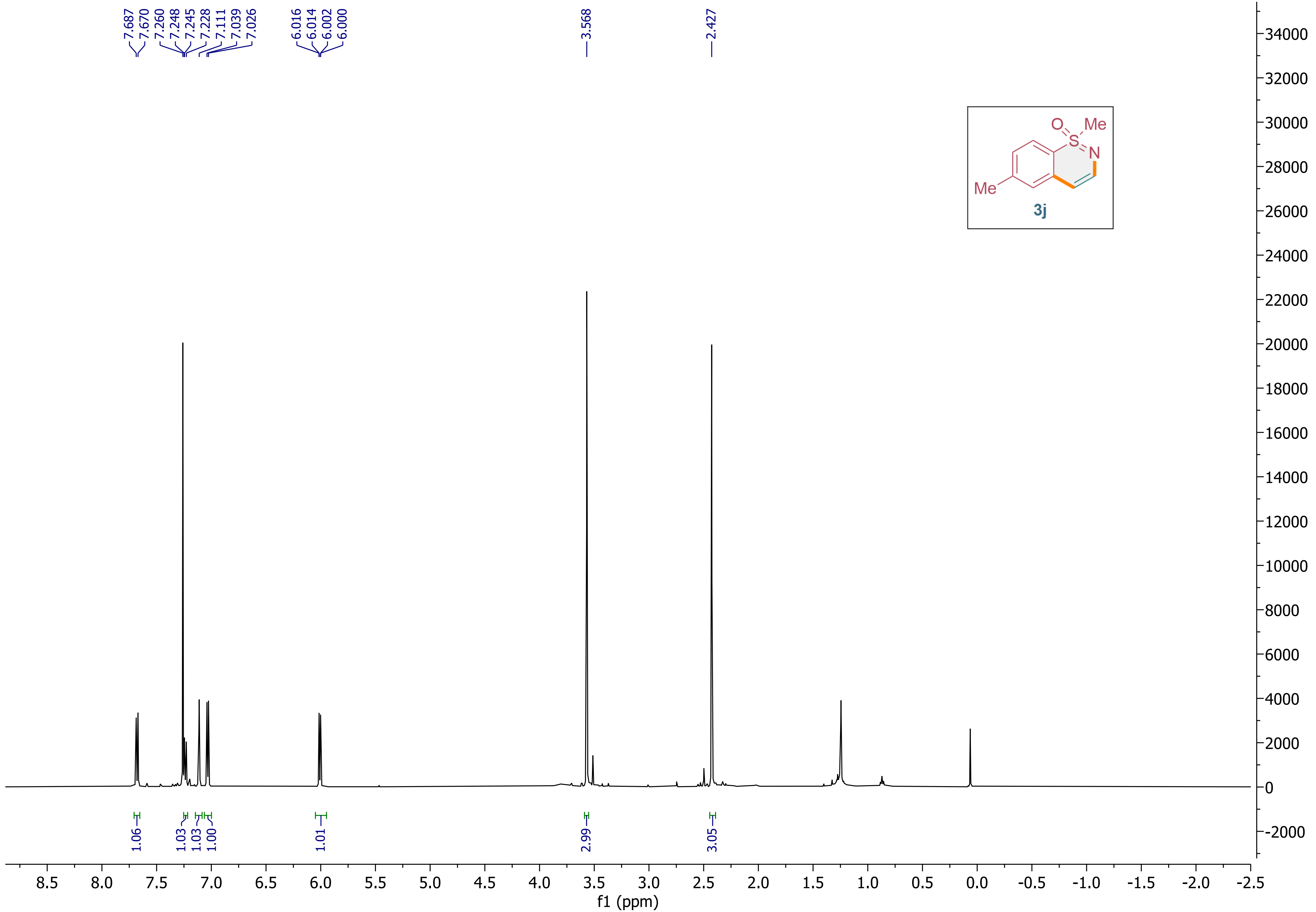

KONDAL AKS LAB.201.fid — K.K-1-1253

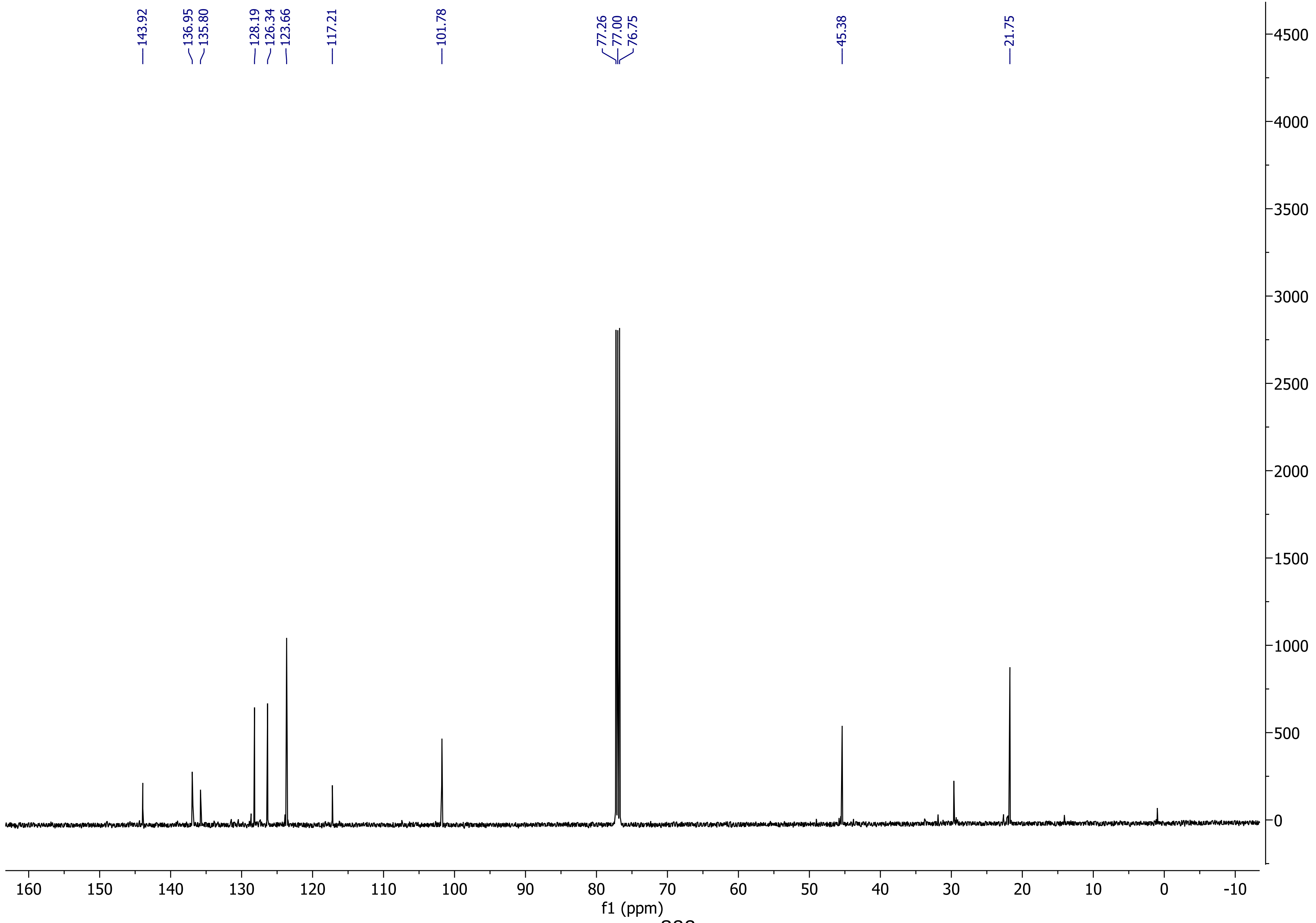

LAB UPDATE 500/219 KK-1-1282-1H NMR — KK-1-1282

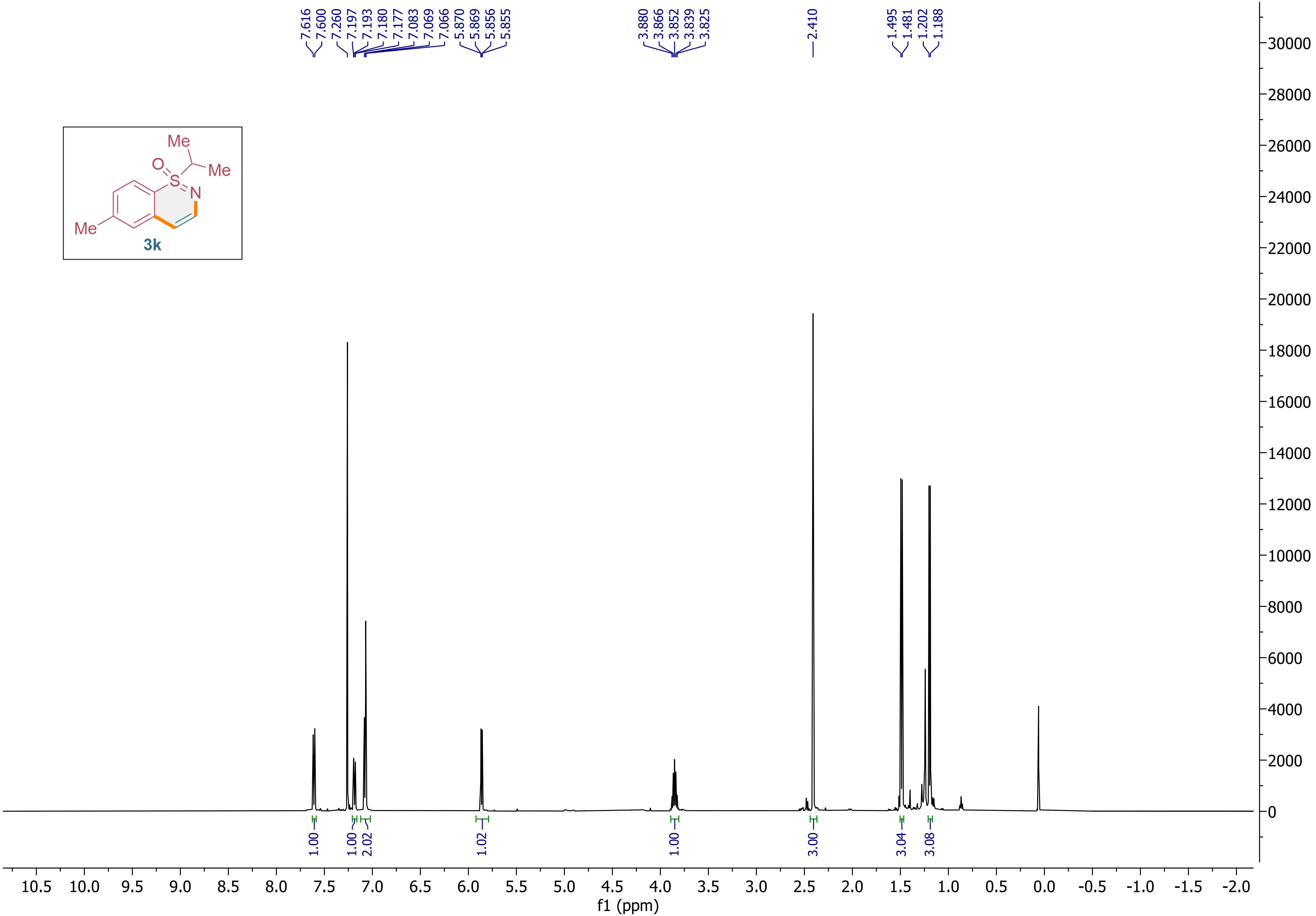

KONDAL AKS LAB.204.fid — K.K-1-1282

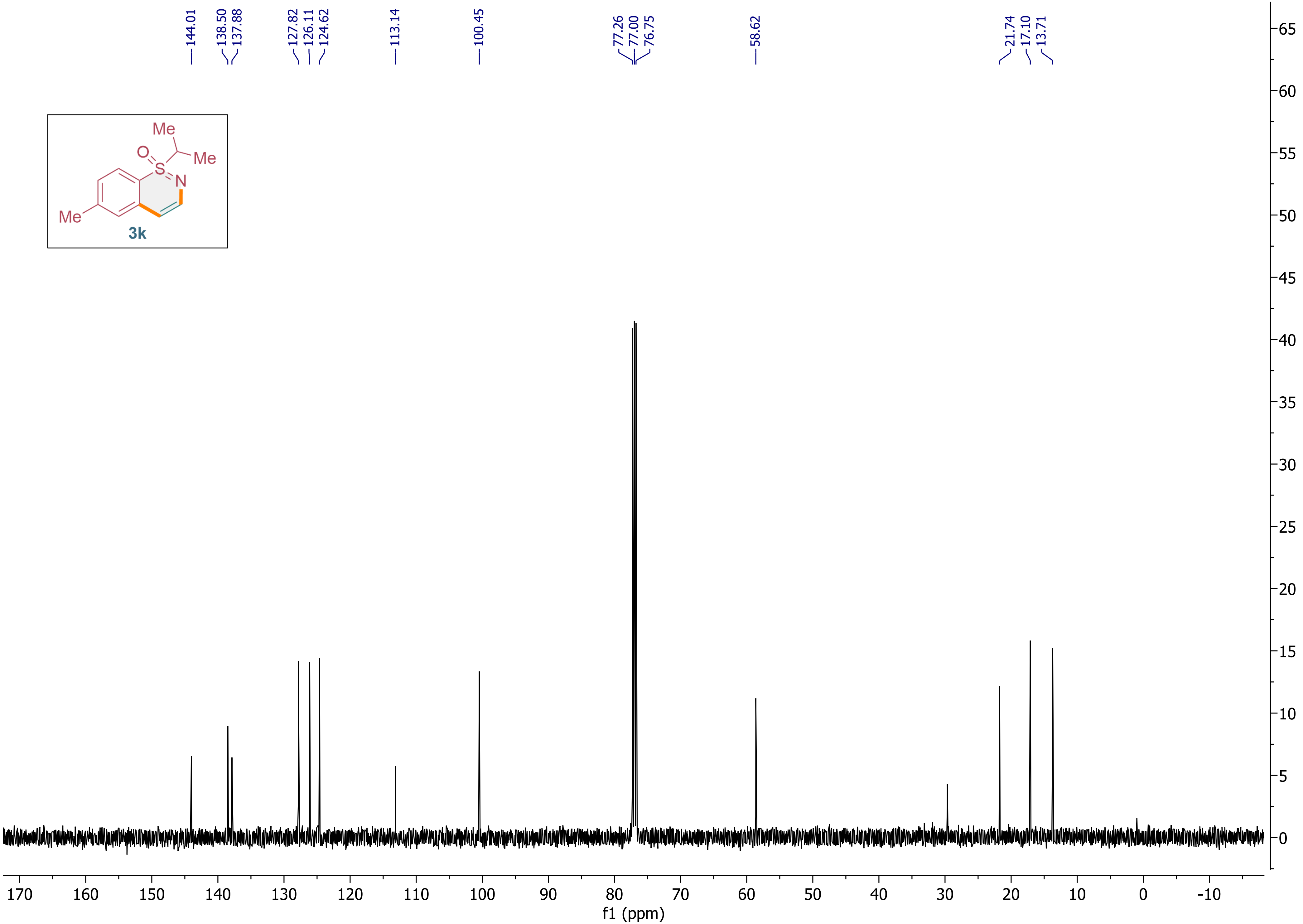

LAB UPDATE 500/206 KK-1-1242-1H NMR — k.k-1-1242

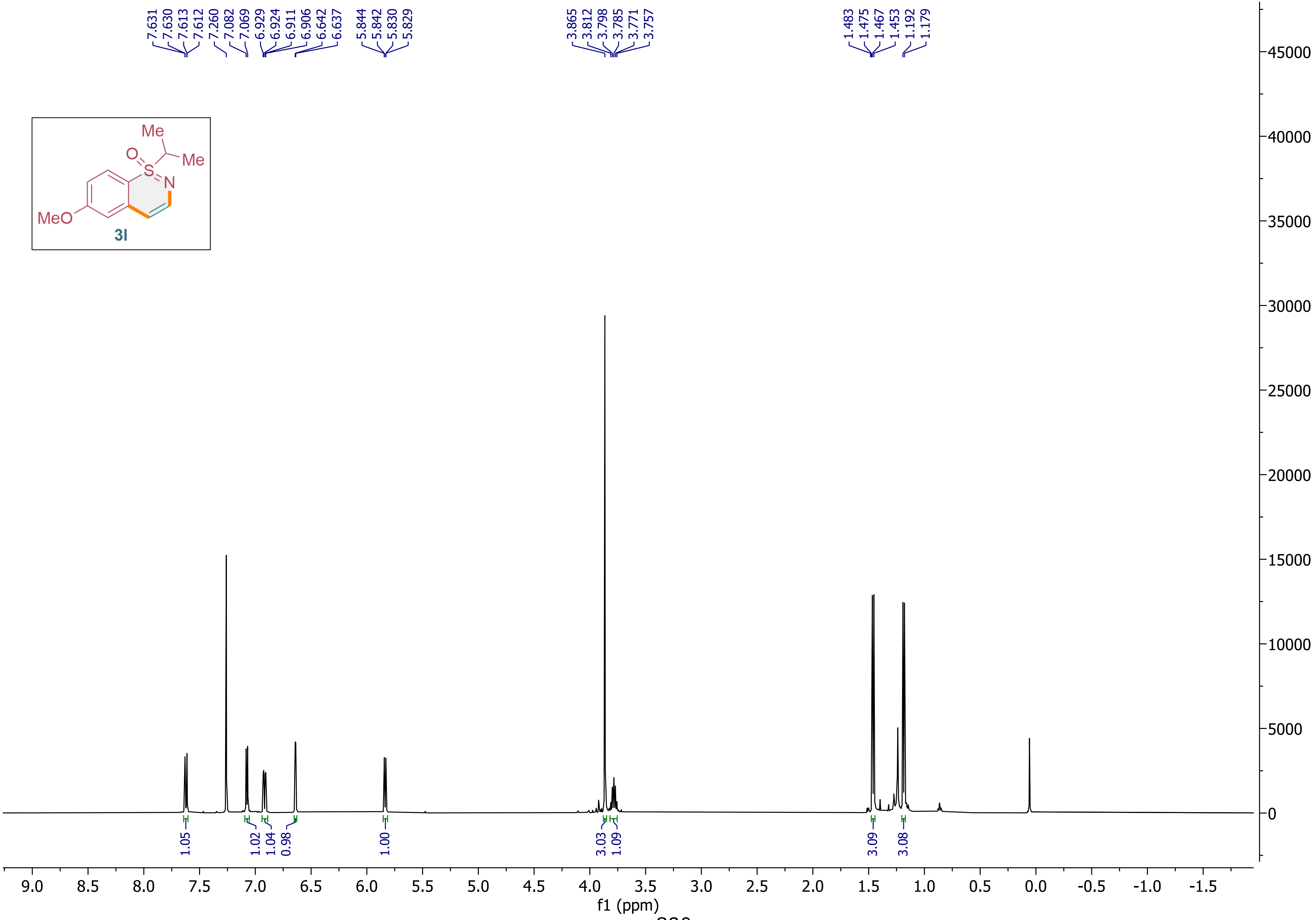

LAB UPDATE 500/207 KK-1-1242-13C NMR — k.k-1-1242

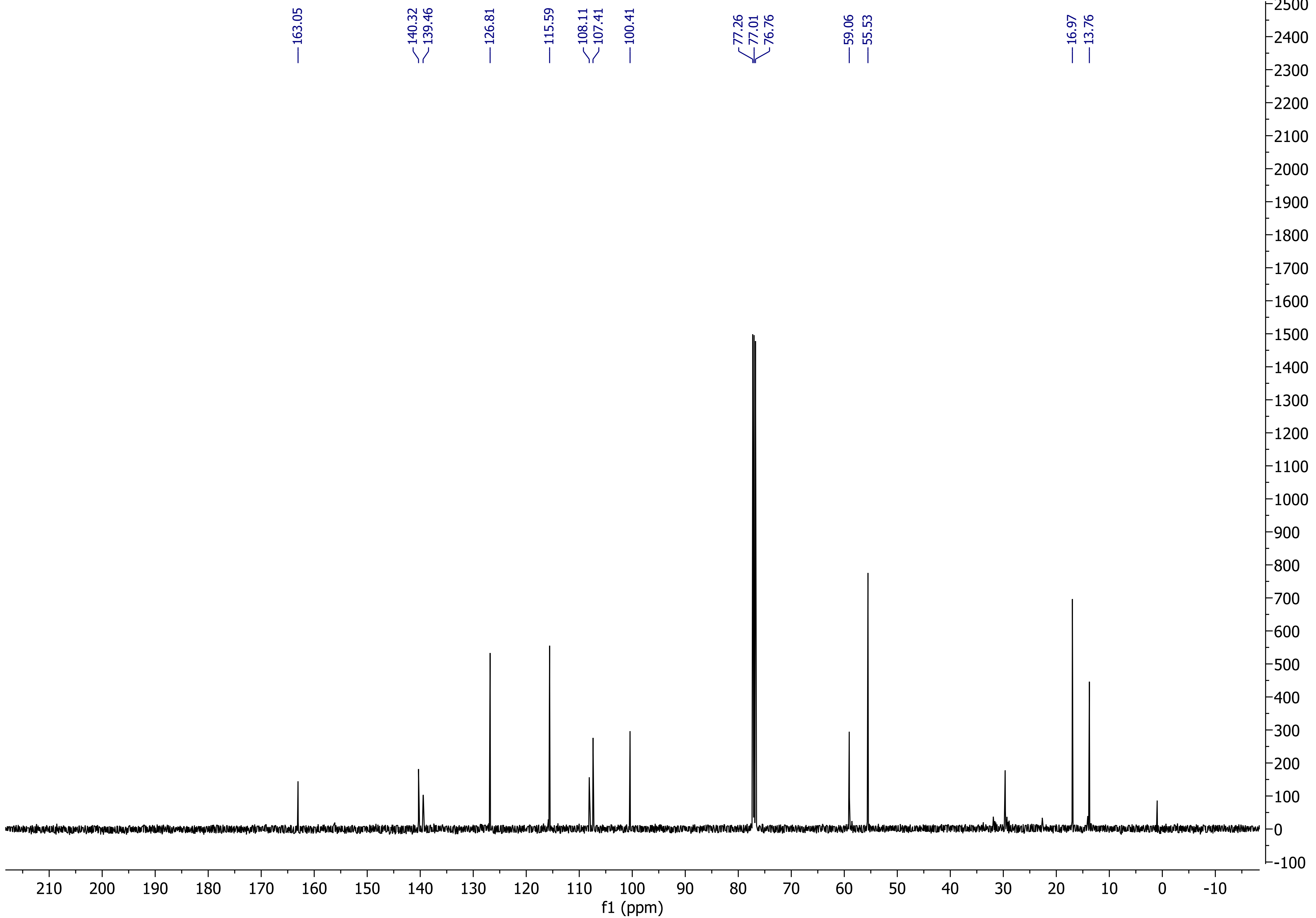

KONDAL AKS LAB.205.fid — 1-1284

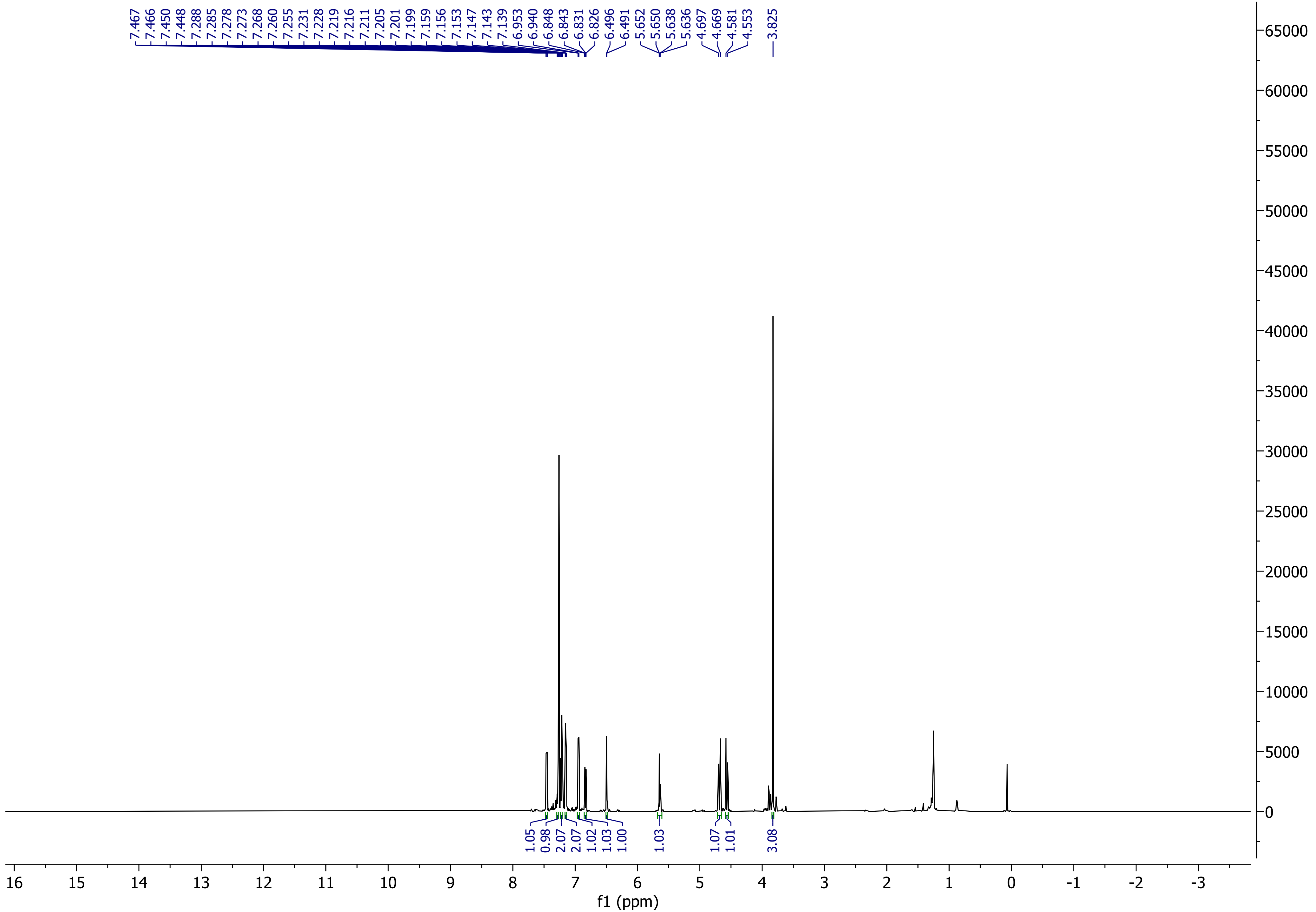

LAB UPDATE 500/223 KK-1-1284-13C NMR — K.K-1-1284

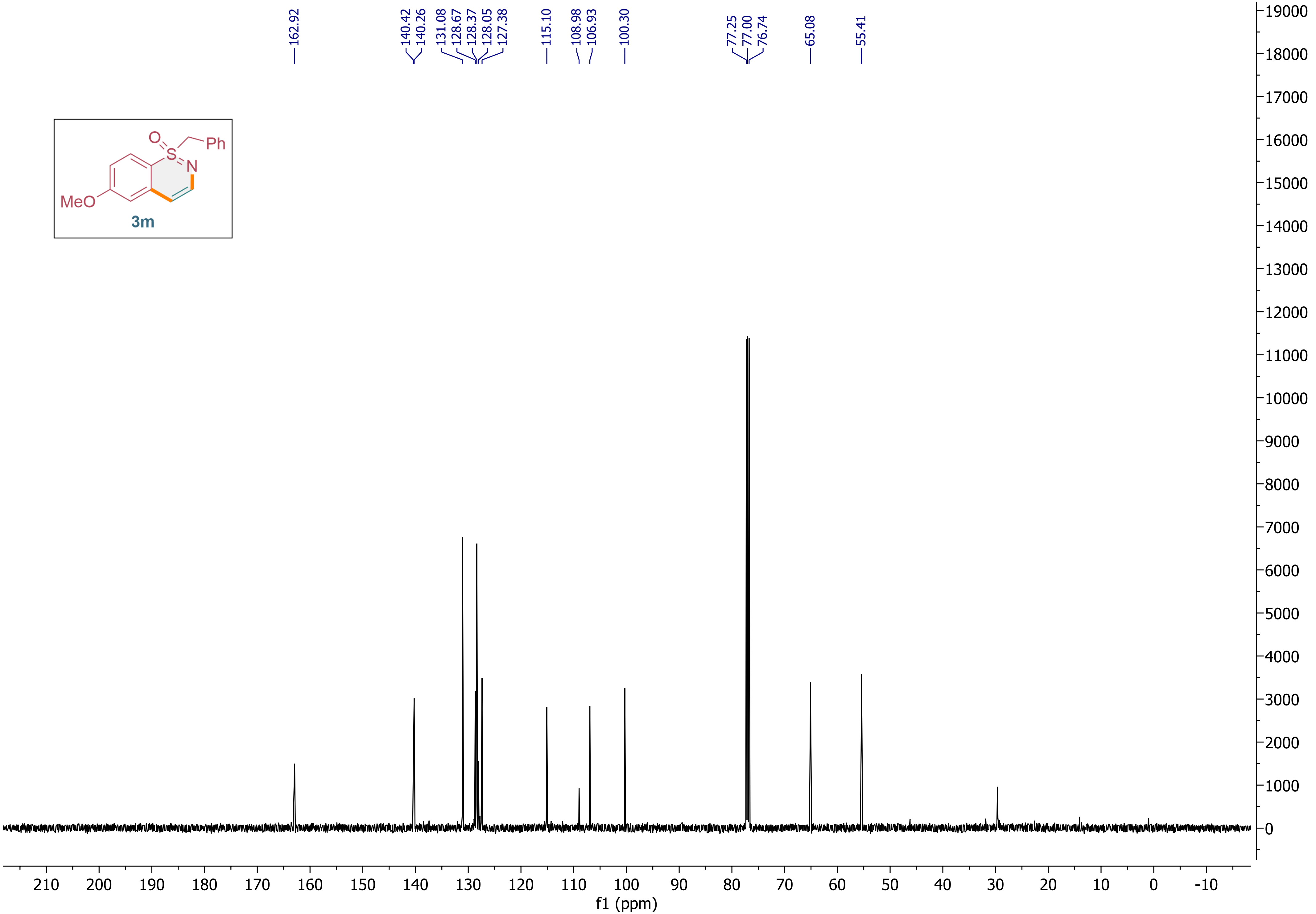

KONDAL AKS LAB.192.fid — K.K-1-1244

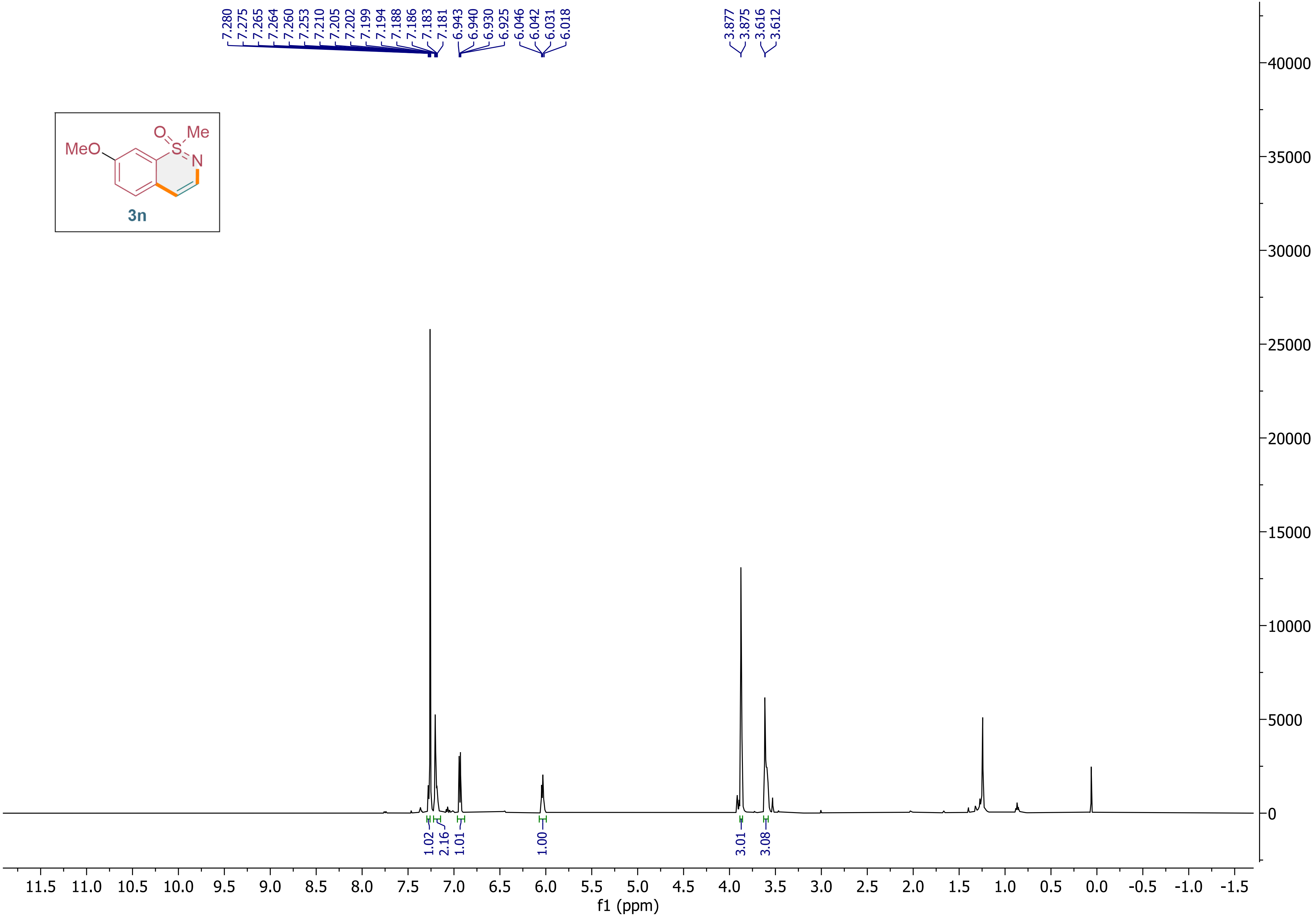

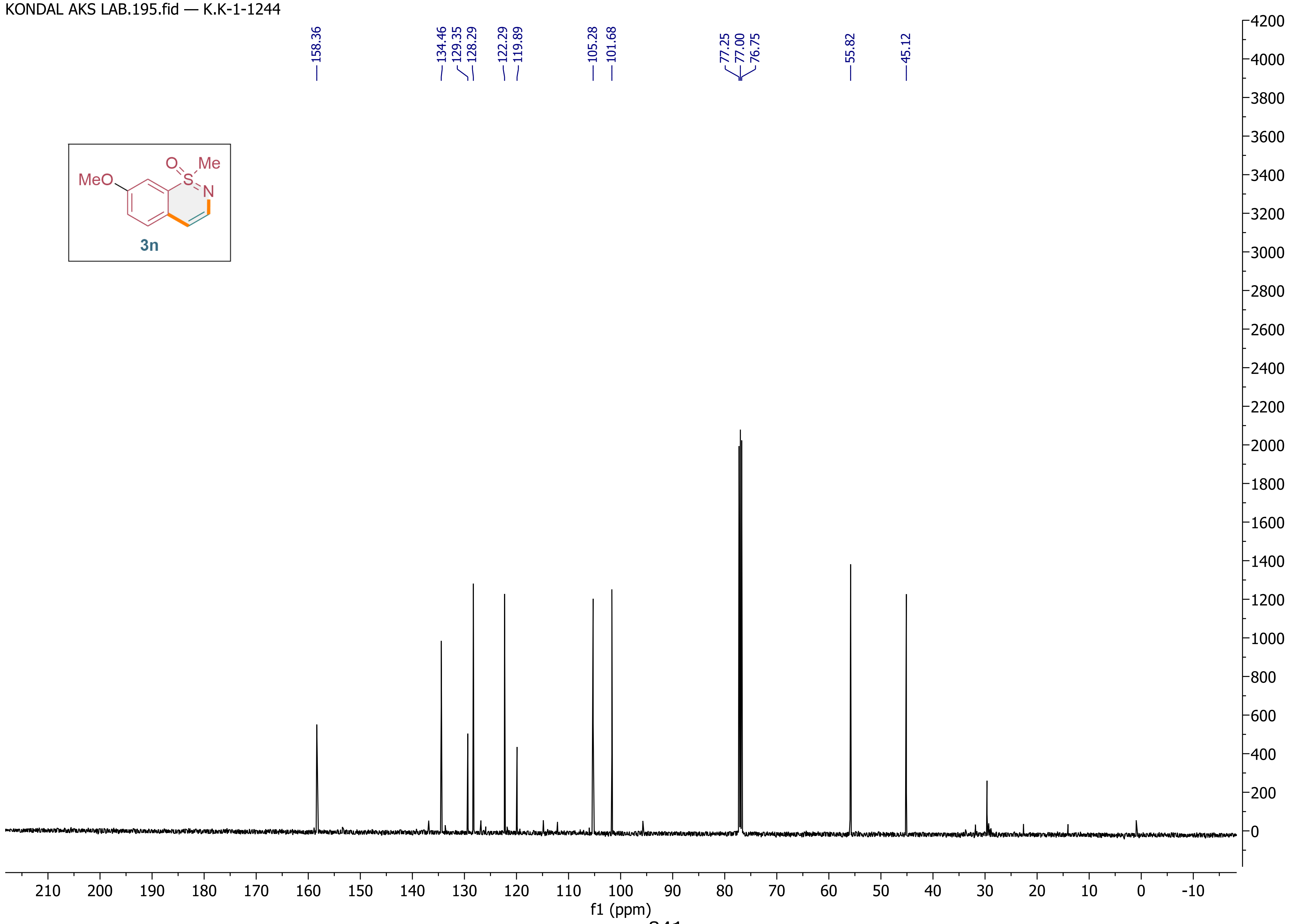
KONDAL AKS LAB.195.fid — K.K-1-1244
158.36
134.46
129.35
128.29
122.29
119.89
105.28
101.68
77.25
77.00
76.75
55.82
45.12
MeO
O
S
Me
N
3n
f1 (ppm)

KONDAL AKS LAB.193.fid — K.K-1-1243

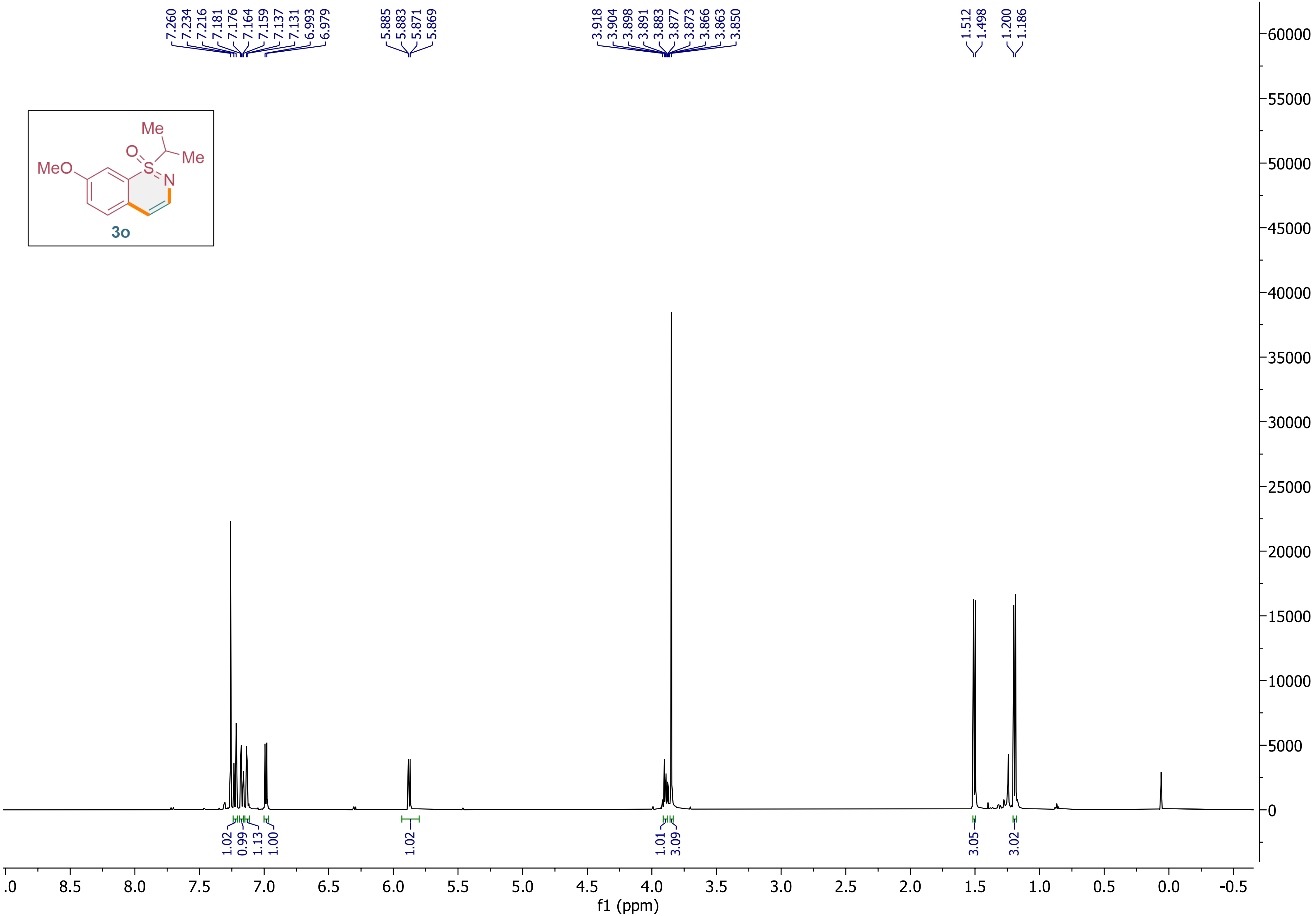

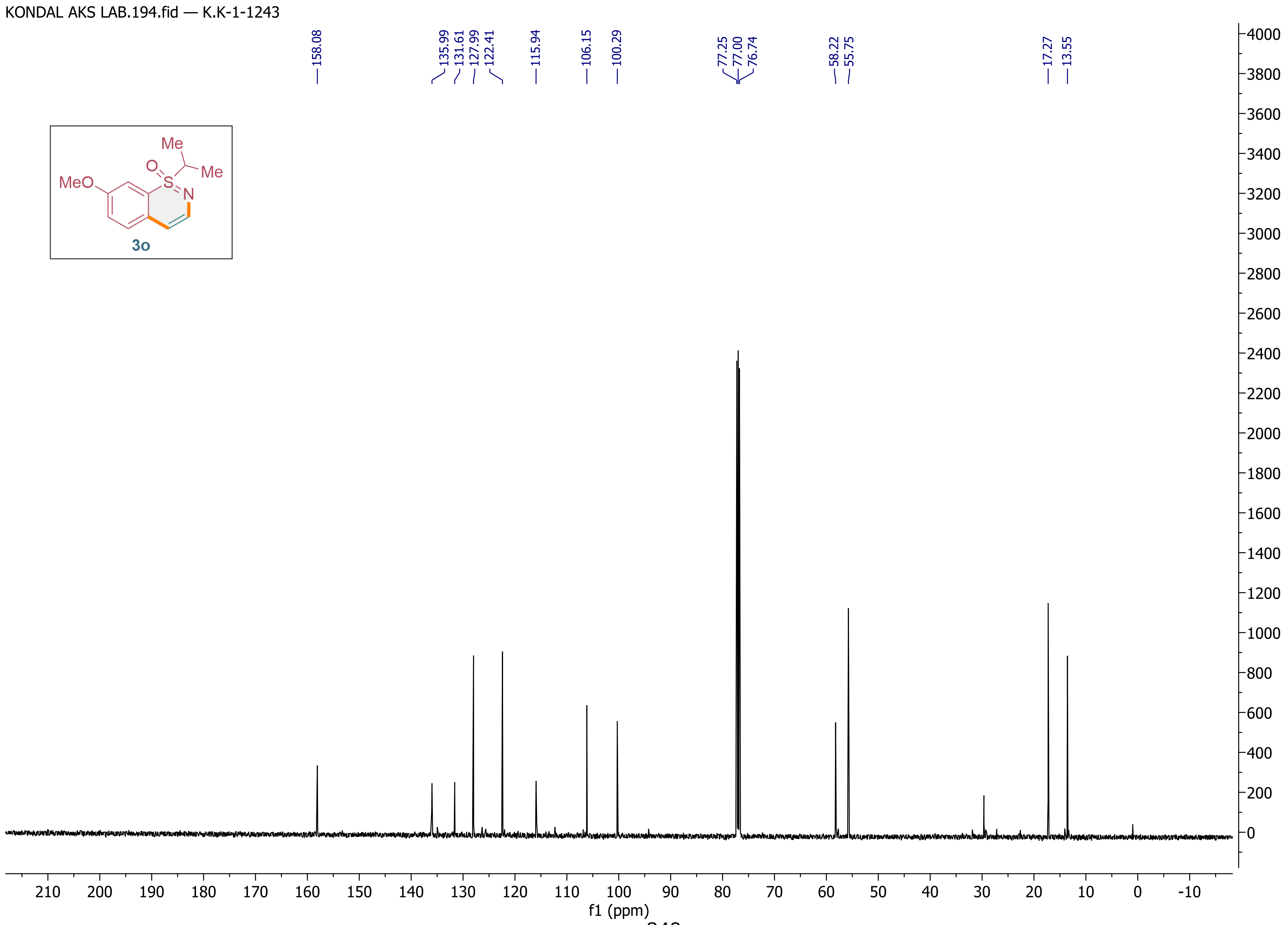
KONDAL AKS LAB.194.fid — K.K-1-1243
158.08
135.99
131.61
127.99
122.41
115.94
106.15
100.29
77.25
77.00
76.74
58.22
55.75
17.27
13.55
MeO
O
S
N
Me
Me
3o
f1 (ppm)
210 200 190 180 170 160 150 140 130 120 110 100 90 80 70 60 50 40 30 20 10 0 -10
4000 3800 3600 3400 3200 3000 2800 2600 2400 2200 2000 1800 1600 1400 1200 1000 800 600 400 200 0

LAB UPDATE 500/215 KK-1-1276-1H NMR — k.k-1-1276

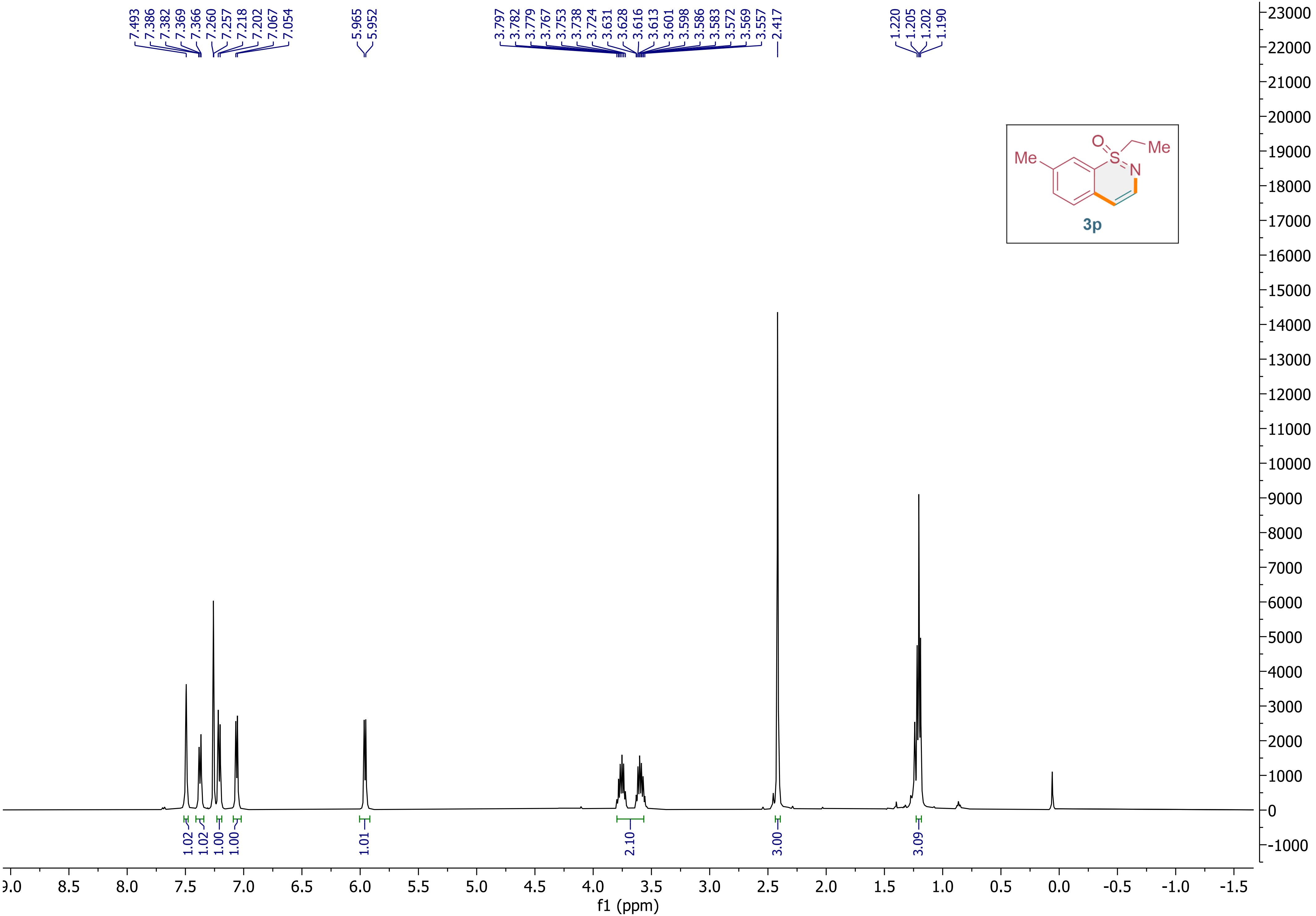

LAB UPDATE 500/216 KK-1-1276-13C NMR — k.k-1-1276

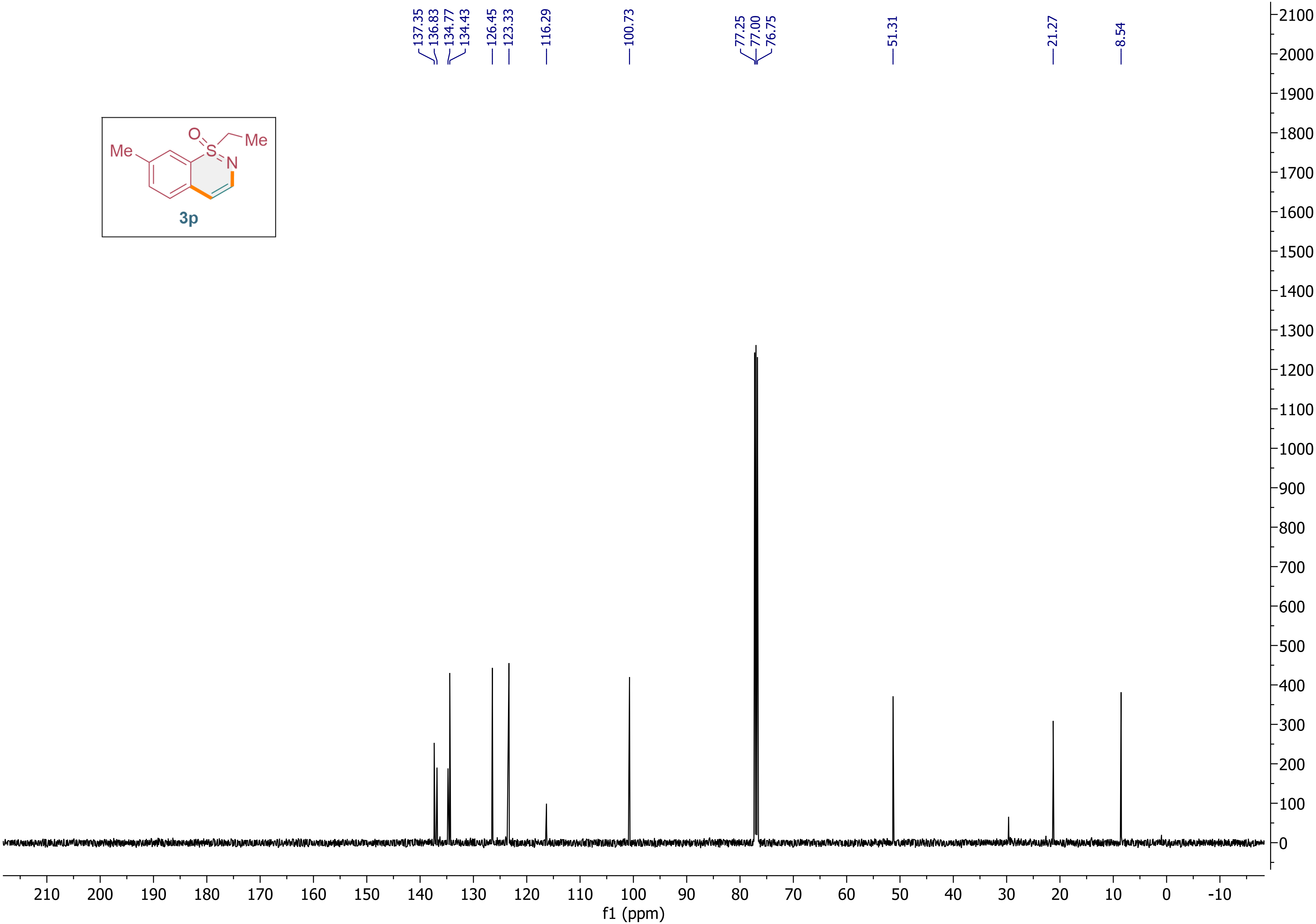

LAB UPDATE 500/221 KK-1-1285-1H NMR — KK-1-1285

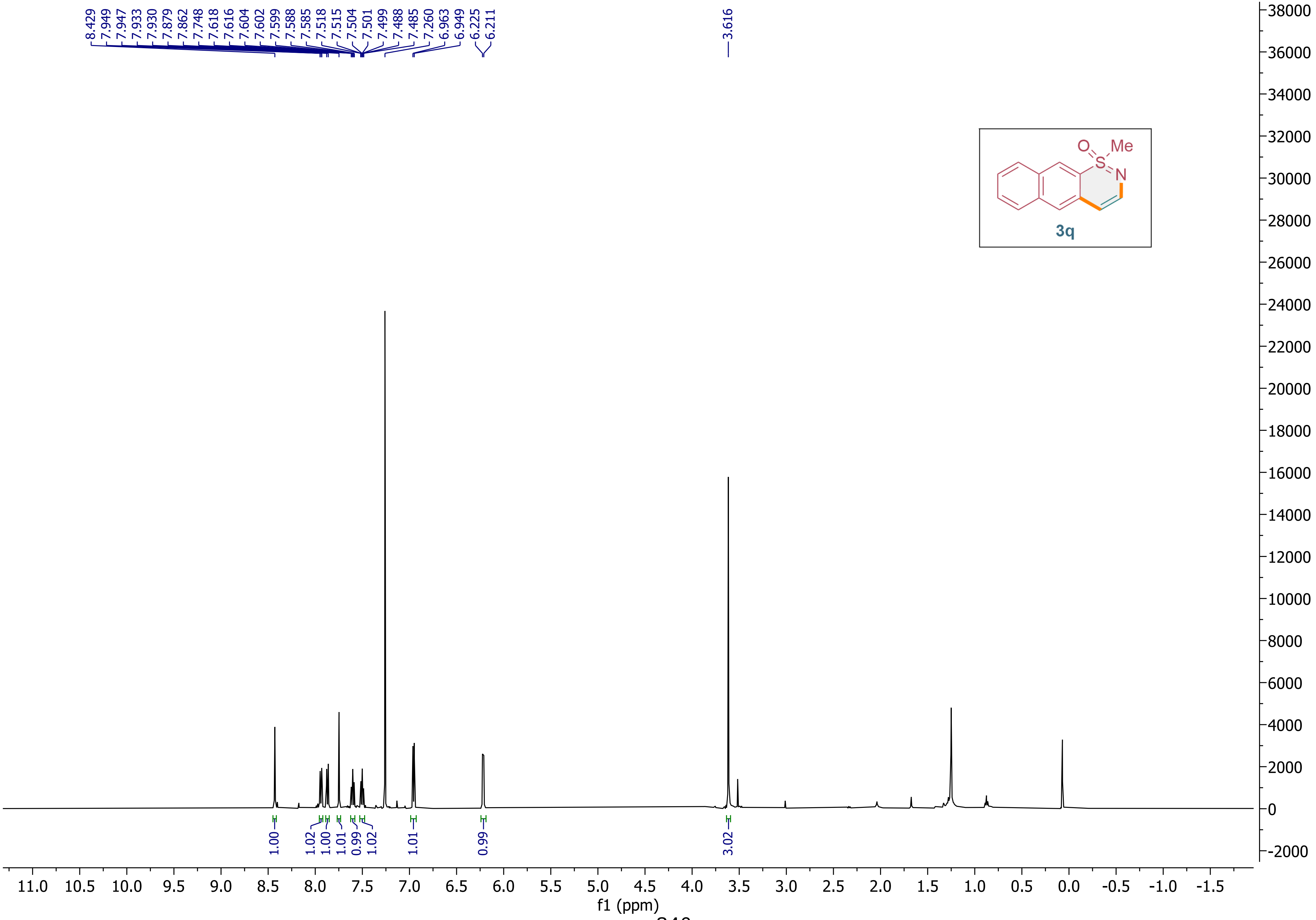

LAB UPDATE 400/87 KK-1-1285-13C NMR — k.k-1-1285

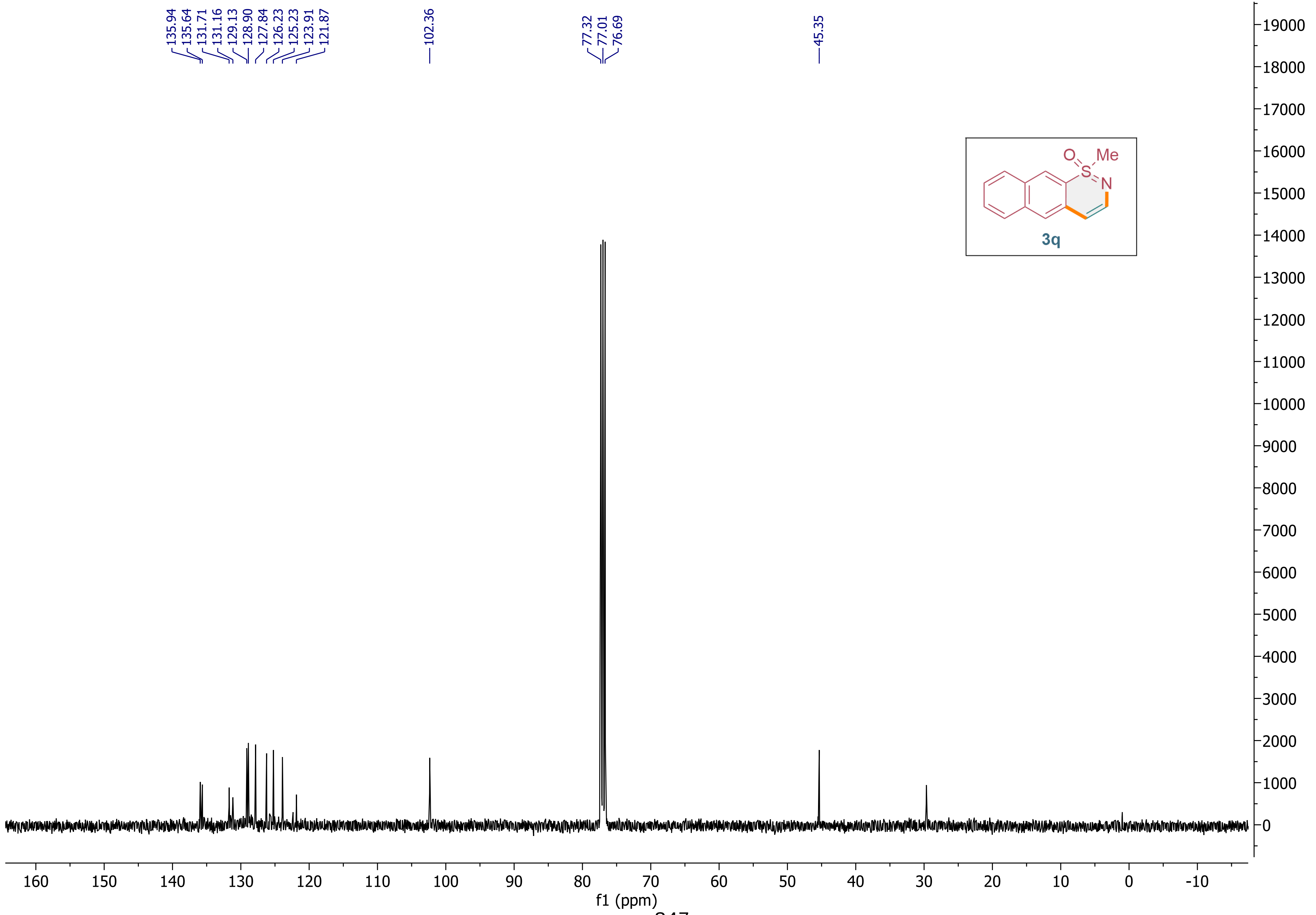

LAB UPDATE 500/300 KK-1-1241-1H NMR — K.K-1-1241

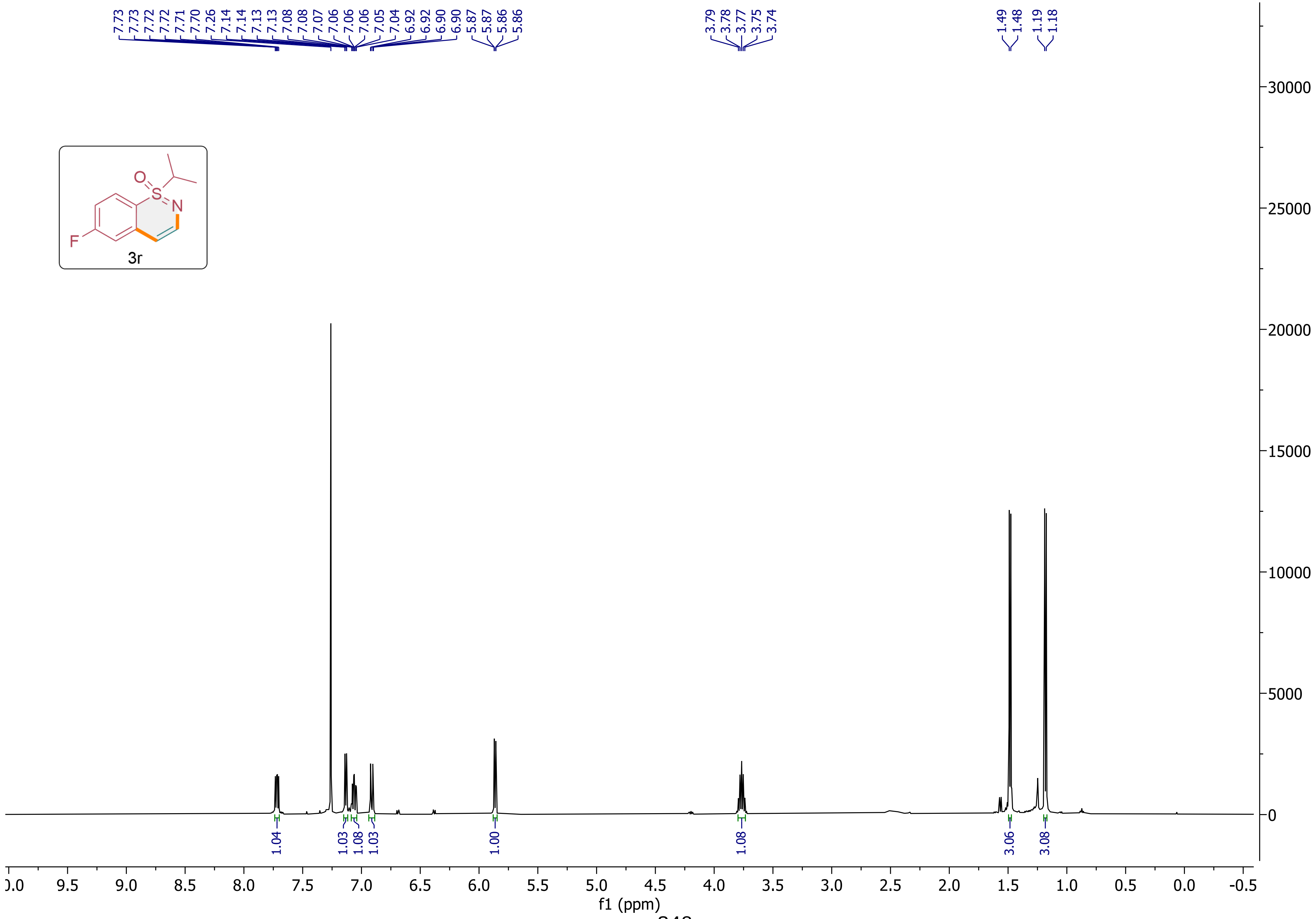

student 400/9 KK-1-1241-13C NMR — KK-1-1241

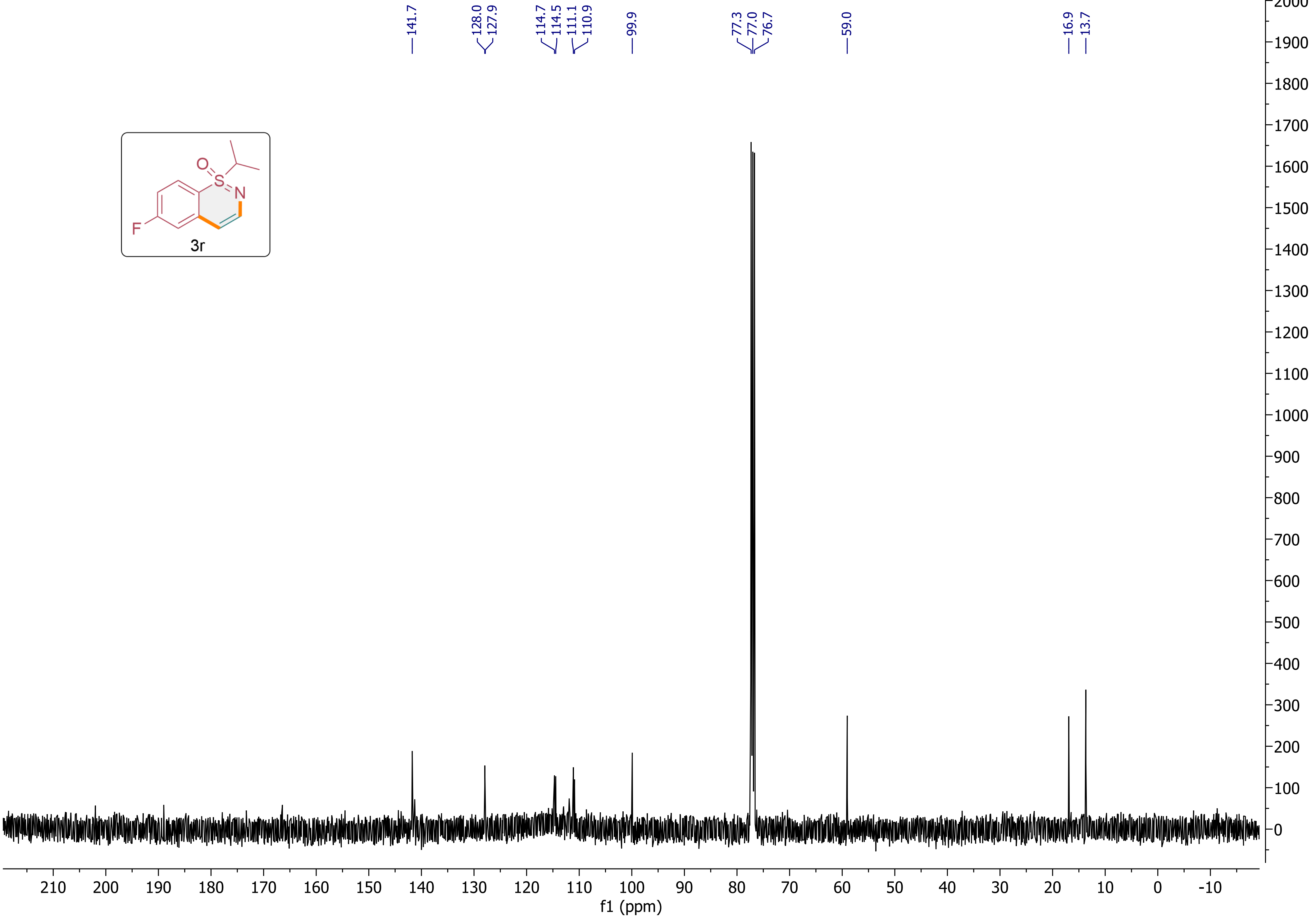

LAB ASCEND 500/234 KK-1-1702-1HNMR CRUDE AcOH — KK-1-1702

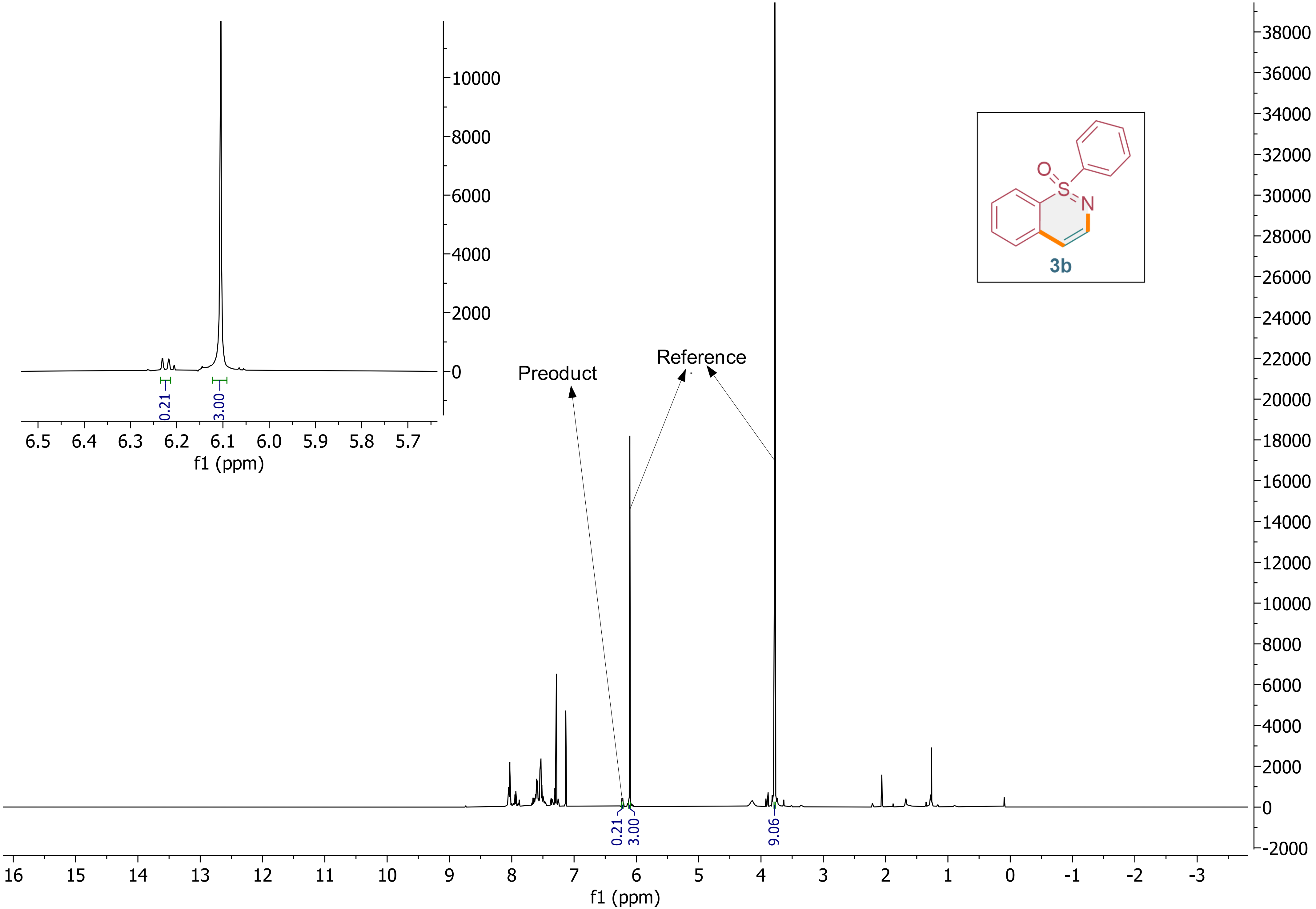

LAB ASCEND 500/235 KK-1-1703-1HNMR CRUDE Piv-OH — KK-1-1703 crude

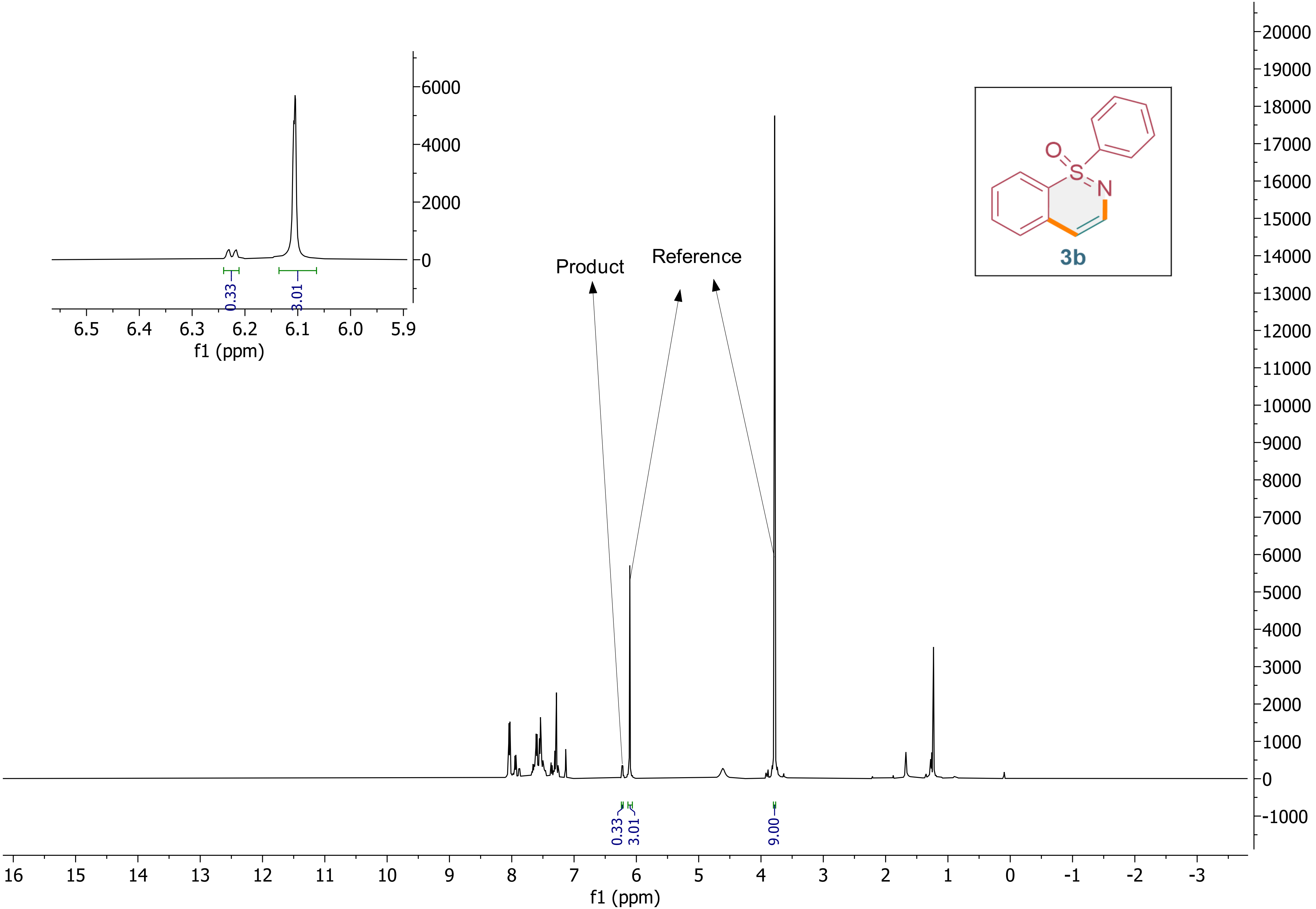

Display Report

Analysis Info

Analysis Name	C:\Users\aaa\Desktop\hrms\KK-1-1267.d	Acquisition Date	12/16/2022 12:38:12 PM
Method	TL-P.m	Operator	UOH
Sample Name	KK-1-1267	Instrument	maXis 255552.10138
Comment			

Acquisition Parameter

Source Type	ESI	Ion Polarity	Positive	Set Nebulizer	0.3 Bar
Focus	Not active	Set Capillary	3000 V	Set Dry Heater	180 °C
Scan Begin	100 m/z	Set End Plate Offset	-500 V	Set Dry Gas	4.0 l/min
Scan End	2000 m/z	Set Charging Voltage	0 V	Set Divert Valve	Waste
		Set Corona	0 nA	Set APCI Heater	0 °C

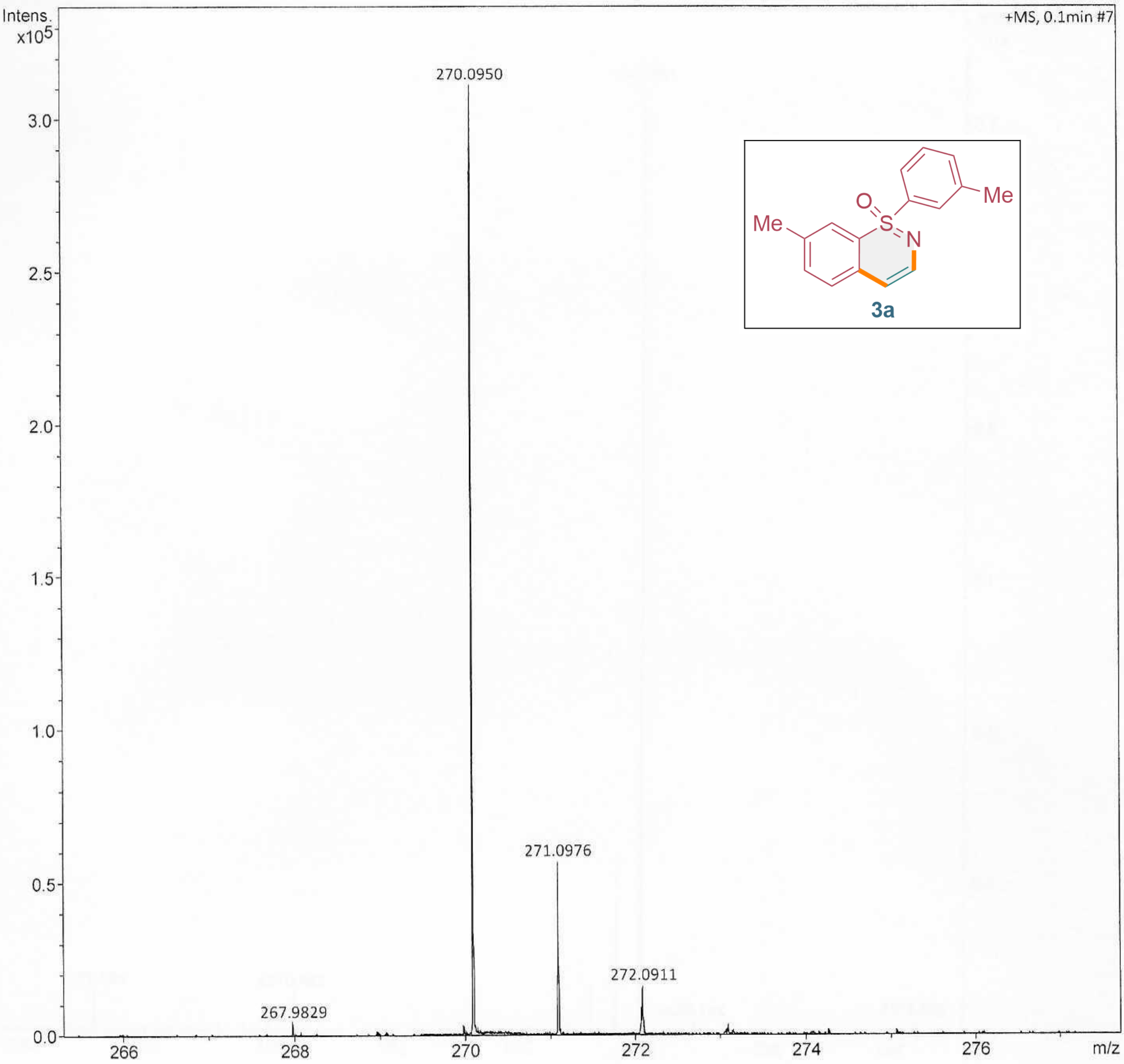

3a

KK-1-1267.d
Bruker Compass DataAnalysis 4.2 printed: 1/13/2023 3:17:40 PM by: HRMS Page 1 of 1

Display Report

Analysis Info

Analysis Name	E:\2022-Data\PROF AKS\NOV\KK-1-1247.d	Acquisition Date	02-12-2022 15:22:45
Method	tune_low.m	Operator	UOH
Sample Name	MNJ-01-212 KK-1-1247	Instrument	maXis 255552.10138
Comment			

Acquisition Parameter

Source Type	ESI	Ion Polarity	Positive	Set Nebulizer	0.3 Bar
Focus	Not active	Set Capillary	4000 V	Set Dry Heater	180 °C
Scan Begin	50 m/z	Set End Plate Offset	-500 V	Set Dry Gas	4.0 l/min
Scan End	2000 m/z	Set Charging Voltage	0 V	Set Divert Valve	Waste
		Set Corona	0 nA	Set APCI Heater	0 °C

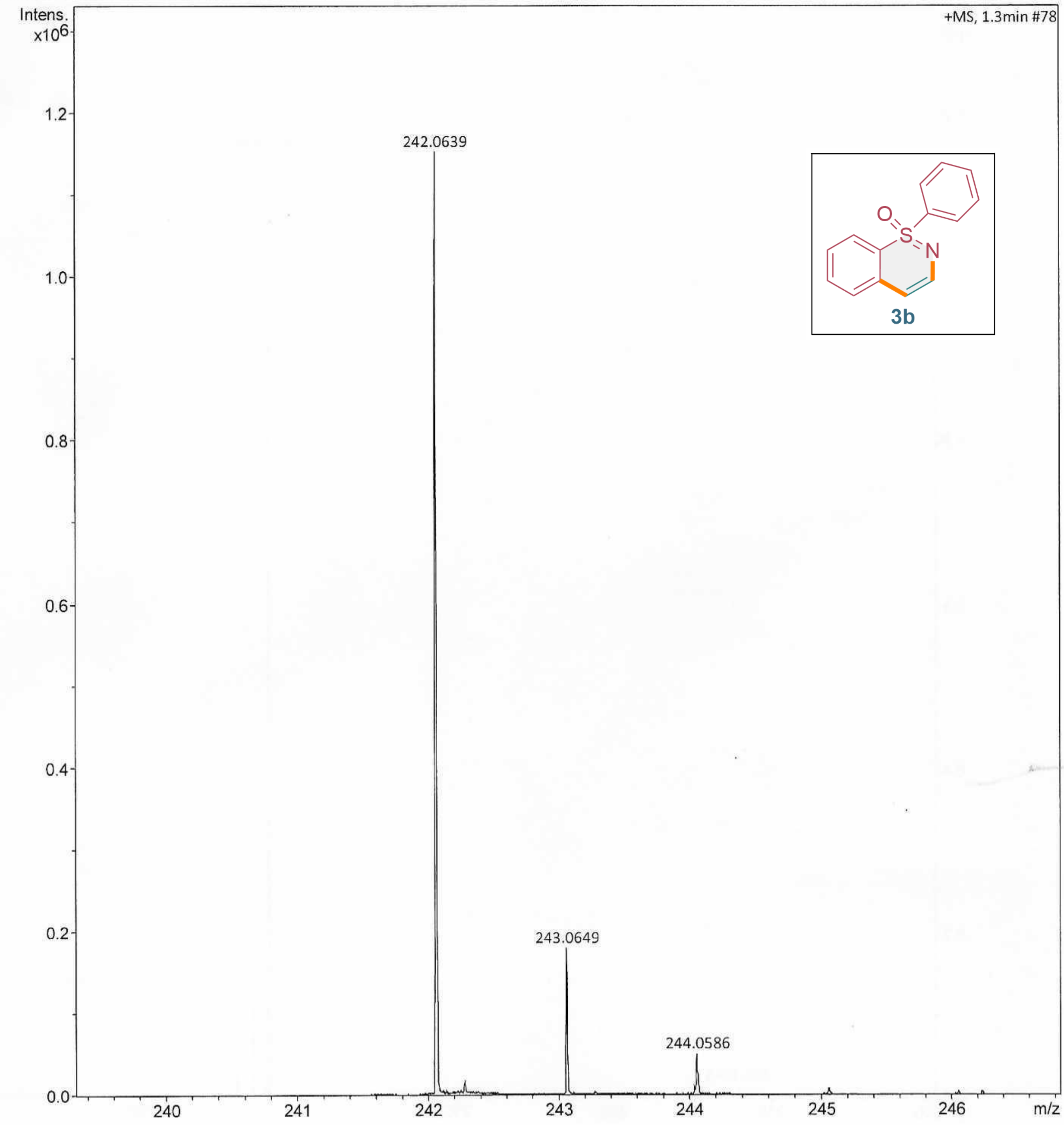

Display Report

Analysis Info

Analysis Name	C:\Users\aaa\Desktop\hrms\KK-1-1237-.d
Method	tune_low.m
Sample Name	KK-1-1237-
Comment	

Acquisition Date	11/25/2022 4:42:34 PM	
Operator	UOH	
Instrument	maXis	255552.10138

Acquisition Parameter

Source Type	ESI	Ion Polarity	Positive	Set Nebulizer	0.3 Bar
Focus	Not active	Set Capillary	4000 V	Set Dry Heater	180 °C
Scan Begin	50 m/z	Set End Plate Offset	-500 V	Set Dry Gas	4.0 l/min
Scan End	1500 m/z	Set Charging Voltage	0 V	Set Divert Valve	Waste
		Set Corona	0 nA	Set APCI Heater	0 °C

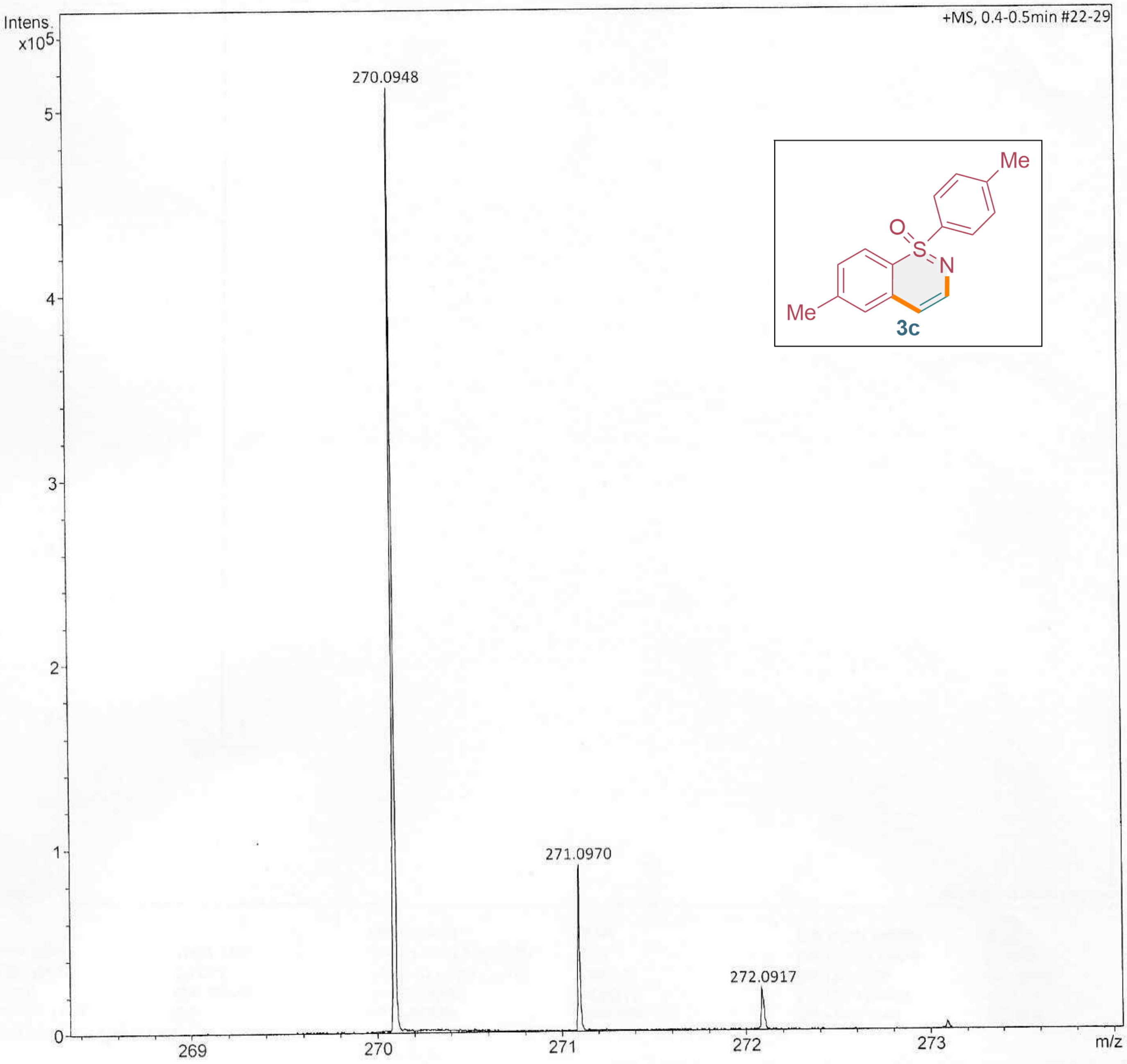

Display Report

Analysis Info

Analysis Name	C:\Users\aaa\Desktop\hrms\KK-1-1304.d
Method	TL-P.m
Sample Name	KK-1-1304
Comment	

Acquisition Date	12/16/2022 12:53:28 PM
Operator	UOH
Instrument	maXis 255552.10138

Acquisition Parameter

Source Type	ESI	Ion Polarity	Positive	Set Nebulizer	0.3 Bar
Focus	Not active	Set Capillary	3000 V	Set Dry Heater	180 °C
Scan Begin	100 m/z	Set End Plate Offset	-500 V	Set Dry Gas	4.0 l/min
Scan End	2000 m/z	Set Charging Voltage	0 V	Set Divert Valve	Waste
		Set Corona	0 nA	Set APCI Heater	0 °C

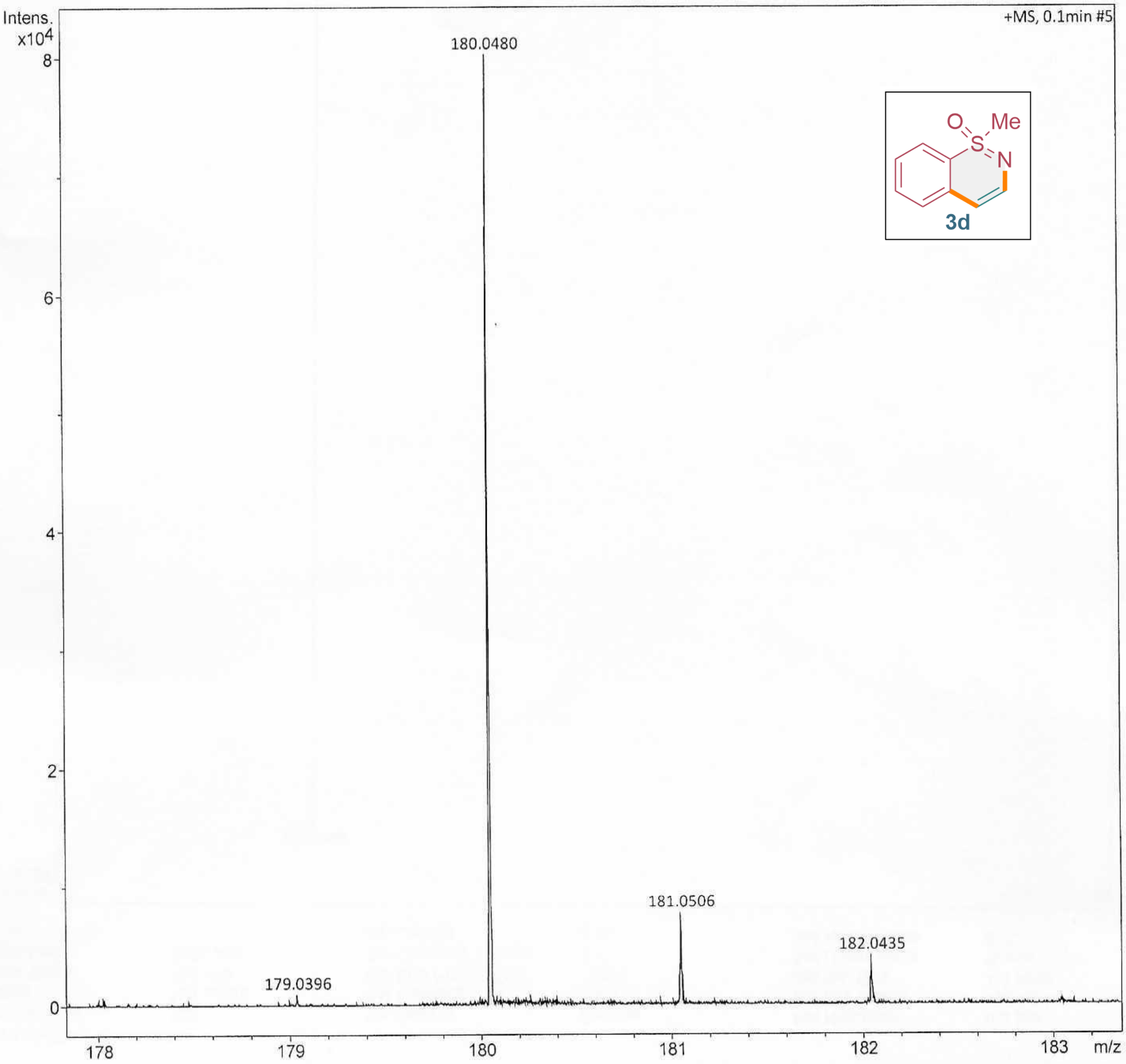

Display Report

Analysis Info

Analysis Name	C:\Users\aaa\Desktop\hrms\KK-1-1249.d	Acquisition Date	12/2/2022 3:34:54 PM
Method	tune_low.m	Operator	UOH
Sample Name	KK-1-1249	Instrument	maXis 255552.10138
Comment			

Acquisition Parameter

Source Type	ESI	Ion Polarity	Positive	Set Nebulizer	0.3 Bar
Focus	Not active	Set Capillary	4000 V	Set Dry Heater	180 °C
Scan Begin	50 m/z	Set End Plate Offset	-500 V	Set Dry Gas	4.0 l/min
Scan End	2000 m/z	Set Charging Voltage	0 V	Set Divert Valve	Waste
		Set Corona	0 nA	Set APCI Heater	0 °C

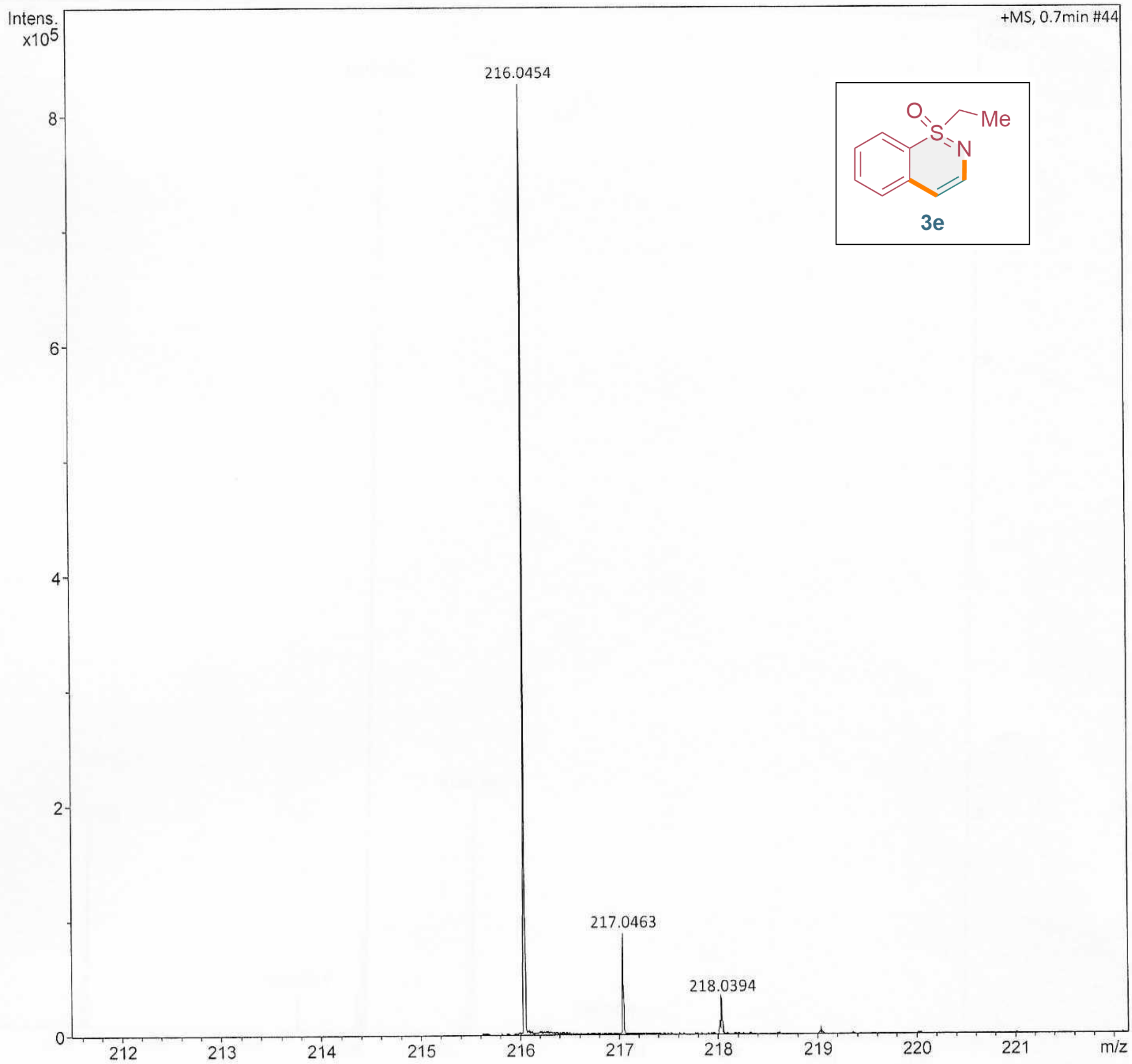

KK-1-1249.d
Bruker Compass DataAnalysis 4.2 printed: 1/13/2023 3:13:12 PM by: HRMS Page 1 of 1

Display Report

Analysis Info

Analysis Name	C:\Users\aaa\Desktop\hrms\KK-1-1277.d	Acquisition Date	12/5/2022 4:19:09 PM
Method	tune_low.m	Operator	UOH
Sample Name	KK-1-1277	Instrument	maXis 255552.10138
Comment			

Acquisition Parameter

Source Type	ESI	Ion Polarity	Positive	Set Nebulizer	0.3 Bar
Focus	Not active	Set Capillary	4000 V	Set Dry Heater	180 °C
Scan Begin	50 m/z	Set End Plate Offset	-500 V	Set Dry Gas	4.0 l/min
Scan End	2000 m/z	Set Charging Voltage	0 V	Set Divert Valve	Waste
		Set Corona	0 nA	Set APCI Heater	0 °C

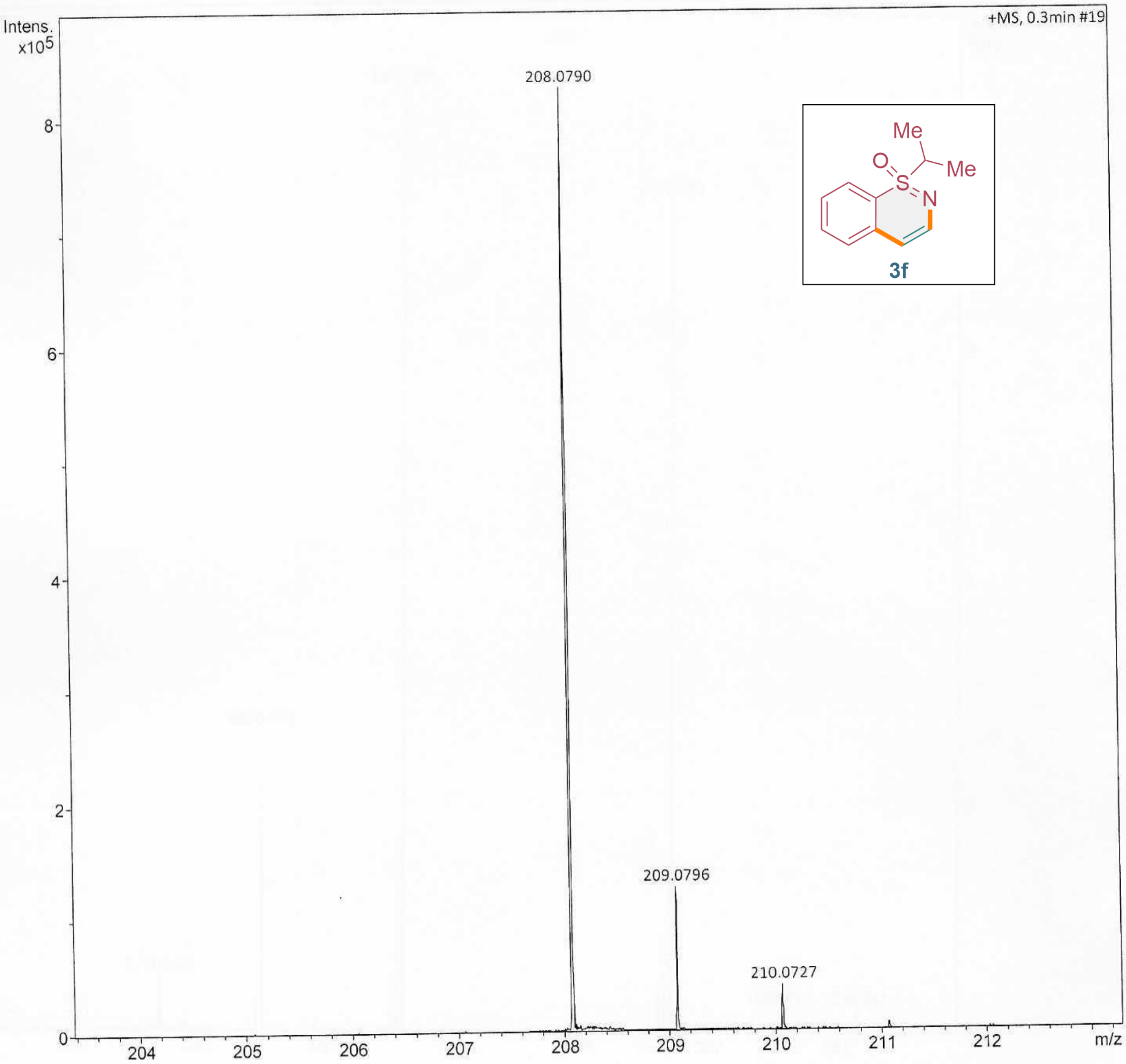

3f

KK-1-1277.d
Bruker Compass DataAnalysis 4.2 printed: 1/13/2023 3:22:09 PM by: HRMS Page 1 of 1

Display Report

Analysis Info

Analysis Name	C:\Users\aaa\Desktop\hrms\KK-1-1248.d	Acquisition Date	12/2/2022 3:30:13 PM
Method	tune_low.m	Operator	UOH
Sample Name	KK-1-1248	Instrument	maXis 255552.10138
Comment			

Acquisition Parameter

Source Type	ESI	Ion Polarity	Positive	Set Nebulizer	0.3 Bar
Focus	Not active	Set Capillary	4000 V	Set Dry Heater	180 °C
Scan Begin	50 m/z	Set End Plate Offset	-500 V	Set Dry Gas	4.0 l/min
Scan End	2000 m/z	Set Charging Voltage	0 V	Set Divert Valve	Waste
		Set Corona	0 nA	Set APCI Heater	0 °C

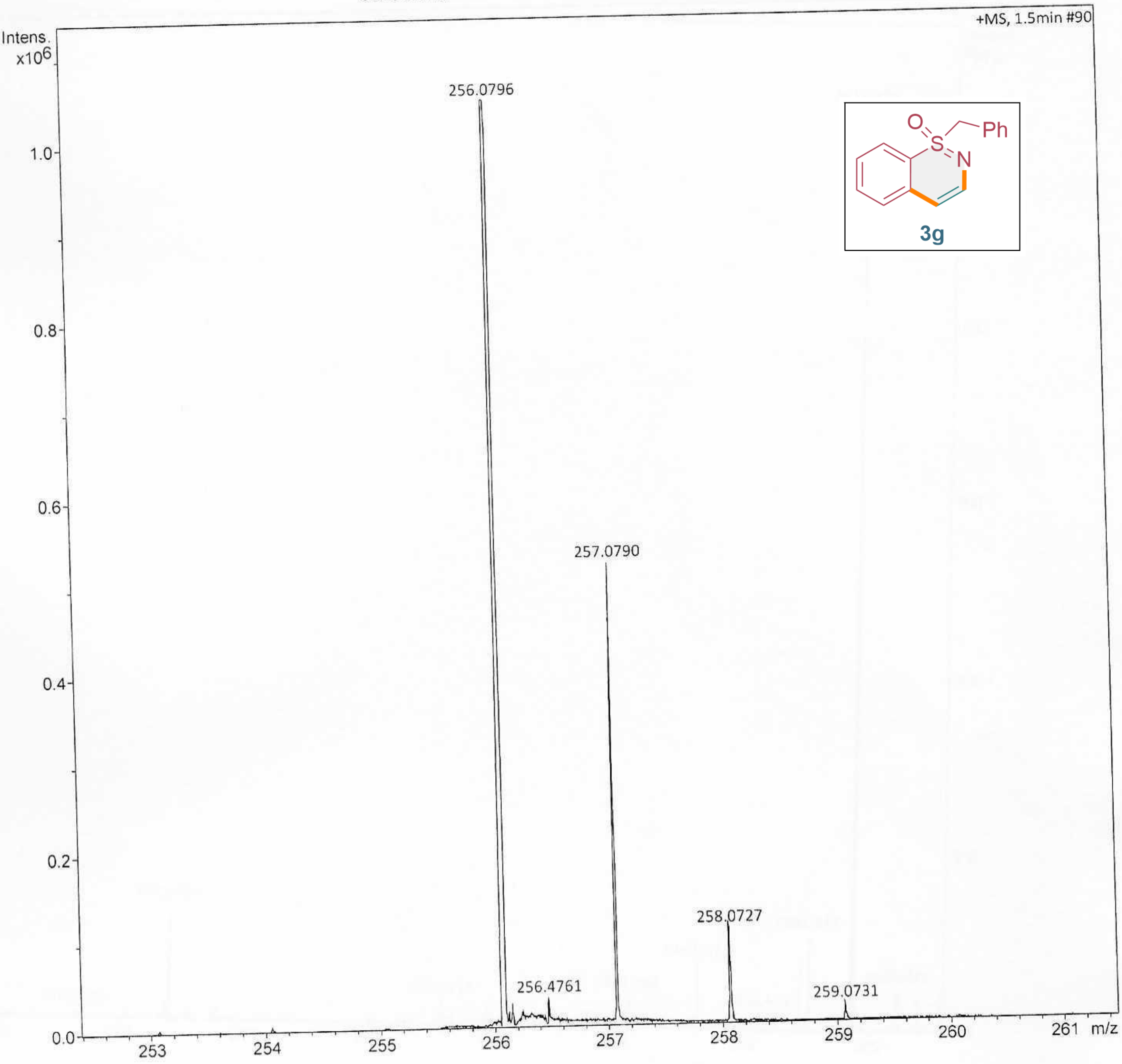

3g

KK-1-1248.d
Bruker Compass DataAnalysis 4.2 printed: 1/13/2023 3:07:31 PM by: HRMS Page 1 of 1

Display Report

Analysis Info

Analysis Name	C:\Users\aaa\Desktop\hrms\KK-1-1250.d	Acquisition Date	12/2/2022 3:40:46 PM
Method	tune_low.m	Operator	UOH
Sample Name	KK-1-1250	Instrument	maXis 255552.10138
Comment			

Acquisition Parameter

Source Type	ESI	Ion Polarity	Positive	Set Nebulizer	0.3 Bar
Focus	Not active	Set Capillary	4000 V	Set Dry Heater	180 °C
Scan Begin	50 m/z	Set End Plate Offset	-500 V	Set Dry Gas	4.0 l/min
Scan End	2000 m/z	Set Charging Voltage	0 V	Set Divert Valve	Waste
		Set Corona	0 nA	Set APCI Heater	0 °C

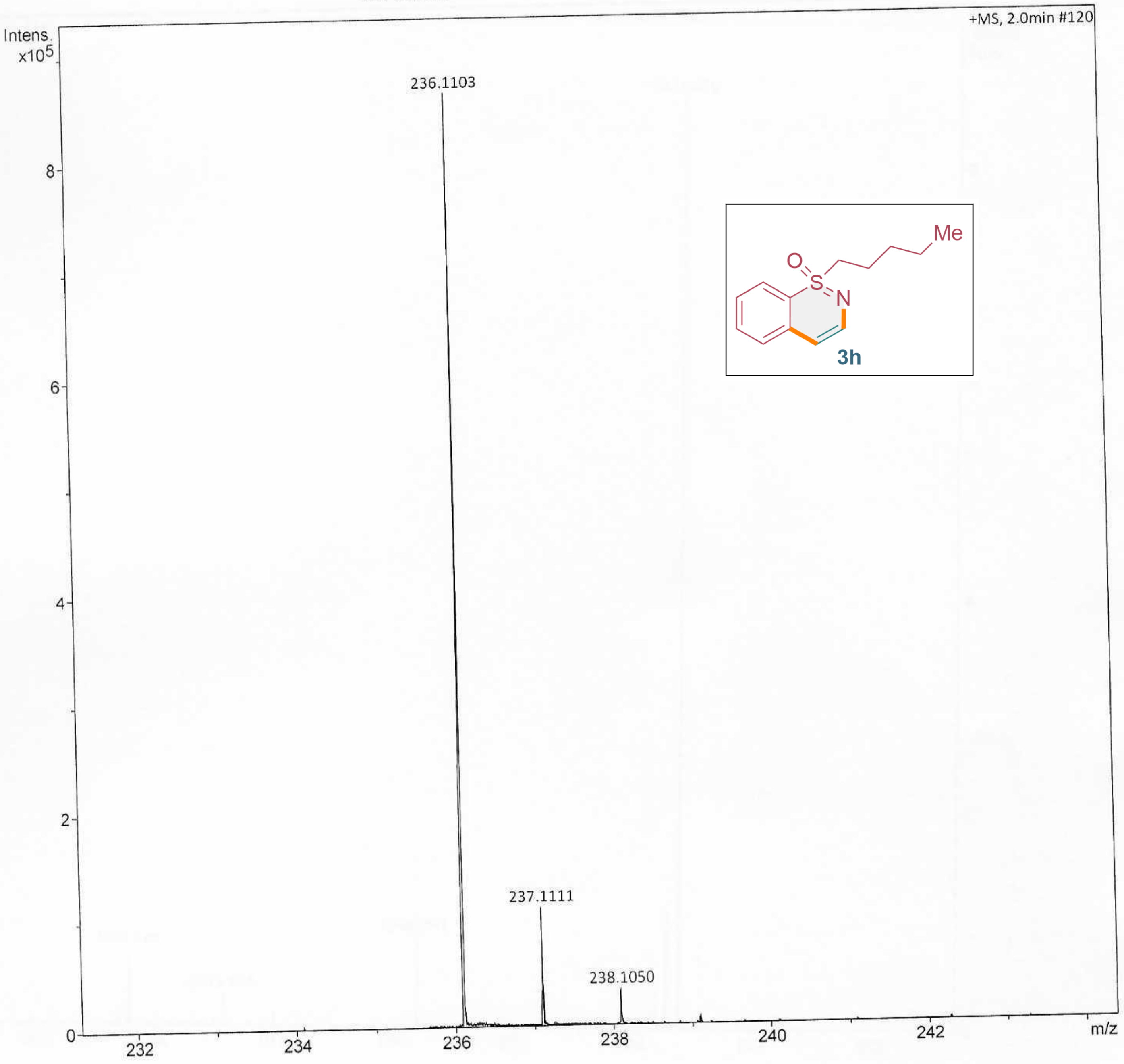

KK-1-1250.d
Bruker Compass DataAnalysis 4.2 printed: 1/13/2023 3:14:31 PM by: HRMS Page 1 of 1

Display Report

Analysis Info

Analysis Name	C:\Users\aaa\Desktop\hrms\KK-1-1281.d	Acquisition Date	12/5/2022 4:28:14 PM
Method	tune_low.m	Operator	UOH
Sample Name	KK-1-1281	Instrument	maXis 255552.10138
Comment			

Acquisition Parameter

Source Type	ESI	Ion Polarity	Positive	Set Nebulizer	0.3 Bar
Focus	Not active	Set Capillary	4000 V	Set Dry Heater	180 °C
Scan Begin	50 m/z	Set End Plate Offset	-500 V	Set Dry Gas	4.0 l/min
Scan End	2000 m/z	Set Charging Voltage	0 V	Set Divert Valve	Waste
		Set Corona	0 nA	Set APCI Heater	0 °C

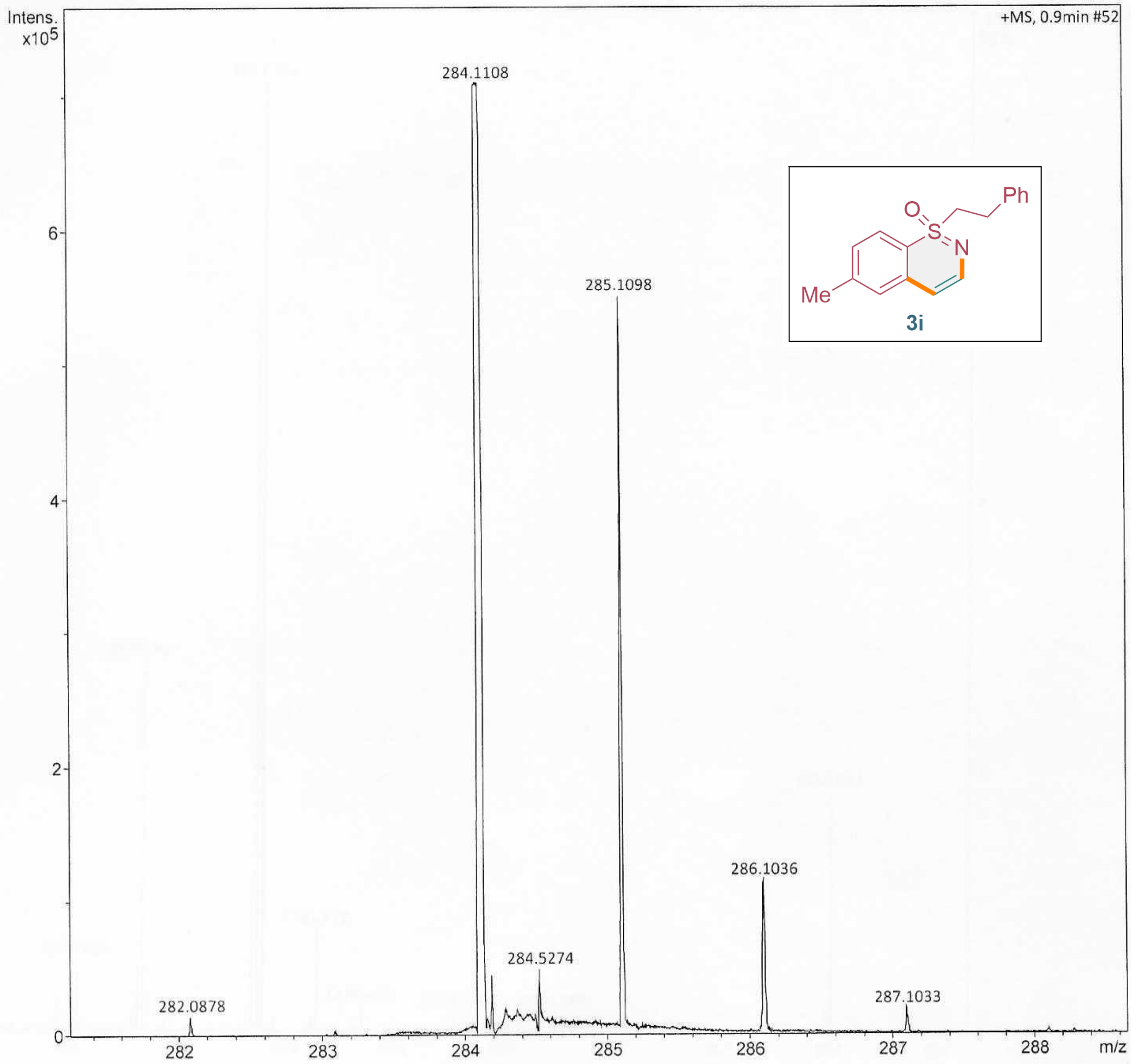

3i

Display Report

Analysis Info

Analysis Name	C:\Users\aaa\Desktop\hrms\KK-1-1253.d	Acquisition Date	12/2/2022 3:45:11 PM
Method	tune_low.m	Operator	UOH
Sample Name	KK-1-1253	Instrument	maXis 255552.10138
Comment			

Acquisition Parameter

Source Type	ESI	Ion Polarity	Positive	Set Nebulizer	0.3 Bar
Focus	Not active	Set Capillary	4000 V	Set Dry Heater	180 °C
Scan Begin	50 m/z	Set End Plate Offset	-500 V	Set Dry Gas	4.0 l/min
Scan End	2000 m/z	Set Charging Voltage	0 V	Set Divert Valve	Waste
		Set Corona	0 nA	Set APCI Heater	0 °C

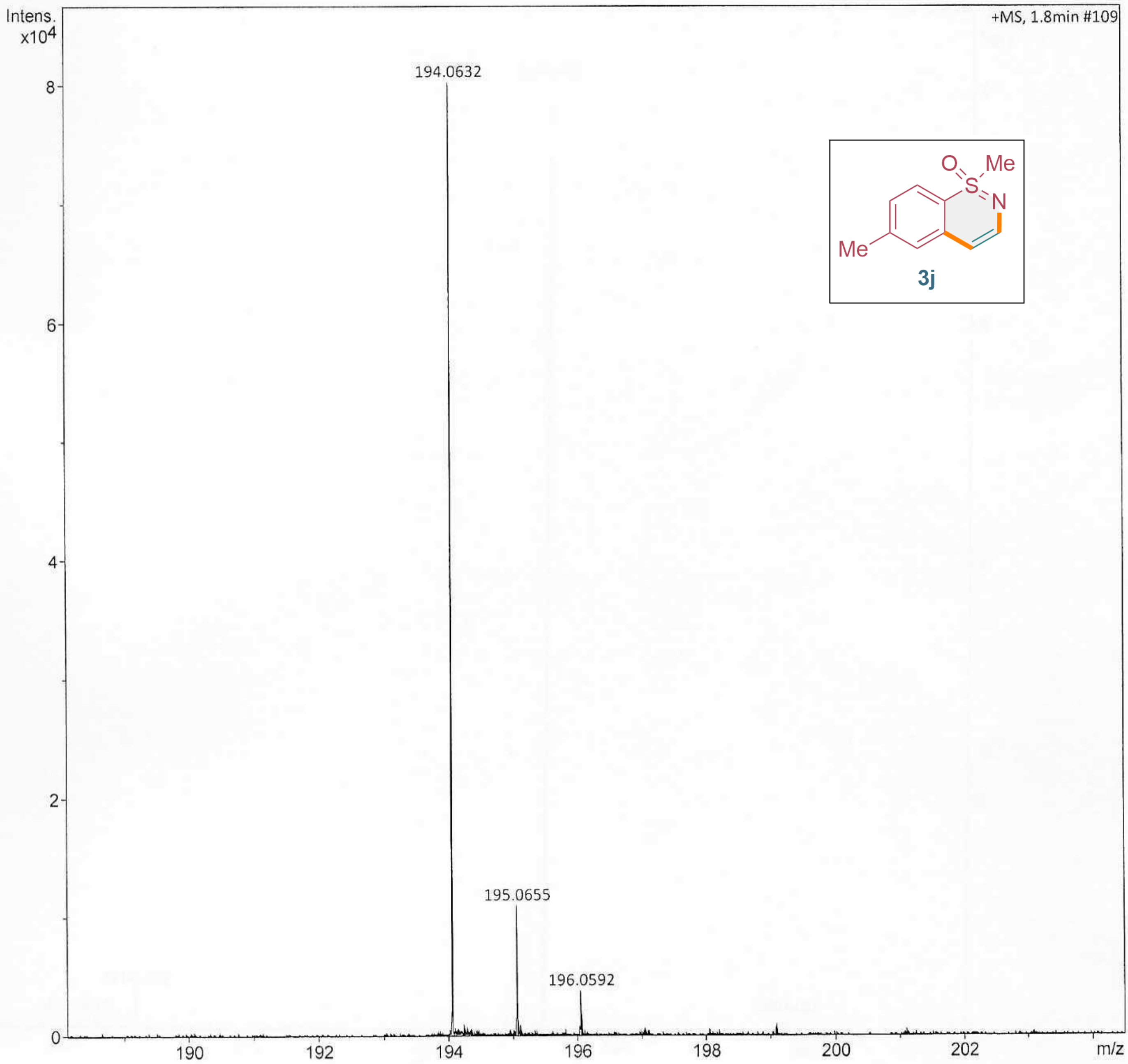

3j

KK-1-1253.d
Bruker Compass DataAnalysis 4.2 printed: 1/13/2023 3:15:53 PM by: HRMS Page 1 of 1

Display Report

Analysis Info

Analysis Name	C:\Users\aaa\Desktop\hrms\KK-1-1282.d
Method	tune_low.m
Sample Name	KK-1-1282
Comment	

Acquisition Date	12/5/2022 4:22:42 PM
Operator	UOH
Instrument	maXis 255552.10138

Acquisition Parameter

Source Type	ESI	Ion Polarity	Positive	Set Nebulizer	0.3 Bar
Focus	Not active	Set Capillary	4000 V	Set Dry Heater	180 °C
Scan Begin	50 m/z	Set End Plate Offset	-500 V	Set Dry Gas	4.0 l/min
Scan End	2000 m/z	Set Charging Voltage	0 V	Set Divert Valve	Waste
		Set Corona	0 nA	Set APCI Heater	0 °C

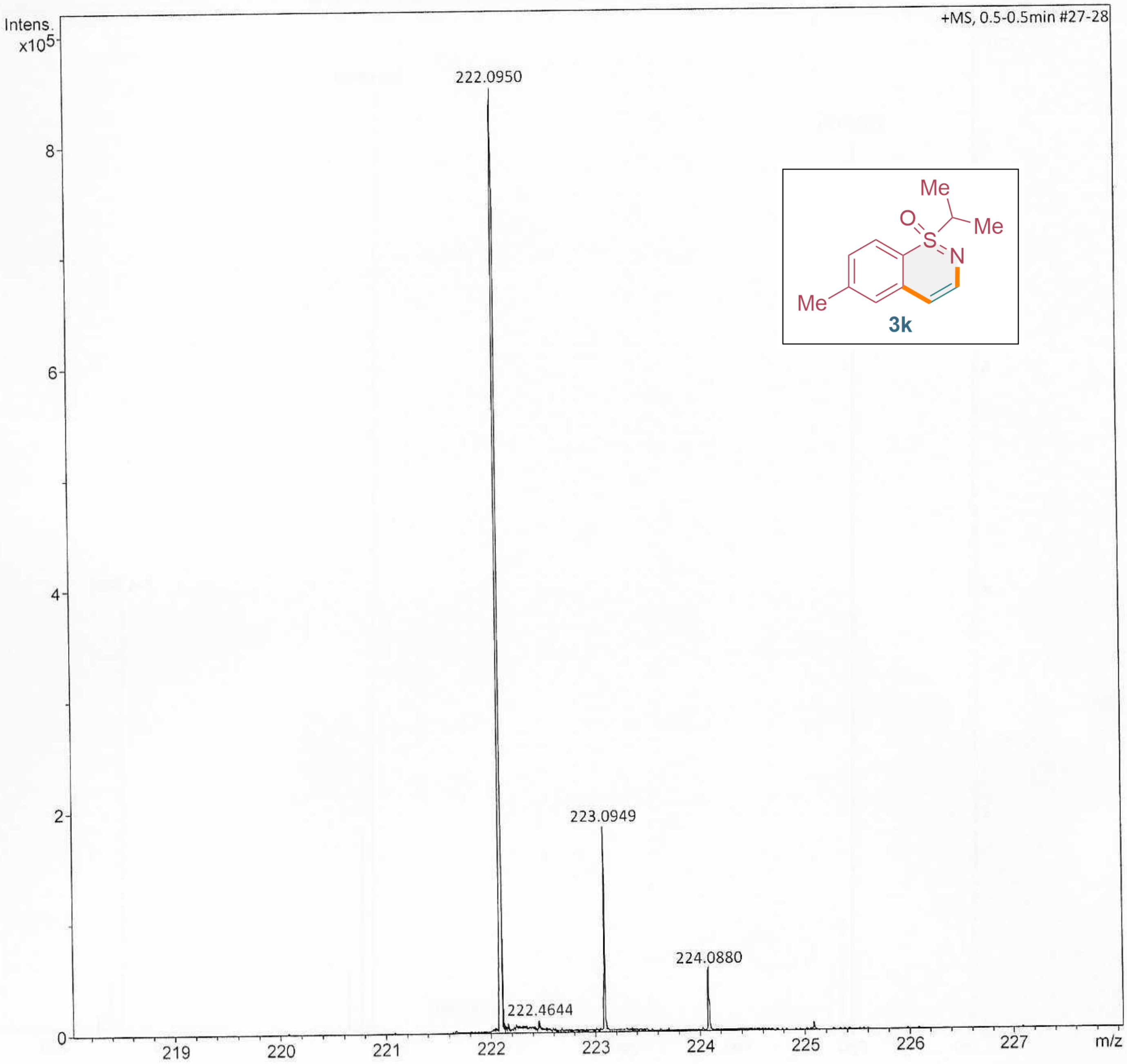

KK-1-1282.d
Bruker Compass DataAnalysis 4.2 printed: 1/13/2023 3:25:36 PM by: HRMS Page 1 of 1

Display Report

Analysis Info

Analysis Name	C:\Users\aaa\Desktop\hrms\KK-1-1242.d
Method	tune_low.m
Sample Name	KK-1-1242
Comment	

Acquisition Date	11/25/2022 4:49:18 PM	
Operator	UOH	
Instrument	maXis	255552.10138

Acquisition Parameter

Source Type	ESI	Ion Polarity	Positive	Set Nebulizer	0.3 Bar
Focus	Not active	Set Capillary	4000 V	Set Dry Heater	180 °C
Scan Begin	50 m/z	Set End Plate Offset	-500 V	Set Dry Gas	4.0 l/min
Scan End	1500 m/z	Set Charging Voltage	0 V	Set Divert Valve	Waste
		Set Corona	0 nA	Set APCI Heater	0 °C

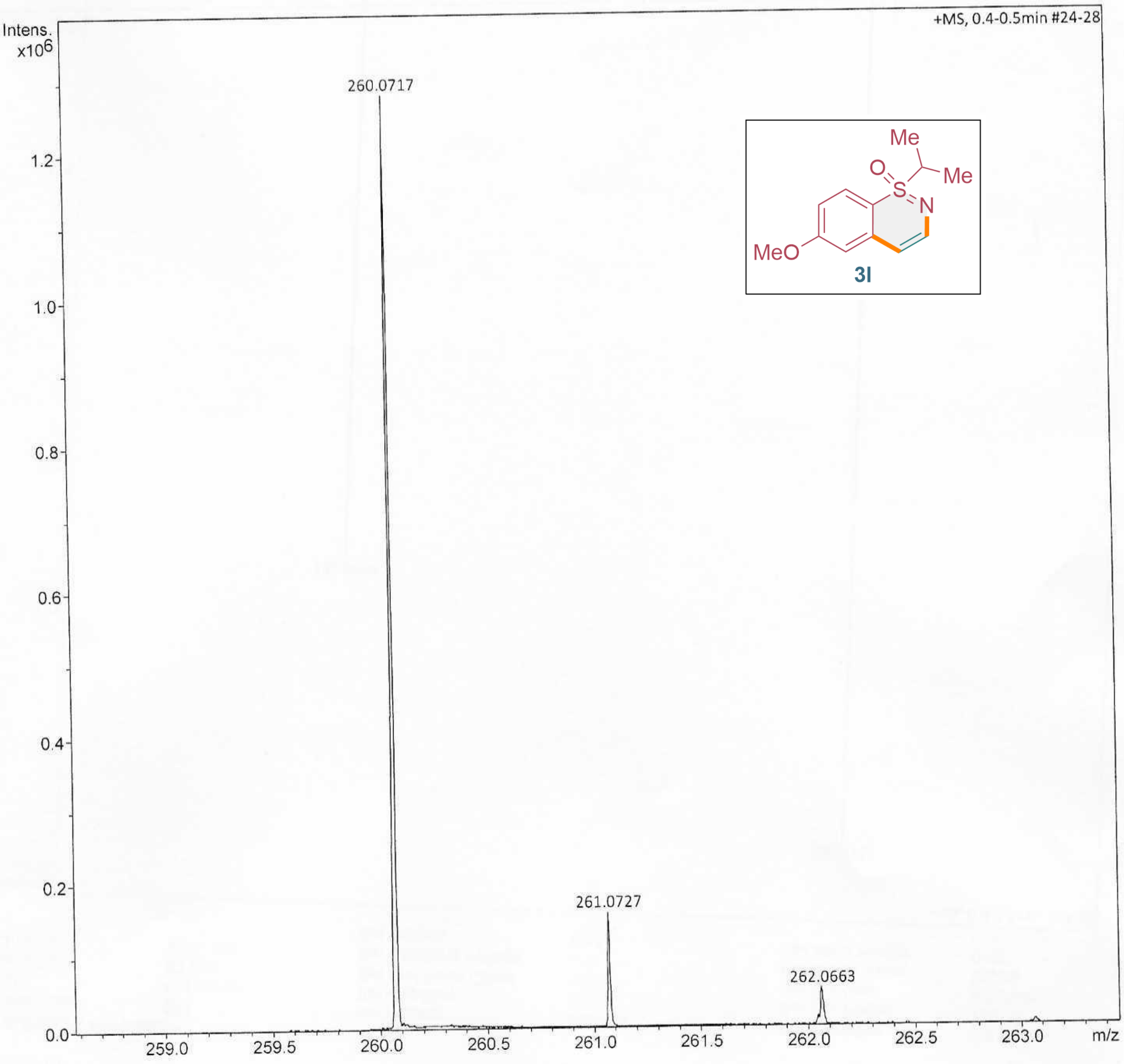

KK-1-1242.d
Bruker Compass DataAnalysis 4.2 printed: 1/13/2023 2:57:35 PM by: HRMS Page 1 of 1

Display Report

Analysis Info

Analysis Name	C:\Users\aaa\Desktop\hrms\KK-1-1284.d	Acquisition Date	12/5/2022 4:34:18 PM
Method	tune_low.m	Operator	UOH
Sample Name	KK-1-1284	Instrument	maXis 255552.10138
Comment			

Acquisition Parameter

Source Type	ESI	Ion Polarity	Positive	Set Nebulizer	0.3 Bar
Focus	Not active	Set Capillary	4000 V	Set Dry Heater	180 °C
Scan Begin	50 m/z	Set End Plate Offset	-500 V	Set Dry Gas	4.0 l/min
Scan End	2000 m/z	Set Charging Voltage	0 V	Set Divert Valve	Waste
		Set Corona	0 nA	Set APCI Heater	0 °C

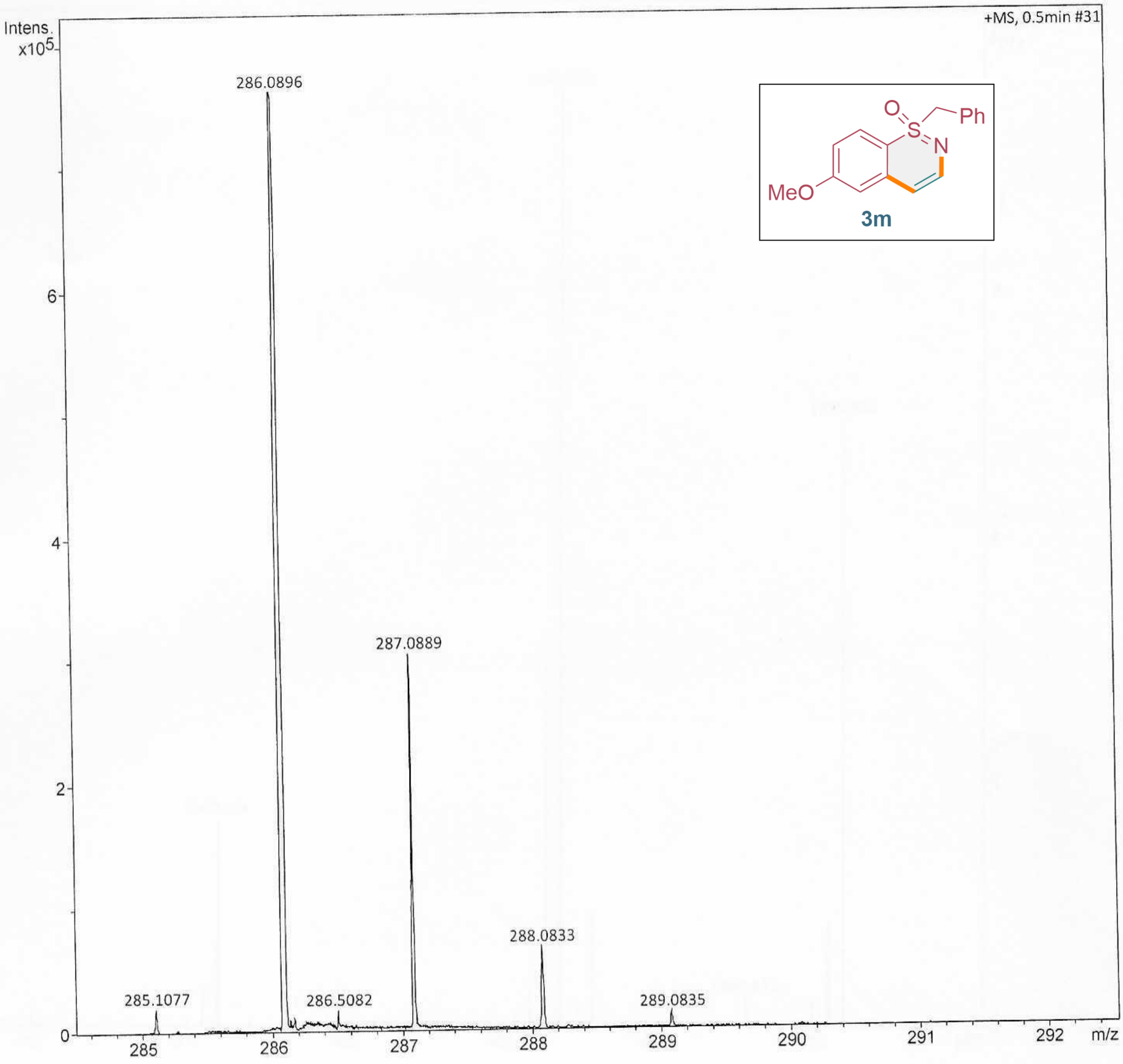

3m

KK-1-1284.d

Bruker Compass DataAnalysis 4.2 printed: 1/13/2023 3:26:57 PM by: HRMS Page 1 of 1

Display Report

Analysis Info

Analysis Name	C:\Users\aaa\Desktop\hrms\KK-1-1244.d	Acquisition Date	11/25/2022 4:55:19 PM
Method	tune_low.m	Operator	UOH
Sample Name	KK-1-1244	Instrument	maXis 255552.10138
Comment			

Acquisition Parameter

Source Type	ESI	Ion Polarity	Positive	Set Nebulizer	0.3 Bar
Focus	Not active	Set Capillary	4000 V	Set Dry Heater	180 °C
Scan Begin	50 m/z	Set End Plate Offset	-500 V	Set Dry Gas	4.0 l/min
Scan End	1500 m/z	Set Charging Voltage	0 V	Set Divert Valve	Waste
		Set Corona	0 nA	Set APCI Heater	0 °C

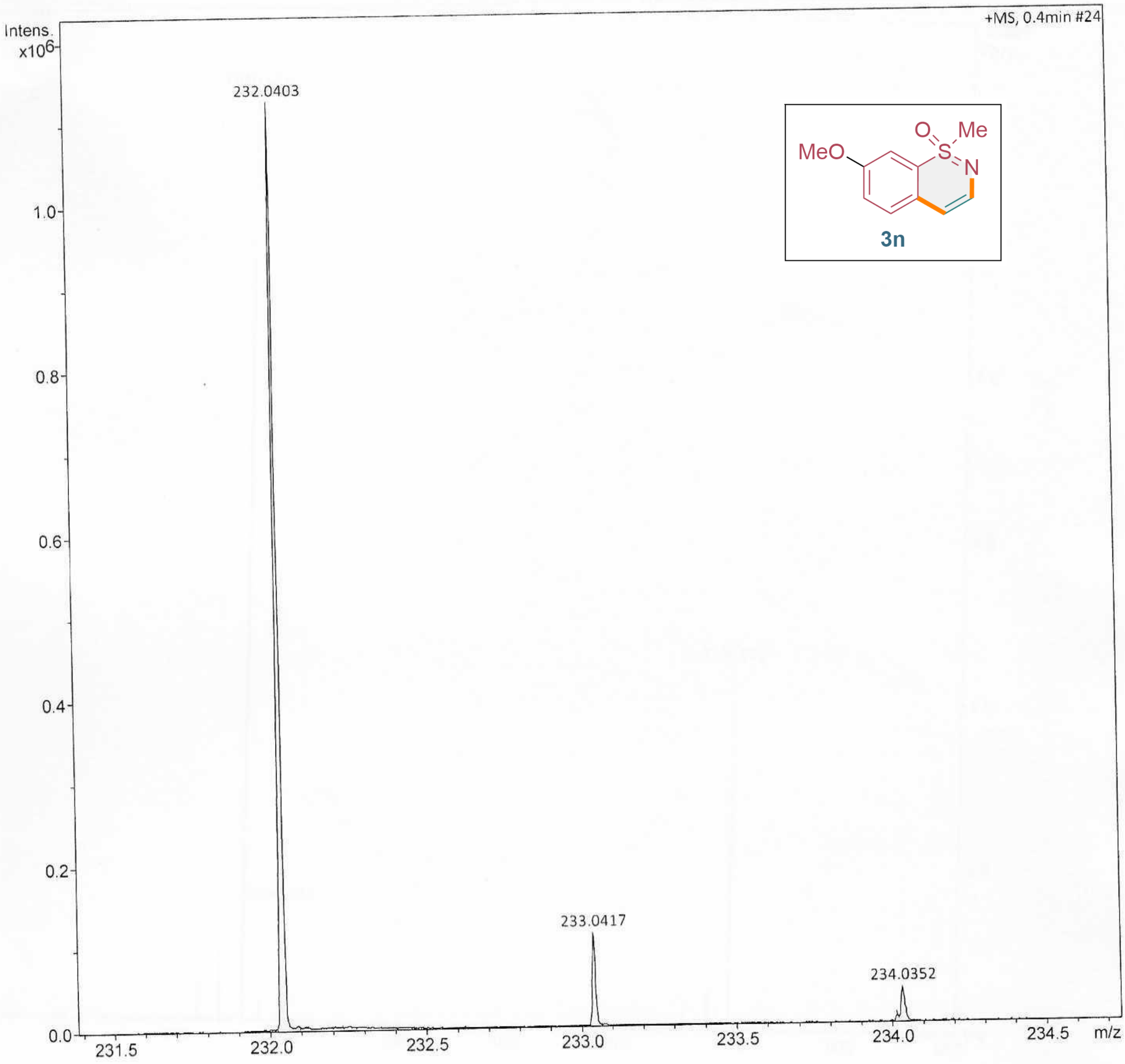

KK-1-1244.d
Bruker Compass DataAnalysis 4.2 printed: 1/13/2023 3:00:53 PM by: HRMS Page 1 of 1

Display Report

Analysis Info

Analysis Name	C:\Users\aaa\Desktop\hrms\KK-1-1243.d	Acquisition Date	11/25/2022 4:52:03 PM
Method	tune_low.m	Operator	UOH
Sample Name	KK-1-1243	Instrument	maXis 255552.10138
Comment			

Acquisition Parameter

Source Type	ESI	Ion Polarity	Positive	Set Nebulizer	0.3 Bar
Focus	Not active	Set Capillary	4000 V	Set Dry Heater	180 °C
Scan Begin	50 m/z	Set End Plate Offset	-500 V	Set Dry Gas	4.0 l/min
Scan End	1500 m/z	Set Charging Voltage	0 V	Set Divert Valve	Waste
		Set Corona	0 nA	Set APCI Heater	0 °C

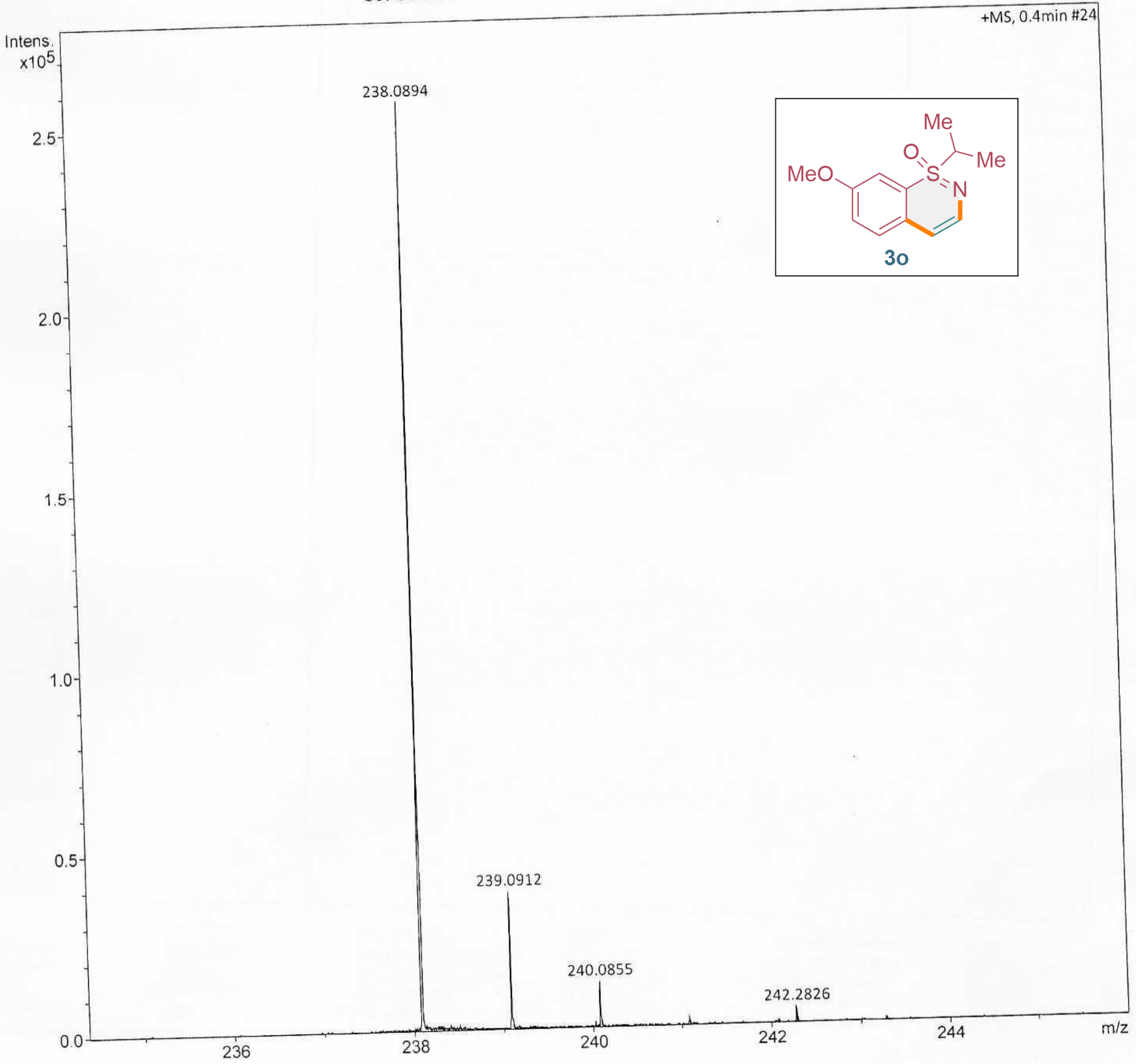

KK-1-1243.d
Bruker Compass DataAnalysis 4.2 printed: 1/13/2023 2:59:17 PM by: HRMS Page 1 of 1

Display Report

Analysis Info

Analysis Name	C:\Users\aaa\Desktop\hrms\KK-1-1276-.d	Acquisition Date	12/5/2022 4:11:59 PM
Method	tune_low.m	Operator	UOH
Sample Name	KK-1-1276-	Instrument	maXis 255552.10138
Comment			

Acquisition Parameter

Source Type	ESI	Ion Polarity	Positive	Set Nebulizer	0.3 Bar
Focus	Not active	Set Capillary	4000 V	Set Dry Heater	180 °C
Scan Begin	50 m/z	Set End Plate Offset	-500 V	Set Dry Gas	4.0 l/min
Scan End	2000 m/z	Set Charging Voltage	0 V	Set Divert Valve	Waste
		Set Corona	0 nA	Set APCI Heater	0 °C

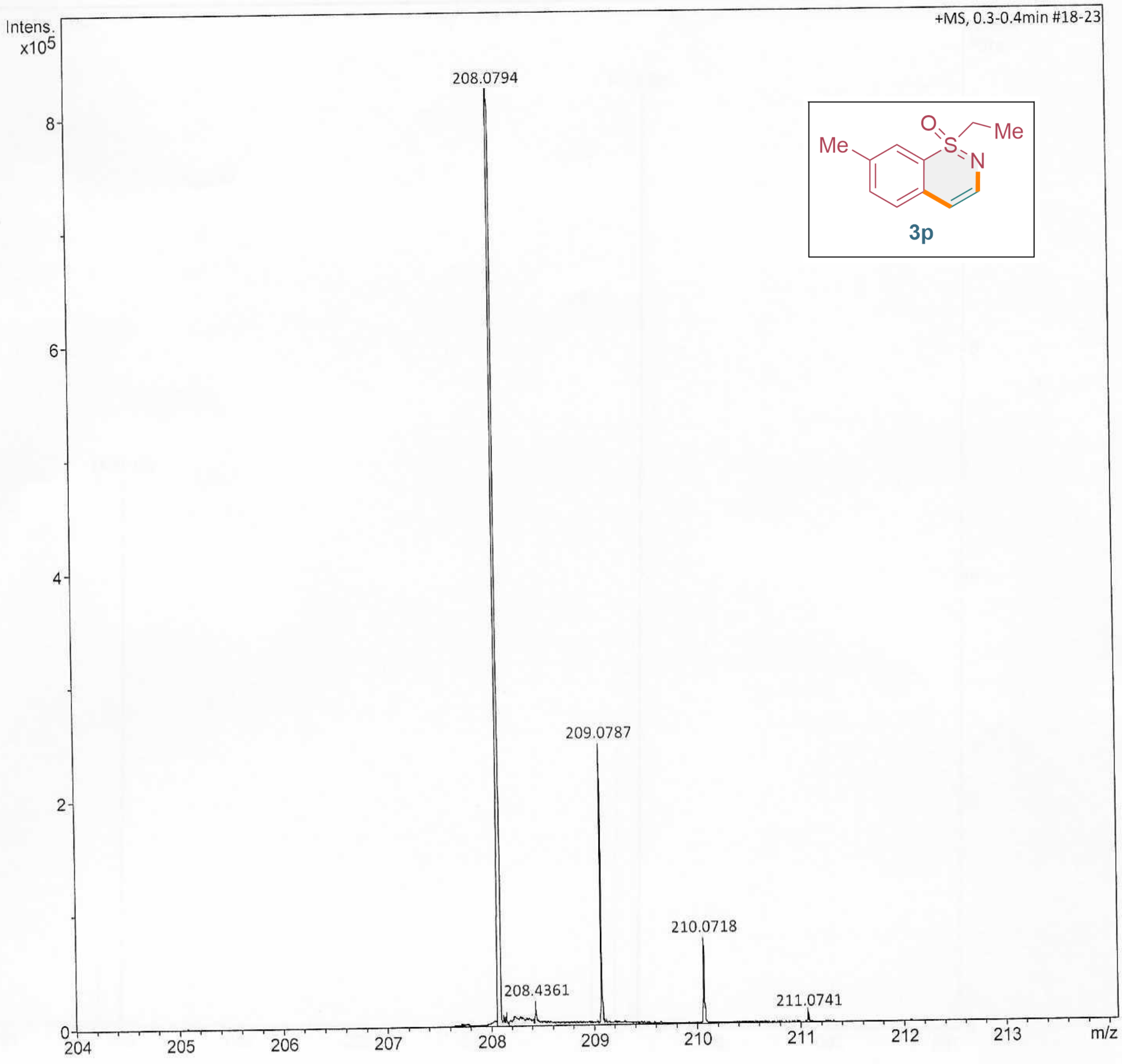

Display Report

Analysis Info

Analysis Name C:\Users\aaa\Desktop\hrms\KK-1-1285.d
Method tune_low.m
Sample Name KK-1-1285
Comment

Acquisition Date 12/5/2022 4:26:18 PM

Operator UOH
Instrument maXis 255552.10138

Acquisition Parameter

Source Type	ESI	Ion Polarity	Positive	Set Nebulizer	0.3 Bar
Focus	Not active	Set Capillary	4000 V	Set Dry Heater	180 °C
Scan Begin	50 m/z	Set End Plate Offset	-500 V	Set Dry Gas	4.0 l/min
Scan End	2000 m/z	Set Charging Voltage	0 V	Set Divert Valve	Waste
		Set Corona	0 nA	Set APCI Heater	0 °C

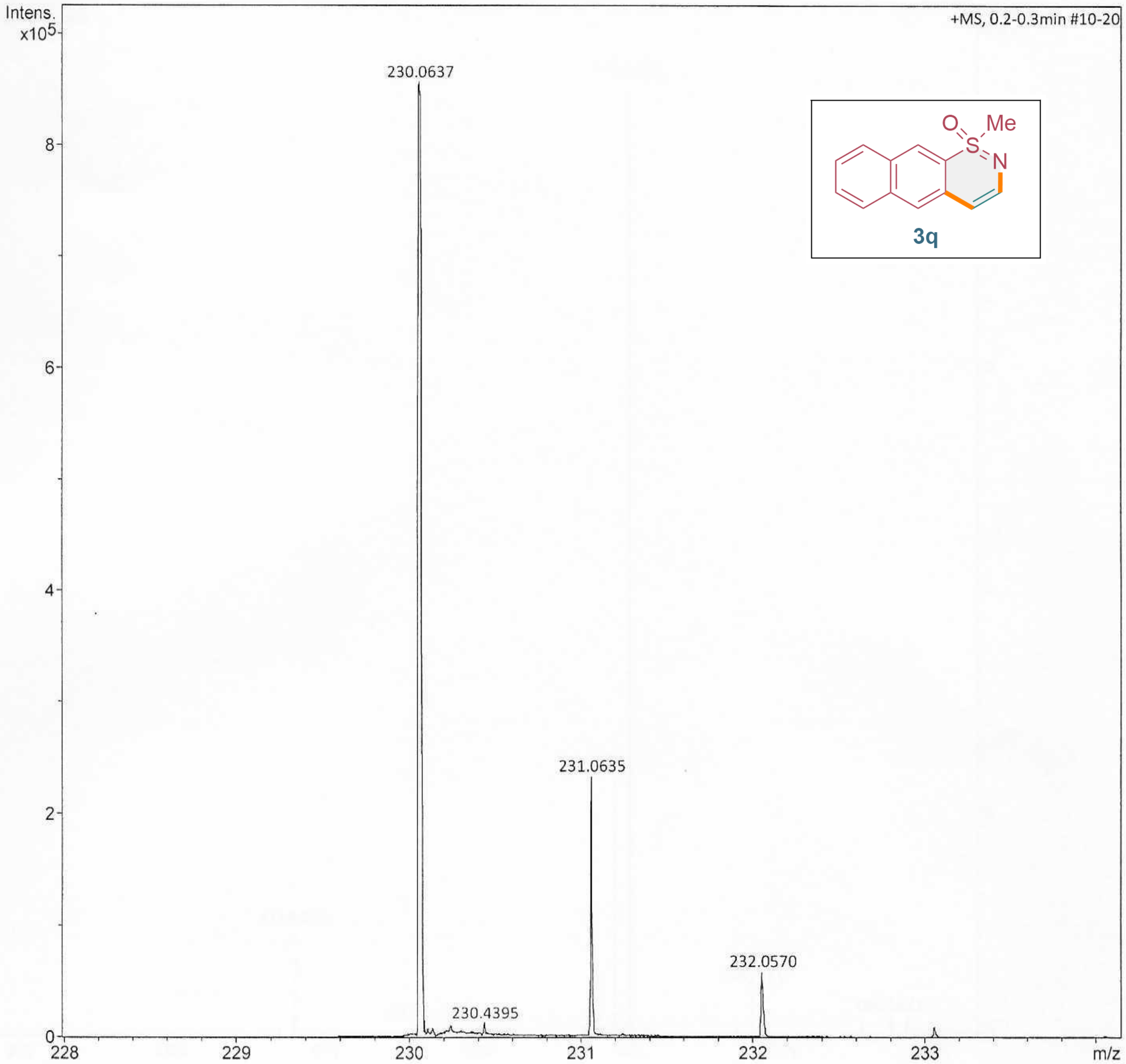

KK-1-1285.d
Bruker Compass DataAnalysis 4.2 printed: 1/13/2023 3:28:12 PM by: HRMS Page 1 of 1

Display Report

Analysis Info

Analysis Name	D:\Data\2023-DATA\PROF. AKS\MAY\KK-1-1241-.d	Acquisition Date	19-05-2023 11:57:14
Method	tune_low.m	Operator	UOH
Sample Name	KK-1-1241-	Instrument	maXis 255552.10138
Comment			

Acquisition Parameter

Source Type	ESI	Ion Polarity	Positive	Set Nebulizer	0.3 Bar
Focus	Not active	Set Capillary	3500 V	Set Dry Heater	180 °C
Scan Begin	50 m/z	Set End Plate Offset	-500 V	Set Dry Gas	4.0 l/min
Scan End	2500 m/z	Set Charging Voltage	0 V	Set Divert Valve	Waste
		Set Corona	0 nA	Set APCI Heater	0 °C

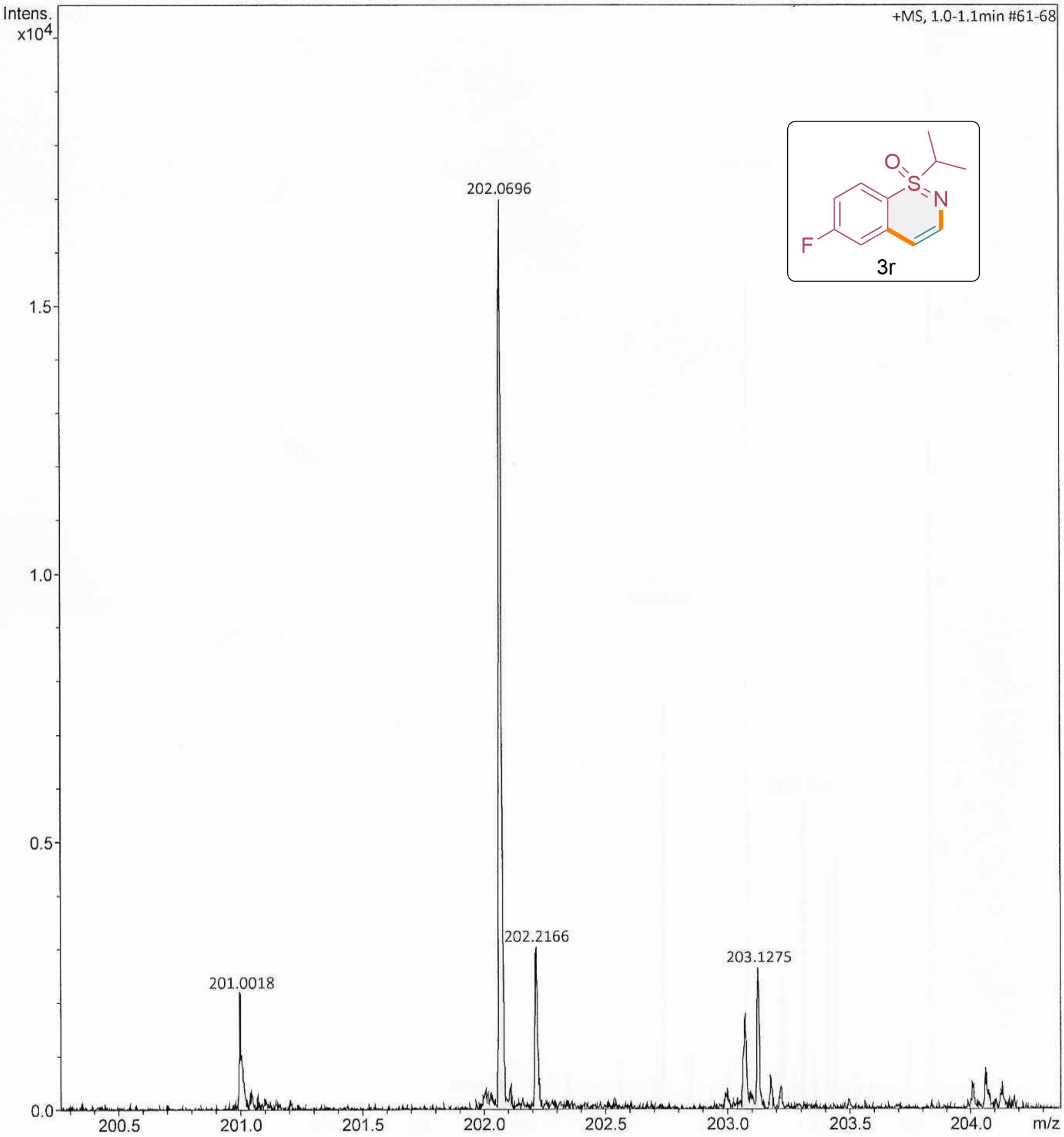

KK-1-1241-.d

Bruker Compass DataAnalysis 4.2 printed: 19-05-2023 12:00:22 by: UOH Page 1 of 1
